# Bioconductor’s EnrichmentBrowser: seamless navigation through combined results of set- & network-based enrichment analysis

**DOI:** 10.1186/s12859-016-0884-1

**Published:** 2016-01-20

**Authors:** Ludwig Geistlinger, Gergely Csaba, Ralf Zimmer

**Affiliations:** Institute of Bioinformatics, Department of Informatics, Ludwig-Maximilians-Universität München, Amalienstrasse 1780333, Munich, Germany

**Keywords:** Gene expression, Differential expression, Pathway analysis, Gene set enrichment, Gene network enrichment

## Abstract

**Background:**

Enrichment analysis of gene expression data is essential to find functional groups of genes whose interplay can explain experimental observations. Numerous methods have been published that either ignore (set-based) or incorporate (network-based) known interactions between genes. However, the often subtle benefits and disadvantages of the individual methods are confusing for most biological end users and there is currently no convenient way to combine methods for an enhanced result interpretation.

**Results:**

We present the EnrichmentBrowser package as an easily applicable software that enables (1) the application of the most frequently used set-based and network-based enrichment methods, (2) their straightforward combination, and (3) a detailed and interactive visualization and exploration of the results. The package is available from the Bioconductor repository and implements additional support for standardized expression data preprocessing, differential expression analysis, and definition of suitable input gene sets and networks.

**Conclusion:**

The EnrichmentBrowser package implements essential functionality for the enrichment analysis of gene expression data. It combines the advantages of set-based and network-based enrichment analysis in order to derive high-confidence gene sets and biological pathways that are differentially regulated in the expression data under investigation. Besides, the package facilitates the visualization and exploration of such sets and pathways.

**Electronic supplementary material:**

The online version of this article (doi:10.1186/s12859-016-0884-1) contains supplementary material, which is available to authorized users.

## Background

Genome-wide gene expression studies with microarrays or RNA-seq typically measure several thousand genes at a time [1]. This makes biological interpretation challenging. To approach this task several statistical filters can be applied to obtain an easier tractable number of genes and to concentrate further investigation effort on genes that are differentially expressed. Subsequently, analysis focuses on whether disproportionately many of the remaining genes belong to known functional sets of genes. Such an enrichment for certain gene functions, sets or pathways immediately generates important hypotheses about underlying mechanisms of e.g. an investigated clinical phenotype.

A recent review divides existing enrichment methods into three generations [2]. The first generation of methods is centered around the traditionally used overrepresentation analysis, which tests based on the hypergeometric distribution whether genes above a predefined significance threshold are overrepresented in functional gene sets [3]. The second generation of methods resolves the restriction to the subset of significant genes, and instead scores the tendency of gene set members to appear rather at the top or bottom of the ranked list of all measured genes [4].

First and second generation methods have in common that they ignore known interactions between genes. Those methods are thus denoted as *set-based* in this manuscript. Methods that do incorporate known interactions belong to the third generation of methods and are denoted as *network-based* in the following (reviewed in [5]).

While each generation is represented by numerous published methods with individual benefits and disadvantages, there is currently no gold standard enrichment method agreed upon. This makes the decision for a particular method intricate. It also leads users, actually intending a better biological understanding of their data, to decide based on criteria not necessarily relating to biological insight such as frequency of usage and ease of application.

Combination of methods has been proven superior to individual methods in different areas of computational biology, as it increases performance [6] and statistical power [7] and biological insights often complement each other [1, 8].

In this article, we propose and implement the straightforward combination of major set- and network-based enrichment methods. We demonstrate that this filters out spurious hits of individual methods and reduces the outcome to candidates accumulating evidence from different methods. This increases the confidence in resulting enriched gene sets, and, thus, substantially enhances the biological interpretation of large-scale gene expression data.

## Implementation

The EnrichmentBrowser is implemented in the statistical programming language R [9] and the package is included in the open-source Bioconductor project [10].

### Overview

**Fig. 1:**
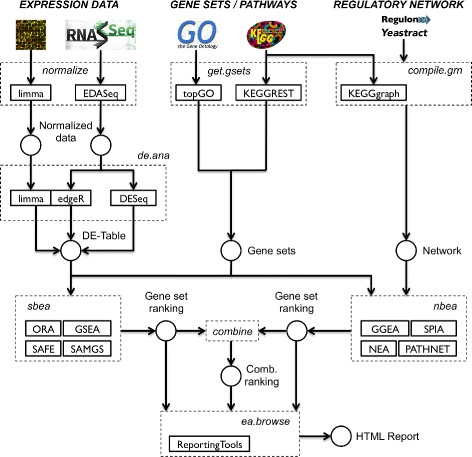
Workflow. Expression data as measured with microarrays or RNA-seq is tested for enrichment of specific functional gene sets, e.g. as defined in the Gene Ontology or the KEGG pathway annotation. Additional information from regulatory networks annotated in specific databases such as the RegulonDB or Yeastract can be exploited. Implemented methods can be carried out individually and combined by selected ranking criteria. Resulting gene set rankings can be browsed as HTML pages allowing detailed inspection (as illustrated in Fig. 2)

Given gene expression data sampling different conditions, specific functional gene sets, and optionally a regulatory network of known interactions between genes, the EnrichmentBrowser performs three essential steps: (1) chosen set- and network-based enrichment methods are executed individually, (2) enriched gene sets are combined by selected ranking criteria, and (3) resulting gene set rankings are displayed as HTML pages for detailed inspection (Fig. 1).

**Fig. 2:**
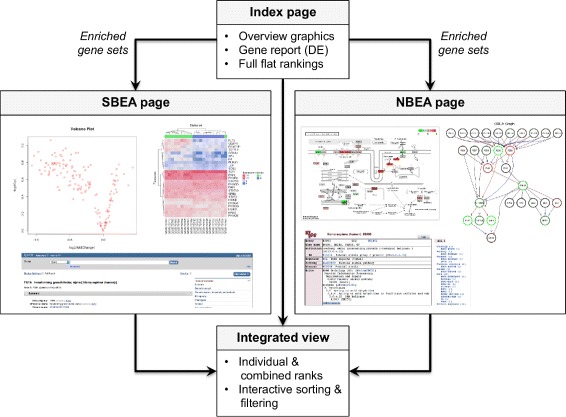
Navigation. Structured access to enrichment analysis results is provided by an index page that links overview graphics, a gene report that includes measures of differential expression for each gene under investigation, and the full flat gene set rankings for each executed method (and, optionally, their combination). In addition, a top table for each method is linked containing detailed set-based (SBEA page) and network-based (NBEA page) views of significant gene sets. The SBEA page for a gene set is composed of (1) an interactive volcano plot (fold change vs. DE *p*-value) allowing immediate identification of significant genes by mouse-over, and (2) two heatmaps displaying the expression of all set genes and the subset of significant genes. The NBEA page illustrates interactions within a gene set by projecting it onto the underlying regulatory network, and for KEGG gene sets by additionally highlighting corresponding pathway maps. Results of method combination are linked in a combined page that displays the combined ranking alongside the individual rankings, and which can be interactively sorted and filtered according to user-selected criteria. See Additional files 2, 3 and 4 for several application examples and the vignette of the EnrichmentBrowser package for details of the various options

### Data preprocessing

The typical starting point for the EnrichmentBrowser is normalized gene expression data. The data are usually microarray intensity measurements or RNA-seq read counts for several thousand genes between two conditions, each represented by a group of samples.

Two inputs are required: (1) the expression matrix, in which each row corresponds to a gene and each column to a sample, and (2) a binary classification vector dividing the samples in cases and controls. In case of paired samples or sample blocks, e.g. indicating different treatments of cases and controls, a vector defining the blocks can optionally be supplied.

While each dataset typically shows individual characteristics that need to be specifically normalized for, the EnrichmentBrowser provides several well-established standard routines for that purpose. This includes within-array/-lane and between-array/-lane normalization for microarray and RNA-seq data based on functionality from the limma [11] and EDASeq [12] package, respectively.

In case of microarray data, once it has been read in, the data is transformed from probe to gene level. This incorporates a mapping from probe to gene identifiers, which is automatically done for recognized platforms (i.e. a corresponding Bioconductor annotation package such as hgu95av2.db [13] exists) or required as a user input otherwise. An important parameter is the summarization method determining, in case of multiple probes for one gene, whether an average value is computed or the probe that discriminates the most between the two sample groups is kept.

### Differential expression

Differences in gene expression between the two sample groups are computed using established functionality from the limma package [11], involving the voom transformation [14] when applied to RNA-seq data. Alternatively, differential expression analysis for RNA-seq data can also be carried out based on the negative binomial distribution with edgeR [15] and DESeq2 [16]. Resulting log2 fold changes and derived *p*-values for each gene can be inspected in several ways (Fig. 2). This includes a gene report that lists the respective measures of differential expression for each gene, and several overview graphics such as:

1. Heatmap: clustered overview of gene expression between the two groups for all genes, and separately, only for significant genes satisfying predefined thresholds of fold change and *p*-value.
2. Volcano plot: fold change versus *p*-value plot that illustrates the correspondence of amount, direction and statistical significance of expression changes (supporting immediate identification and exploration of significant genes by mouse-over and linking to the corresponding gene entries).
3. *P*-value distributions: histogram of raw and adjusted *p*-values.

Multiple testing correction is performed using the p.adjust function from the stats package, which implements several frequently used corrections from which the user can choose (reviewed in [17]). This includes the stringent Bonferroni correction and the less conservative method of Benjamini and Hochberg.

### Enrichment analysis

#### Set-based enrichment analysis

The EnrichmentBrowser implements several ways to assemble the gene sets that should be tested for enrichment. User-defined gene sets can be parsed from suitable formats such as GMT [18] or extracted from pathway XML format [19]. Frequently used organism-specific gene sets from GO [20] and KEGG [21] can be downloaded exploiting functionality from the topGO [22] and KEGGREST [23] package, respectively.

Currently supported are the following major set-based enrichment methods:

1. ORA: Overrepresentation Analysis, simple and frequently used test based on the hypergeometric distribution (reviewed in [3]),
2. SAFE: Significance Analysis of Function and Expression, implements a resampling version of ORA, includes other test statistics such as Wilcoxon’s rank sum, and allows to estimate the significance of gene sets by sample permutation [24],
3. GSEA: Gene Set Enrichment Analysis, frequently used and widely accepted, uses a Kolmogorov-Smirnov statistic to test whether the ranks of the *p*-values of genes in a gene set resemble a uniform distribution [4],
4. SAMGS: Significance Analysis of Microarrays on Gene Sets, extending the SAM method for single genes to gene set analysis [25].

ORA is a first generation method, whereas SAFE, GSEA, and SAMGS belong to the second generation of enrichment methods. The EnrichmentBrowser uses its own implementation of ORA, while it integrates SAFE as implemented in the safe package. Implementations of GSEA and SAMGS are adapted from [26, 27], respectively.

SAFE, GSEA, and SAMGS use sample permutation for estimating the gene set significance, which involves recomputation of their individual local *t*-like statistics for each gene. As this is not *per se* suitable for RNA-seq read count data, the EnrichmentBrowser provides specific local statistics based on the limma/voom-transformed *t*-statistic, the LR-statistic from edgeR, and the Wald-statistic from DESeq. Global statistics for each gene set are accordingly chosen as the KS-statistic (for GSEA), Wilcoxon’s rank sum (for SAFE), and Hotelling’s *T*^2^ (for SAMGS). Permutation testing with selected local and global statistics is carried out using the general framework implemented in the safe package.

#### Network-based enrichment analysis

Gene regulatory networks represent known interactions between genes as derived from specific experiments or compiled from the literature [28]. There are well-studied processes and organisms for which comprehensive and well-annotated regulatory networks are available, e.g. the RegulonDB for *E. coli* [29] and Yeastract for *S. cerevisiae* [30]. While it is recommended to use these specific networks, and the EnrichmentBrowser supports their download and formatting, there are also cases where such a network is not easily available. For these cases the EnrichmentBrowser implements the possibility to compile a network from regulatory interactions annotated in the KEGG database. This incorporates downloading and parsing of the pathways for a selected organism making use of the KEGGREST and KEGGgraph package [31], respectively.

Currently integrated network-based enrichment analysis methods are

1. GGEA: Gene Graph Enrichment Analysis, evaluates consistency of known regulatory interactions with the observed expression data [32],
2. SPIA: Signaling Pathway Impact Analysis, combines ORA with the probability that expression changes are propagated across the pathway topology [33],
3. NEA: Network Enrichment Analysis, applies ORA on interactions instead of genes [34],
4. PathNet: Pathway analysis using Network information, applies ORA on combined evidences of the observed signal and the signal implied by connected neighbors in the network [35].

GGEA is implemented as the default network-based enrichment method of the EnrichmentBrowser and is also incorporated in the network-based visualization of gene sets. SPIA, NEA, and PathNet are integrated as implemented in the SPIA, neaGUI, and PathNet package, respectively.

#### Generic plug-in of additional methods

The goal of the EnrichmentBrowser is to provide the most frequently used enrichment methods. However, it is also possible to exploit its functionality with additional methods not among the currently implemented ones. This requires to implement a function that takes the characteristic arguments eset (expression data), gs (gene sets), alpha (significance level), and in case of network-based enrichment also grn (gene regulatory network). In addition, it must return a vector storing the resulting *p*-value for each gene set in gs.

### Combining results

Different enrichment analysis methods usually result in different gene set rankings for the same dataset. To compare results and detect gene sets that are supported by different methods, the EnrichmentBrowser package allows to combine results from the different set- and network-based enrichment methods. The combination of results yields a new ranking of the gene sets under investigation according to a defined ranking and combination function.

The ranking function determines by which statistic the individual gene set rankings are sorted and which type of ranks are computed. The ranking statistic is typically chosen to be the gene set *p*-value or score (sorted in ascending and descending order, respectively). Predefined rank types include:

1. *Absolute* ranks *r*_*A*_ are assigned from 1 to *n* according to the sorting of the ranking statistic. Intuitively, *n* is identical to the number of gene sets *N*_*GS*_ if the ranking statistic takes a different value for each gene set. As ties can occur, which yields the *same* rank for gene sets with equal value, *n* corresponds to the number of distinct values of the ranking statistic (denoted as *N*_*D*_).
2. To account for a differing number of gene sets in the individual rankings, *relative* ranks *r*_*R*_ can be derived from absolute ranks via *r*_*A*_/*n*·100.
3. Although frequently used to rank gene sets, absolute and relative ranks can be misleading in case of extensive presence of ties. Especially, when comparing a coarse-grained (*N*_*D*_≪*N*_*GS*_) and a fine-grained ranking (*N*_*D*_≈*N*_*GS*_). Here, similar absolute/relative ranks imply a very different meaning. To resolve this, we introduce *competitive* ranks *r*_*C*_ calculated as the percentage of gene sets with a value of the ranking statistic at least as extreme as observed for the gene set to be ranked.

The default ranking function returns competitive ranks based on gene set *p*-values.

The combination function determines how ranks are combined across methods and can be chosen from predefined functions such as mean, median, min, and sum (default). User-defined ranking and combination functions can also be plugged in.

### Visualization and exploration

The standard output of existing enrichment tools is a ranking of the gene sets by the corresponding *p*-value. The EnrichmentBrowser package provides additional visualization and interactive exploration of resulting gene sets far beyond that point. Based on functionality from the ReportingTools package [36], the resulting flat ranking can be accompanied by a HTML report from which each gene set can be inspected in detail (Fig. 2).

Instead of providing individual visualization capabilities for each method, the EnrichmentBrowser implements general set- and network-based visualizations (SBEA and NBEA page). They represent results of methods of the corresponding class, but can also be incorporated independent of the enrichment method executed. It is thus possible to carry out e.g. set-based methods, while including a network-based visualization of significant gene sets in the result report.

The SBEA page is composed as described for the global differential expression report (the set of all measured genes). Thus, a gene set under study is visualized with an interactive volcano plot alongside 2 heatmaps for all and only differentially expressed set members.

The composition of the NBEA page depends on the gene set source and whether a regulatory network is available. For KEGG gene sets, differential expression is visualized directly on the pathways by overplotting the original pathway maps with pathview [37]. In addition, connected subgraphs within a pathway are displayed separately and can be inspected by mouse-over (involves the imageMap function from biocGraph [38]). In case a regulatory network has been provided, gene sets can also be viewed as GGEA graphs. Such a graph displays for a gene set of interest the consistency of each interaction in the network that involves a gene set member. Nodes (genes) are colored according to expression (up-/down-regulated) and edges (interactions) are colored according to consistency, i.e. how well the interaction type (activation/inhibition) is reflected in the correlation of the observed expression of both interaction partners (see the legend in Additional file 1: Figure S1). Although GGEA graphs have been originally implemented for illustrating gene sets according to GGEA, they are apparently useful for depicting mechanisms exploited by network-based methods in general.

The combined result view additionally enables an interactive ranking either based on the combined ranks across methods, or with respect to one of the chosen methods.

## Results and discussion

In the following, we demonstrate the application of the EnrichmentBrowser to microarray and RNA-seq data. Subsequently, we systematically evaluate the individual methods integrated in the package and the effect of combining methods. See Additional file 1 for supplementary material and methods. A comparative evaluation to existing Bioconductor packages and stand-alone tools such as SegMine [39] and graphite web [40] can also be found in Additional file 1.

### Application example 1: ALL microarray data

To demonstrate the functionality of the package for microarray data, we consider expression values of patients suffering from acute lymphoblastic leukemia [41]. A frequent chromosomal defect found among these patients is a translocation, in which parts of chromosome 9 and 22 swap places. This results in the oncogenic fusion gene BCR/ABL created by positioning the ABL1 gene on chromosome 9 to a part of the BCR gene on chromosome 22.

The data is available from Bioconductor in the ALL data package [42] and contains normalized intensity measurements on a log-scale for 12,625 probes across 79 patients. Case and control group were defined according to presence or absence of the BCR-ABL gene fusion. We use functionality of the EnrichmentBrowser for transformation from probe to gene level and differential expression analysis (see Implementation, section *Data preprocessing* and *Differential expression*). Human KEGG pathways were downloaded as gene sets, i.e. ignoring interactions between genes.

We apply ORA to detect overrepresented KEGG pathways using the default significance level *α* of 0.05 (Table 1; and Additional file 2 for the detailed HTML summary). Resulting pathways can be divided in three categories: (1) clearly linked to the phenotype such as transcriptional misregulation in cancer, apoptosis and basal cell carcinoma, (2) unknown and secondary effects of phenotype or treatment like myocarditis, which can be caused by cancer radiation therapy, and (3) clearly irrelevant such as legionellosis (drinking water contamination) and shigellosis (foodborne illness).

**Table 1:** Combination of top ranked gene sets of ORA and GGEA by rank sum (ALL microarray data)

ID	Title	ORA	GGEA	$\sum $
hsa05416	Viral myocarditis	1	1	2
hsa04520	Adherens junction	4	2	6
hsa05217	Basal cell carcinoma	9	3	12
hsa04622	RIG-I-like receptor	2	12	14
hsa04210	Apoptosis	6	10	16
hsa05202	Transcript. misreg. in cancer	7	13	20
hsa05130	Pathogenic E. coli infection	3	-	-
hsa05134	Legionellosis	5	-	-
hsa05131	Shigellosis	8	-	-
hsa05412	Arrhytm. cardiomyopathy	10	-	-
hsa05100	Invasion of epithelial cells	11	-	-
hsa04670	Leukocyte trans. migration	12	-	-
hsa05206	MicroRNAs in cancer	13	-	-
hsa04350	TGF- *β* signaling	-	4	-
hsa04550	Pluripotency of stem cells	-	5	-
hsa05211	Renal cell carcinoma	-	6	-
hsa04310	Wnt signaling	-	7	-
hsa04660	T cell receptor	-	8	-
hsa05144	Malaria	-	9	-
hsa04514	Cell adhesion	-	11	-

Shown are the absolute ranks returned by ORA and GGEA and the resulting rank sum in the last column (see Implementation, section *Combining results*)

We investigate next whether these findings can be explained by known regulatory interactions. This means, whether regulators such as transcription factors and their target genes are expressed in accordance to the connecting regulations. Therefore, we apply GGEA using a network of regulations compiled from the KEGG database. For comparison, we select the same number of gene sets as for ORA from the top of the GGEA ranking (Table 1; and Additional file 2 for the detailed HTML summary).

To identify relevant pathways reported by both methods, we combine the rankings of ORA and GGEA by rank sum, including only gene sets in the intersection of both rankings. This yields a new ranking in which irrelevant pathways such as legionellosis and shigellosis are filtered out (Table 1; and Additional file 2 for the detailed HTML summary). Thus, combining ORA with GGEA yields a ranking reduced to the most plausible pathways, which are supported by several mechanistic explanations in the GGEA graphs.

### Application example 2: TCGA RNA-seq data

The EnrichmentBrowser integrates specific methods for preprocessing and differential expression analysis of RNA-seq data. Accordingly, enrichment methods that rely on sample permutation are adapted to incorporate specific local statistics for recomputation of per-gene differential expression (see Implementation, section *Set-based enrichment analysis*). To demonstrate the functionality, we apply the package for the analysis of RNA-seq data from The Cancer Genome Atlas (TCGA, [43]). We consider here uterine corpus endometrial carcinoma (UCEC), which is one of the most common cancers of the female reproductive system [44].

The data is available from GEO under accession GSE62944 [45] and contains integer sequencing read counts for 554 UCEC tumor and 35 adjacent normal samples. We apply the limma/voom-based differential expression analysis and make use of the KEGG gene set catalogue and regulatory network described in the previous section.

For set-based enrichment analysis, we choose GSEA as it is among the methods specifically adapted in the EnrichmentBrowser for the analysis of RNA-seq data. In addition, we apply PathNet and NEA for network-based enrichment analysis (full rankings can be found in Additional file 3). To investigate the effect of method combination, we combine the 3 individual gene set rankings by rank sum as for the ALL example.

**Fig. 3:**
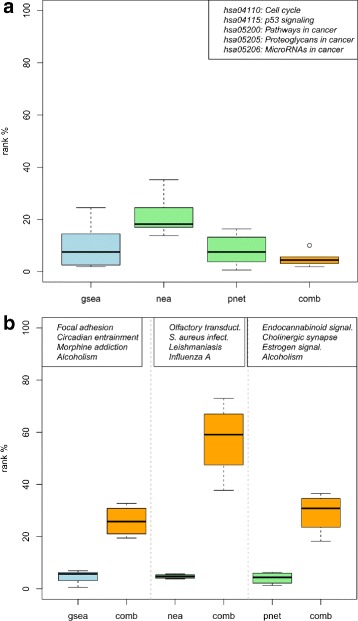
Method combination consolidates cancer-specfic pathways and downgrades unspecific pathways (TCGA RNA-seq data). **a** Competitive rank distributions of selected cancer-specific pathways (listed top right) for GSEA, NEA, and PathNet when applied to the UCEC RNA-seq data from TCGA. The orange rightmost boxplot depicts the corresponding rank distribution when combining the individual rankings by rank sum. Analogously, **b** depicts the rank distributions of selected unspecific pathways (listed at the top of the respective panel) that were top ranked by the 3 individual methods

We find cancer-specific pathways such as *p53 signaling pathway* and *Pathways in cancer* clearly consolidated in the combined ranking (Fig. 3a). On the other hand, unspecific pathways such as *Olfactory transduction* and *Morphine addiction*, which were top ranked by the individual methods, are distinguishably downgraded in the combined ranking (Fig. 3b).

Thus, independent of the expression data type under study (microarray/RNA-seq) and the enrichment methods combined (previously: ORA/GGEA, here: GSEA/NEA/PathNet), the combination has shown to improve individual rankings by increasing confidence in specific target pathways and removing irrelevant pathways from the top of the ranking.

### Systematic evaluation: GEO2KEGG benchmark set

We have observed beneficial effects of combining enrichment methods at the example of specific microarray and RNA-seq datasets. We investigate next whether these effects can be observed systematically when applied to many datasets.

For that purpose, we use a compendium of 27 GEO datasets derived from the *KEGGdzPathwaysGEO* and the *KEGGandMetacoreDzPathwaysGEO* benchmark sets [46, 47]. See Additional files 1 and 4 for details. These datasets have been specifically selected as they investigate a certain human disease for which a corresponding KEGG pathway exists (e.g. Alzheimer’s disease). These pathways are thus denoted as the target pathways in the following.

We investigate first how well the individual set- and network-based methods detect the target pathways and, subsequently, whether the detection can be improved by combining methods.

#### Individual methods

**Fig. 4:**
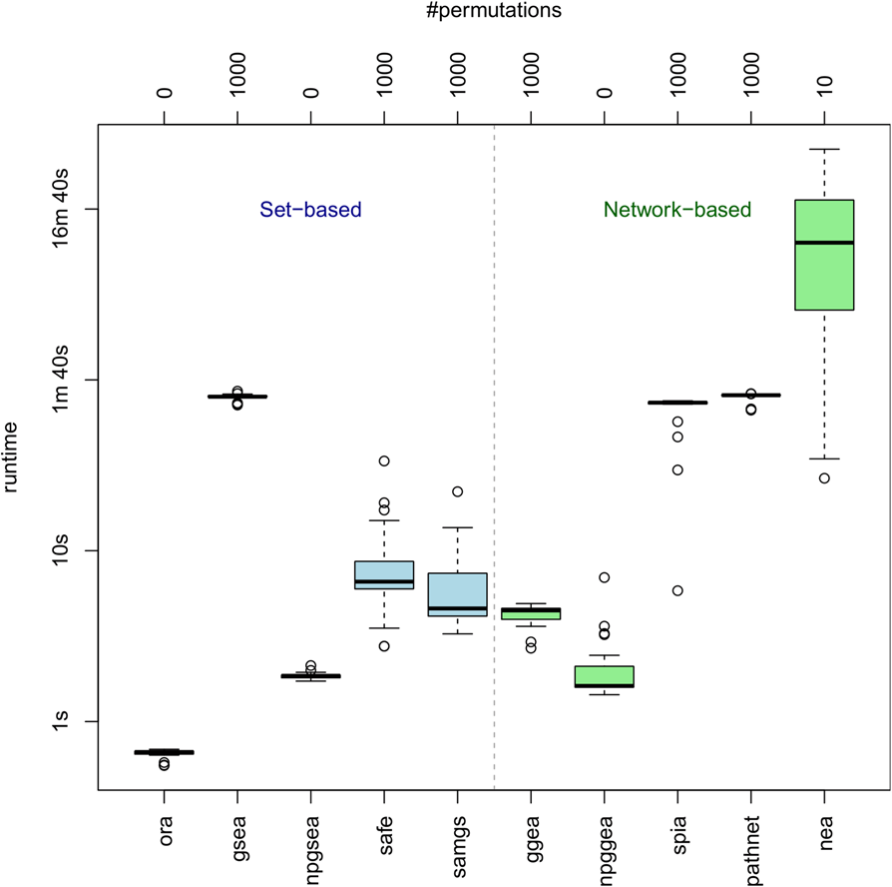
Runtime. Shown are the distributions of the elapsed processing times (*y*-axis, log-scale) when applying the enrichment methods indicated on the bottom *x*-axis to the 27 datasets of the GEO2KEGG benchmark set. The *x*-axis on top of the plot indicates the number of permutations that have been used to estimate gene set significance. Elapsed runtime of NEA when using 100 and 1000 permutations, respectively, are shown in Additional file 1: Figure S2

When applying the 8 methods to the 27 datasets of the GEO2KEGG benchmark set, an issue of practical relevance is runtime (Fig. 4). As expected, runtime of the methods depends mainly on whether permutation testing is used to estimate gene set significance, and whether this is efficiently implemented.

ORA, applying the hypergeometric test without permutation, can thus be performed with almost no effort, displaying a constant runtime of around half a second per dataset. GSEA, applied with a default of 1000 permutations, is slower by two orders of magnitude taking around 1 *min* 40 *sec* per dataset. It should be mentioned that the original GSEA R script [26], which has been straightforward translated from Java, is considerably slower. The version integrated in the EnrichmentBrowser has been substantially optimized by making use of vectorized calculations. SAFE and SAMGS, taking typically 5–10 *sec* depending on the dataset, although methodically similar to GSEA are much faster as they do not rely on the computationally intensive cumulative KS-statistic. However, using the npGSEA permutation approximation [48] reduces the runtime of GSEA to ≈2 *sec* per dataset.

Concerning the network-based methods, SPIA and PathNet display similar runtime as observed for permutation-based GSEA. NEA seems to be inefficiently implemented, requiring already for 10 permutations ≈13 *min* on average and up to 2 1/2 days for 1000 permutations (Additional file 1: Figure S2). On the other hand, the code of GGEA has been highly optimized and yields short computation times. The permutation-based version takes ≈4 *sec* per dataset. Using a similar permutation approximation as for GSEA reduces the runtime of GGEA to ≈2 *sec* per dataset.

Resulting gene sets returned by the enrichment methods are typically ranked by gene set *p*-value. However, given that a method can return the same *p*-value for more than one gene set impairs a straightforward ranking. This applies especially to permutation *p*-values, which typically lack a suitable granularity [48]. We have thus introduced competitive ranks, defined as the percentage of gene sets with a *p*-value at least as extreme as observed for the gene set to be ranked (see Implementation, section *Combining results*).

**Fig. 5:**
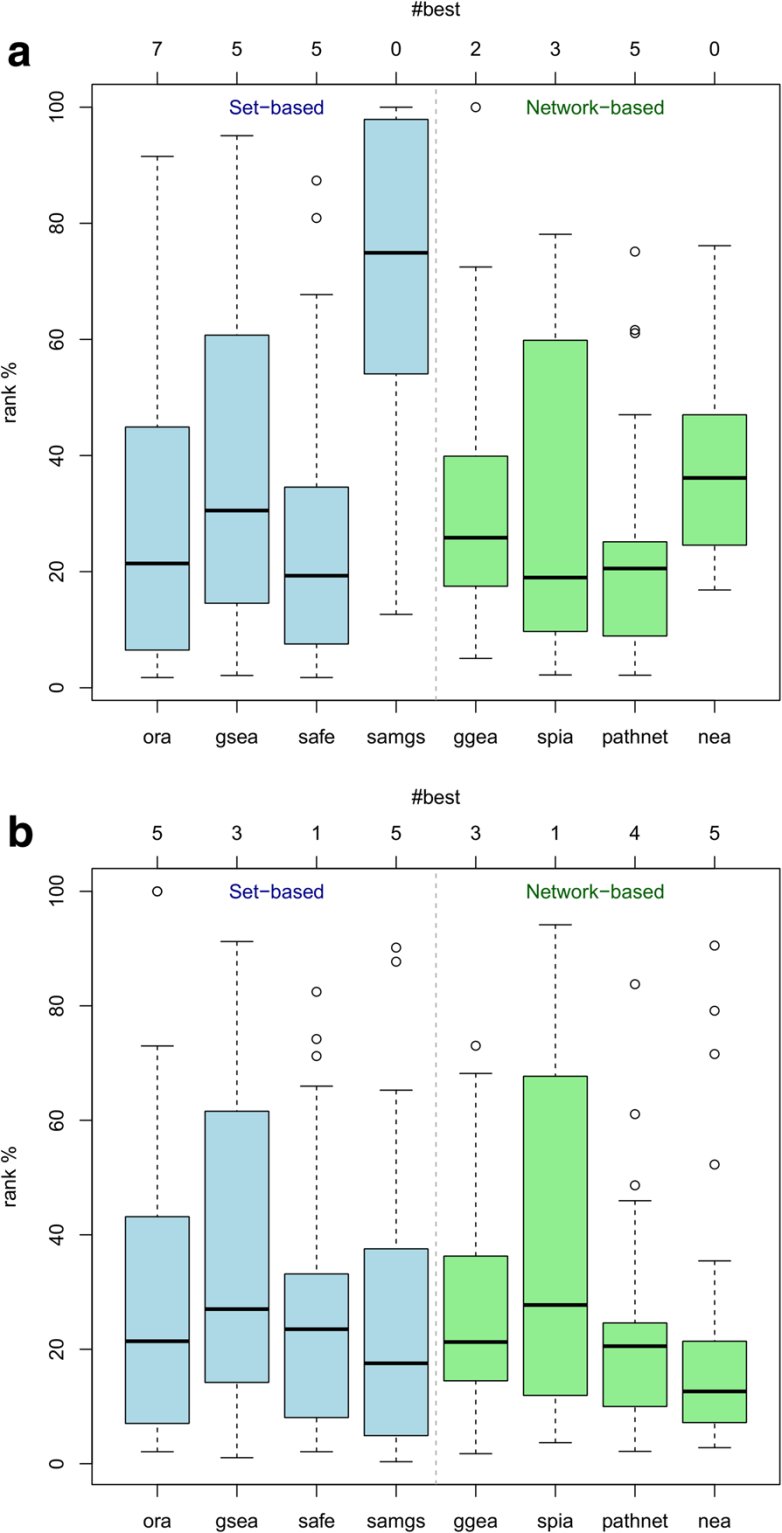
Evaluation of individual methods on the GEO2KEGG benchmark set. Depicted are competitive rank distributions of the KEGG target pathways according to **(a)** gene set *p*-value and **(b)** gene set score of the individual methods when applied to the 27 GEO datasets. The *x*-axis on top of both plots indicates the number of datasets for which the corresponding method resulted in the best ranking (among methods) of the target pathway. As an example, the leftmost blue boxplot in **(a)** shows for ORA a median rank of ≈20 %, i.e. ORA returned for half of the datasets a competitive rank below 20 %. Depicted on top, *#best*=7 means that ORA returned for 7 datasets the best ranking (among methods) for the target. Detailed rank distributions for each dataset can be found in Additional file 1: Figure S3 and S4

Competitive rank distributions of the target pathways when applying the 8 methods to the 27 datasets of the GEO2KEGG benchmark set are shown in Fig. 5a. With the exception of SAMGS and, to a lesser extent, NEA, *p*-value based rankings of the remaining 6 methods appear to well discover the relevance of the target pathways. Their rank distributions are clearly shifted towards the top of the ranking (median ranging from 19 % for SPIA to 30 % for GSEA). This can be interpreted as a clear sign for relevance of the target pathways for the corresponding datasets. However, this also shows that there is no clearcut relation between target pathway and dataset as it would be indicated by throughout top rankings of the target pathways. This is presumably due to interfering issues inherent to KEGG such as incompleteness of the pathway definition as well as overlap and crosstalk between pathways [46, 49].

Nevertheless, there are several notable observations that can be made here: (1) GSEA, typically assumed superior to ORA by incorporating all measured genes, does not display an increased potential for discovering the target pathways. This indicates that most of the variance observed for these sets is explained by genes that are significantly differentially expressed. (2) Similarly, rank distributions of the network-based methods do not deviate significantly from the set-based methods, although they are typically assumed to better reflect the regulatory mechanisms within sets. This indicates that the KEGG network used here is of limited suitability. As it predominantly contains protein-protein interactions and only a small fraction of transcriptional regulatory interactions, quantitative changes in transcriptomic data reflecting effects of interactions are presumably rare. (3) Detailed inspection of the rank distributions shown in Fig. 5a reveals that none of the methods is best suited for all datasets (Additional file 1: Figure S3). There are ≥2 datasets for each method yielding the best ranking (among methods) of the target pathway. (4) SAMGS returns typically a *p*-value of zero for 70–80 % of the gene sets tested, rendering this *p*-value a measure not suitable for ranking. As permutation *p*-values should never be zero [50], usage of this *p*-value is also not recommended for expressing statistical significance. Similarly, permutation *p*-values reported by NEA, although obtained with large computational effort (see runtime discussed earlier), appear also not suitable for ranking.

Given the observed issues for NEA and SAMGS when ranking results by gene set *p*-value, we also ranked the target pathways by gene set score (Fig. 5b). While ranking of the other 6 methods remained almost invariant (GGEA slightly better, SAFE and SPIA slightly worse), this substantially improved rankings returned by SAMGS and NEA. However, inspecting the rank distributions for each dataset in more detail (Additional file 1: Figure S4) showed again that no single method consistently returned best rankings. We observed at least one dataset for each method with the best ranking (among methods) of the corresponding target pathway.

#### Method combination

**Fig. 6:**
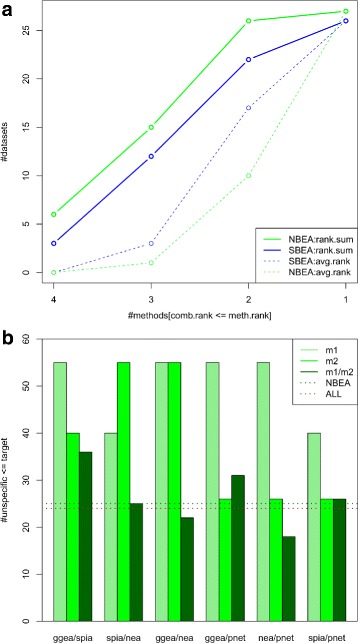
Combination of methods improves individual rankings on the GEO2KEGG benchmark set. **a** Combined ranks by rank sum (solid lines) were computed for the SBEA-combination of the 4 set-based methods (blue) and the NBEA-combination of the 4 network-based methods (green). Depicted is the number of GEO datasets (*y*-axis) for which the combination yielded a ranking of the corresponding target pathway, which was at least as good as obtained from *x* of the individual methods. As an example, the green point at *x*=3 and *y*=15 indicates that the NBEA-combination returned for 15 of the 27 datasets (55.6 %) a ranking of the target as good or better as obtained for 3 of the 4 individual methods (i.e. only one method yielded a ranking better than the combination). For comparison, the dashed lines depict corresponding results when, instead of re-ranking by rank sum (solid lines), ranks are averaged across methods. **b** shows the total number of unspecific pathways ranked at least as good as the target pathway (*y*-axis) for the 4 network-based methods and each pairwise combination. Unspecific pathways are defined in the main text and listed in Additional file 1: Table S1. Corresponding values for the NBEA-combination of the 4 network-based methods and the ALL-combination of all 8 methods (4 set- and 4 network-based) are indicated with the green and the brown dotted line, respectively

Motivated by the results observed for individual methods in the previous section, we investigated next the effect of combining results of methodically similar methods. Therefore, we computed combined ranks by rank sum for the 4 set- (SBEA) and the 4 network-based (NBEA) methods (Fig. 6a).

The NBEA-combination yielded for 6 of the 27 datasets (SBEA: 3 datasets) a ranking of the corresponding target pathway, which was at least as good as obtained for all 4 individual methods. Importantly, the combination returned for almost all datasets (SBEA: 26 datasets, NBEA: all 27 datasets) a ranking of the corresponding target pathway, which was at least as good as obtained for 1 of the 4 methods. This indicates that combining methods is typically safe, i.e. is rarely worse than the worst individual ranking. On the other hand, the combination resulted in many cases in improved rankings of relevant target pathways. In addition, re-ranking by rank sum yielded significantly better ranks of the target pathways as obtained by simply averaging individual ranks across methods (compare dashed and solid lines in Fig. 6a).

As observed for the microarray and RNA-seq application example, combination allowed to filter out, i.e. downgrade irrelevant pathways reported by individual methods. Therefore, we counted for all pairwise combinations of the 4 network-based methods the total number of unspecific pathways ranked at least as good as the target pathway (Fig. 6b). A pathway was denoted as unspecific, if it did not share any genes with the target pathways, and the pathway title suggested no relevance for the diseases studied in the GEO2KEGG benchmark set (such as *Synaptic vesicle cycle* and *Vitamin digestion*; see Additional file 1: Table S1). We found that all 6 pairwise combinations considerably reduced the number of unspecific pathways ranked as good or better than the target. Considering the GGEA/NEA-combination the number of unspecific pathways was reduced by >50 % for both methods. On the other hand, combination with PathNet that displayed the least unspecific pathways, allowed to downgrade up to 70 % unspecific pathways for NEA (while decreasing the number for PathNet even further). We also computed all pairwise combinations of the 4 set-based methods, the effect was however not as pronounced as observed for the network-based methods (Additional file 1: Figure S5).

In summary, given the heterogeneous individual rankings that we observed for the GEO2KEGG benchmark set, combining methods can, of course, not in all situations be expected to be superior to applying individual methods. However, we observed that the combination rarely results in loss of crucial information, but rather yielded in many cases a gain in confidence of relevant pathways while reducing the fraction of unspecific pathways.

## Conclusion

The ongoing development of individual enrichment methods impairs a straightforward decision for the method of choice. The EnrichmentBrowser offers a pragmatic solution by enabling the execution and combination of several major set- and network-based enrichment methods. Whereas no single method is best suited for all application scenarios, this allows to use them all at the same time facilitating a simple direct comparison of the results. It seamlessly displays inconsistencies reported by the applied methods, which makes the user aware that interpretation is needed and has to be done with care in order to derive valid conclusions. The combination can help to avoid misleading results of individual methods by removal of irrelevant gene sets, thus, reducing the outcome to candidates accumulating evidence from different methods. Of course, such consensus combinations come at the cost of less sensitivity but the EnrichmentBrowser does not prohibit that the user accepts non-consensus results from individual methods after careful assessment nevertheless. Detailed investigation of obtained gene sets and pathways is supported by accompanying comprehensive visualization and exploration capabilities. This exceeds considerably the functionality of available tools and we expect users and developers to likewise benefit from it.

## Availability

- **Project name:**EnrichmentBrowser
- **Project home page:**http://bioconductor.org/packages/EnrichmentBrowser
- **Operating system(s):** Platform independent
- **Programming language:**R
- **Other requirements:** Bioconductor
- **License:** Artistic-2.0
- **Any restrictions to use by non-academics:** none

## Additional files

Additional file 1 **Supporting Information.** Supplementary material & methods, comparative evaluation to existing tools, supplementary figures and tables. (PDF 921 kb)

Additional file 2 **EnrichmentBrowser output (ALL microarray data).** Unzip and open the contained index.html in the browser to view the contents of this file (tested with Firefox 39.0). (ZIP 2775 kb)

Additional file 3 **EnrichmentBrowser output (TCGA RNA-seq data).** Unzip and open the contained index.html in the browser to view the contents of this file (tested with Firefox 39.0). (ZIP 7116.8 kb)

Additional file 4 **GEO2KEGG target pathways.** Unzip and open the contained index.html in the browser to view the contents of this file (tested with Firefox 39.0). ZIP 4597.76 kb
