# Supplementary material for: Bioconductor’s EnrichmentBrowser: seamless navigation through combined results of set- & network-based enrichment analysis
Source: BMC Bioinformatics. 2016 Jan 20;17:45. doi: 10.1186/s12859-016-0884-1 (PMC4721010; doi:10.1186/s12859-016-0884-1)
Supplement: Supplementary file 1 — Supporting Information. Supplementary material & methods, comparative evaluation to existing tools, supplementary figures and tables. (PDF 921 kb) [file 12859_2016_884_MOESM1_ESM.pdf]

Supporting Information

Contents

1 Supplementary Material and Methods **2**
1.1 Application example 1: ALL microarray data 2
1.2 Application example 2: TCGA RNA-seq data 3
1.3 Systematic evaluation: GEO2KEGG benchmark set 3

2 Comparative evaluation **4**
2.1 Existing Bioconductor packages 4
2.2 Stand-alone tools 4

List of Figures

S1 GGEA graph legend 6
S2 NEA runtime 6
S3 GEO2KEGG ranks (based on *p*-value) 7
S4 GEO2KEGG ranks (based on score) 7
S5 Unspecific pathways (SBEA-combinations) 8

List of Tables

S1 Unspecific pathways (GEO2KEGG) 8

1 Supplementary Material and Methods

Analysis was carried out using the `EnrichmentBrowser` package, version 1.2.2.

1.1 Application example 1: ALL microarray data

The `ALL` data package, version 1.11.0, was downloaded from `Bioconductor` and loaded in `R`

```
> library(ALL)
> data(ALL)
```

B-cell ALL patients with and without the BCR/ABL fusion were selected via

```
> ind.bs <- grep("^B", ALL$BT)
> ind.mut <- which(ALL$mol.biol %in% c("BCR/ABL", "NEG"))
> sset <- intersect(ind.bs, ind.mut)
> eset <- ALL[, sset]
```

Transformation from probe to gene level was carried out

```
> gene.eset <- probe.2.gene.eset(eset)
```

and differential expression analysis via

```
> pData(gene.eset)$GROUP <- ifelse(eset$mol.biol == "BCR/ABL", 1, 0)
> gene.eset <- de.ana(gene.eset)
```

Human KEGG pathways were downloaded as gene sets (i.e. ignoring interactions between genes)

```
> hsa.gs <- get.kegg.genesets("hsa")
```

and subjected to the enrichment analysis.
ORA was executed with the command

```
> sbea.res <- sbea(method="ora", eset=gene.eset, gs=hsa.gs, perm=0)
```

The gene regulatory network was compiled with

```
> hsa.grn <- compile.grn.from.kegg("hsa")
```

and used as input for GGEA

```
> nbea.res <- nbea(method="ggea", eset=gene.eset, gs=hsa.gs, grn=hsa.grn)
```

Results were combined by rank sum of the absolute ranks obtained for ORA (ranked by nominal gene set p-value) and GGEA (ranked by gene set score).

```
> res.list <- list(sbea.res, nbea.res)
> comb.res <- comb.ea.results(res.list,
+     rank.col=c("P.VALUE", "NORM.SCORE"),
+     decreasing=c(FALSE, TRUE),
+     rank.fun="abs.ranks",
+     comb.fun="sum"))
```

1.2 Application example 2: TCGA RNA-seq data

GSE62944 was downloaded from GEO, and UCEC tumor and adjacent normal samples were selected according to

`GSE62944_06_01_15_TCGA_24_CancerType_Samples.txt.gz`
`GSE62944_06_01_15_TCGA_24_Normal_CancerType_Samples.txt.gz`

Integer read counts for these 554 + 35 = 589 samples were extracted from

`GSM1697009_06_01_15_TCGA_24.normal_Rsubread_FeatureCounts.txt.gz`
`GSM1536837_06_01_15_TCGA_24.tumor_Rsubread_FeatureCounts.txt.gz`

HGNC gene symbols were mapped to Entrez gene IDs. To avoid biasing the analysis with genes of low read count, we excluded genes with a read count sum across samples below 50.
GSEA, NEA, and PathNet were applied with default settings using the KEGG gene sets and regulatory network defined earlier for the ALL application example. Competitive ranks were computed according to nominal gene set p-values (for GSEA and PathNet) and gene set score (for NEA), respectively.

1.3 Systematic evaluation: GEO2KEGG benchmark set

The `KEGGdzPathwaysGEO`, version 1.7.0, and `KEGGandMetacoreDzPathwaysGEO`, version 0.103.0, data packages were downloaded from `Bioconductor`. In total, both packages contained 24 + 18 = 42 GEO datasets. Considering only datasets with a target pathway from KEGG yielded a subset of 34 datasets. Assigned target pathways were visualized as depicted in Additional file 4. We accordingly excluded the following 7 datasets

`GSE20153, GSE20291, GSE6956AA, GSE6956C, GSE781, GSE8762, GSE16759`

from further analysis as the corresponding target pathways did not show sufficient evidence for differential expression. This filtering step was done to ensure the qualification of the target pathways for the selected datasets, and to enable the inclusion of ORA, SPIA, and NEA in the evaluation (as these methods require differentially expressed genes as input).
KEGG gene sets and regulatory network were defined as described for the ALL application example. Except for ORA, all enrichment methods were applied with a default of 1000 permutations. Nominal p-values were used for the rankings depicted in Figure S3 and Figure 5a of the main manuscript. Rankings according to gene set score depicted in Figure S4 and Figure 5b of the main manuscript were computed using the following scores:

1. ORA: number significantly differentially expressed genes / number genes (in gene set),
2. GSEA: normalized enrichment score (`NES`),
3. SAFE: global statistic (Wilcoxon's rank sum) / number genes (in gene set),
4. SAMGS: global statistic (sum of squares of per-gene SAM t-like statistic) / number genes (in gene set),
5. GGEA: normalized consistency score (`NORM.SCORE`),
6. SPIA: perturbation accumulation score (t_A),
7. PathNet: number genes with significant combined evidence / number genes (in gene set),
8. NEA: Z-score.

2 Comparative evaluation

2.1 Existing Bioconductor packages

The `EnrichmentBrowser` provides functionality, which is not covered by already existing packages. This includes the implementation of GGEA shown to improve consistency and explainability of existing enrichment methods [1]. Accompanying GGEA graphs display an intuitive network-based data view extending existing visualizations, which focus predominantly on expression changes of the genes (nodes). GGEA graphs visualize in addition the corresponding effects induced on the interactions (edges).
The combination of enrichment methods is a characteristic feature of the `EnrichmentBrowser`. It allows to interactively inspect the ranking of one or more methods with respect to another method. While this is valuable for biologists for data interpretation, it is as useful for bioinformaticians for design and evaluation of new methods.
On the other hand, the `EnrichmentBrowser` makes intensive use of established functionality of existing packages (see also the main manuscript, section *Implementation*). The full list of all exploited packages can be found on the homepage of the package. There are several recent packages with alternative implementations for certain parts of the `EnrichmentBrowser`. Among them is `graphite` for compiling regulatory networks from KEGG pathways [2] and `PADOG` for comparing gene set rankings against a reference method [3]. These packages are considered for integration in future versions of the `EnrichmentBrowser`.

2.2 Stand-alone tools

The inclusion in Bioconductor has several beneficial aspects: peer-review of package contents by an expert of the Bioconductor team before acceptance, implication of commitment to package maintenance, a periodic release cycle of major updates, and community support for users (via mailing lists). These standards are typically not met to this extent by stand-alone tools. We nevertheless compare the package with two recently published stand-alone tools aiming at a similar functionality.
`SegMine` [4] implements a workflow for semantic microarray analysis as part of the required `Orange4WS` environment [5]. Its application is restricted to three organisms (human, mouse, rat), three gene set definitions (GO, KEGG, Entrez) and three set-based enrichment methods (ORA, GSEA, PAGE [6]). Resulting p-values of the three methods can be combined by a weighted average, which is however statistically discouraged [7]. Interactions between genes are indirectly used for defining and visualization of the gene sets, but are not incorporated in the enrichment analysis itself.
A tool that can be directly executed through a web interface and that allows set- and network-based enrichment analysis is `graphite web` [8]. However, its application is restricted to three organisms (human, mouse, fly), two pathway databases (KEGG and Reactome), three set-based (ORA, GSEA, GLOBALTEST [9]) and two network-based enrichment methods (SPIA and CLIPPER [10]). Methods cannot be combined and visualization and exploration of results is limited to Cytoscape-based graphs in which nodes are colored according to differential expression. Further visualization of effects on the edges and exploration of the gene sets is omitted.
The restrictions of `SegMine` and `graphite web` do not apply to the `EnrichmentBrowser`, which works independent of the organism under investigation. While it extensively supports gene set and pathway definitions according to KEGG, user-defined input in respective gene set and pathway file formats can be also processed. Analogously, the `EnrichmentBrowser` predefines frequently used set- and network-based enrichment methods from which the user can choose, yet it is also possible to seamlessly plugin one's own enrichment method.

References

[1] Geistlinger, L., Csaba, G., Küffner, R., Mulder, N., Zimmer, R.: From sets to graphs: towards a realistic enrichment analysis of transcriptomic systems. Bioinformatics **27(13)**, 366–73 (2011)

[2] Sales, G., Calura, E., Cavalieri, D., Romualdi, C.: graphite - a bioconductor package to convert pathway topology to gene network. BMC Bioinformatics **13**, 20 (2012)

[3] Tarca, A.L., Draghici, S., Bhatti, G., Romero, R.: Down-weighting overlapping genes improves gene set analysis. BMC Bioinformatics **13**, 136 (2012)

[4] Podpecan, V., Lavrac, N., Mozetic, I., Novak, P.K., Trajkovski, I., *et al.*: Segmine workflows for semantic microarray data analysis in orange4ws. BMC Bioinformatics **12**, 416 (2011)

[5] Podpecan, V., Zemenova, M., Lavrac, N.: Orange4ws environment for service-oriented data mining. The Computer Journal **55**, 82–98 (2012)

[6] Kim, S.Y., Volsky, D.J.: Page: parametric analysis of gene set enrichment. BMC Bioinformatics **6**, 144 (2005)

[7] Kim, S.C., Lee, S.J., Lee, W.J., Yum, Y.N., Kim, J.H., *et al.*: Stouffer's test in a large scale simultaneous hypothesis testing. PLoS One **8(5)**, 63290 (2013)

[8] Sales, G., E, C., Martini, P., Romualdi, C.: Graphite web: Web tool for gene set analysis exploiting pathway topology. Nucleic Acids Res **41(Web Server issue)**, 89–97 (2013)

[9] Goeman, J.J., van de Geer, S.A., de Kort, F., van Houwelingen, H.C.: A global test for groups of genes: testing association with a clinical outcome. Bioinformatics **20(1)**, 93–9 (2004)

[10] Martini, P., Sales, G., Massa, M.S., Chiogna, M., C, R.: Along signal paths: an empirical gene set approach exploiting pathway topology. Nucleic Acids Res **41(1)**, 19 (2013)

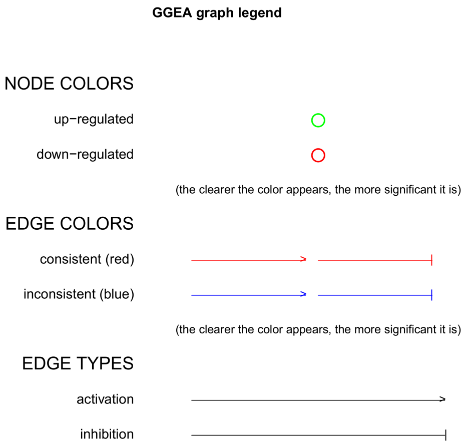

Figure S1: GGEA graph legend.

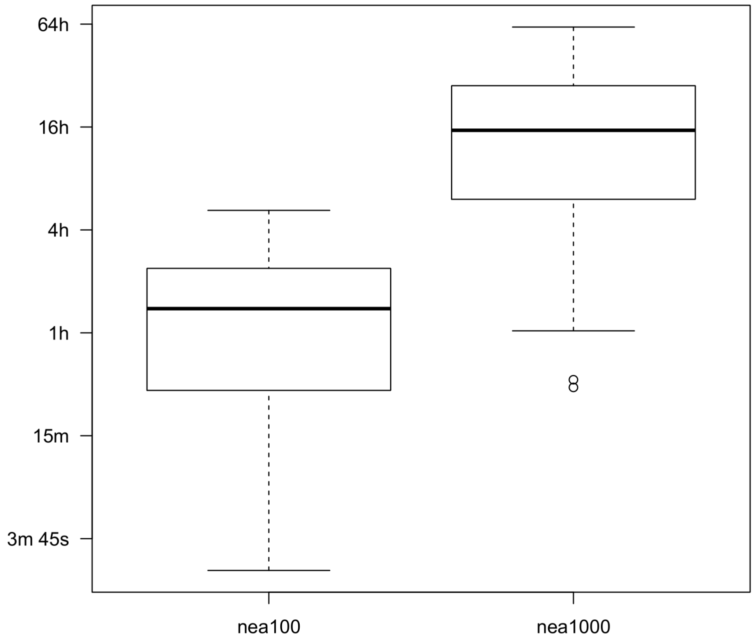

Figure S2: Distribution of the elapsed runtime of NEA for the 27 datasets of the GEO2KEGG benchmark set using 100 and 1000 permutations, respectively.

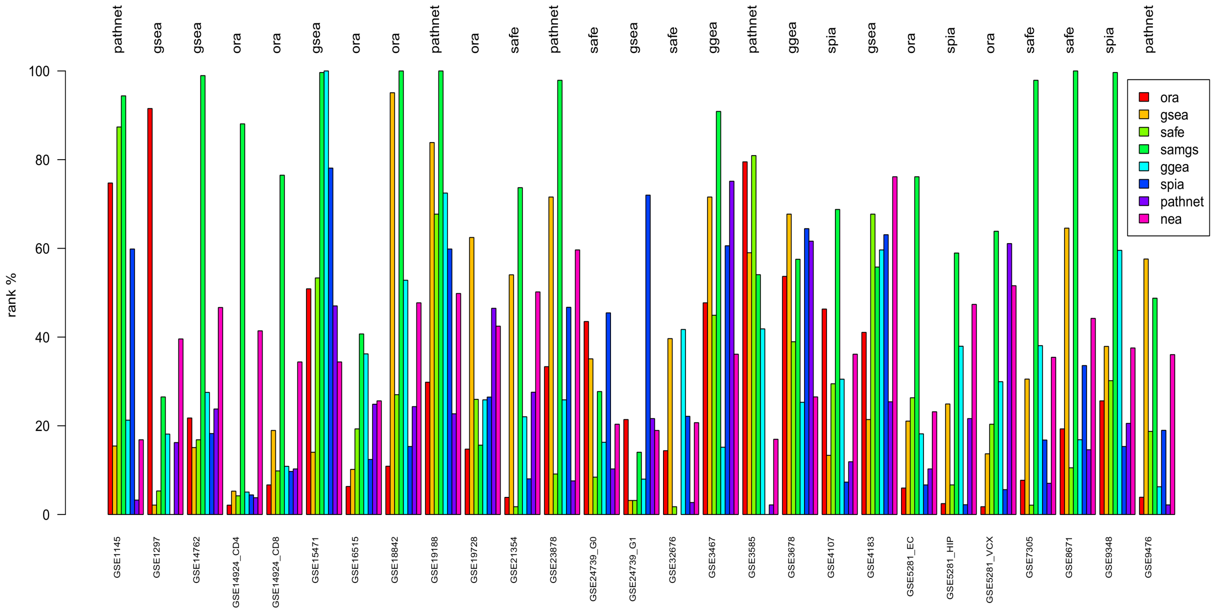

Figure S3: Ranks (based on gene set p-value) for the target KEGG pathways assigned to each of the 27 GEO datasets. The method with best rank is indicated on the top. Rankings of SPIA for GSE1297 and GSE3585 were not considered as they did not include the corresponding target pathway. Similarly, the ranking of SAMGS for GSE32678 was excluded from analysis as it returned an arithmetic error for all pathways under investigation.

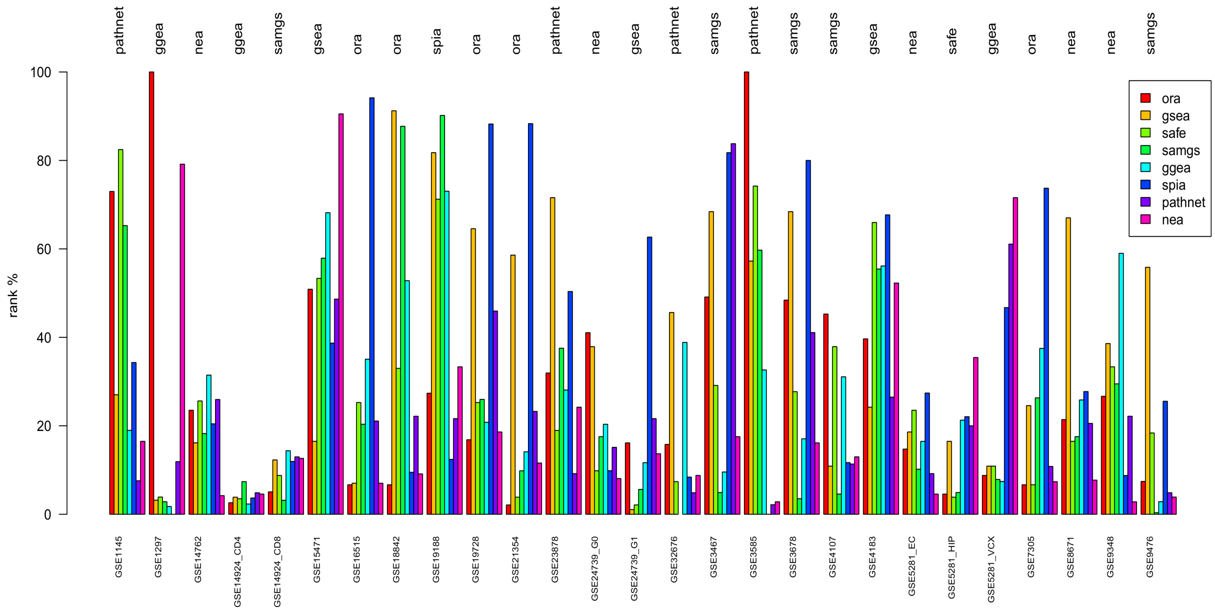

Figure S4: Ranks (based on gene set score) as for Figure S3.

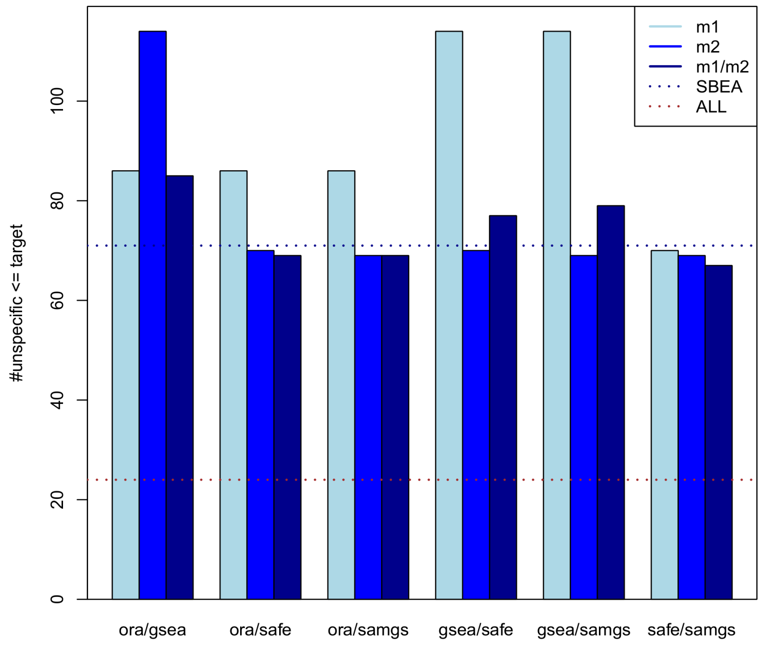

Figure S5: Shown is the total number of unspecific pathways ranked at least as good as the target pathway (y-axis) for the 4 set-based methods and each pairwise combination. Unspecific pathways are defined in the main text and listed in Table S1. The SBEA-combination of the 4 set-based methods and the ALL-combination of all 8 methods (4 set- and 4 network-based) are indicated with the blue and the brown dotted line, respectively.

Table S1: Unspecific pathways used for evaluation of method combination on the GEO2KEGG benchmark set. These pathways were selected as they did not share any genes with the target pathways, and the pathway titles suggested no relevance for the diseases studied in the GEO2KEGG benchmark set.

ID	Title
hsa04130	SNARE interactions in vesicular transport
hsa04140	Regulation of autophagy
hsa04142	Lysosome
hsa04146	Peroxisome
hsa04610	Complement and coagulation cascades
hsa04721	Synaptic vesicle cycle
hsa04950	Maturity onset diabetes of the young
hsa04964	Proximal tubule bicarbonate reclamation
hsa04966	Collecting duct acid secretion
hsa04975	Fat digestion and absorption
hsa04977	Vitamin digestion and absorption
hsa05150	Staphylococcus aureus infection
