# Supplementary material for: Bioconductor’s EnrichmentBrowser: seamless navigation through combined results of set- & network-based enrichment analysis
Source: BMC Bioinformatics. 2016 Jan 20;17:45. doi: 10.1186/s12859-016-0884-1 (PMC4721010; doi:10.1186/s12859-016-0884-1)
Supplement: Supplementary file 2 — EnrichmentBrowser output (ALL microarray data). Unzip and open the contained index.html in the browser to view the contents of this file (tested with Firefox 39.0). (ZIP 2775 kb) [file 12859_2016_884_MOESM2_ESM.zip › comb.html]

COMB - Table of Results


## COMB - Table of Results

| GENE.SET | TITLE | NR.GENES | ORA.RANK | GGEA.RANK | SUM.RANK | SET.VIEW | PATH.VIEW | GRAPH.VIEW |
| --- | --- | --- | --- | --- | --- | --- | --- | --- |
| GENE.SET | TITLE | NR.GENES | ORA.RANK | GGEA.RANK | SUM.RANK | SET.VIEW | PATH.VIEW | GRAPH.VIEW |
| hsa05416 | Viral myocarditis | 55 | 1 | 1 | 2 |  |  |  |
| hsa04520 | Adherens junction | 68 | 4 | 2 | 6 |  |  |  |
| hsa05217 | Basal cell carcinoma | 35 | 9 | 3 | 12 |  |  |  |
| hsa04622 | RIG-I-like receptor signaling pathway | 54 | 2 | 12 | 14 |  |  |  |
| hsa04210 | Apoptosis | 78 | 6 | 10 | 16 |  |  |  |
| hsa05202 | Transcriptional misregulation in cancer | 153 | 7 | 13 | 20 |  |  |  |

| GENE.SET | TITLE | NR.GENES | ORA.RANK | GGEA.RANK | SUM.RANK | SET.VIEW | PATH.VIEW | GRAPH.VIEW |
| --- | --- | --- | --- | --- | --- | --- | --- | --- |

(Page generated on Tue Aug 25 20:46:21 2015 by ReportingTools 2.9.1 and hwriter 1.3.2)
