# Supplementary material for: Bioconductor’s EnrichmentBrowser: seamless navigation through combined results of set- & network-based enrichment analysis
Source: BMC Bioinformatics. 2016 Jan 20;17:45. doi: 10.1186/s12859-016-0884-1 (PMC4721010; doi:10.1186/s12859-016-0884-1)
Supplement: Supplementary file 2 — EnrichmentBrowser output (ALL microarray data). Unzip and open the contained index.html in the browser to view the contents of this file (tested with Firefox 39.0). (ZIP 2775 kb) [file 12859_2016_884_MOESM2_ESM.zip › ggea.html]

GGEA - Table of Results


## GGEA - Table of Results

| GENE.SET | TITLE | NR.GENES | NORM.SCORE | P.VALUE | SET.VIEW | PATH.VIEW | GRAPH.VIEW |
| --- | --- | --- | --- | --- | --- | --- | --- |
| GENE.SET | TITLE | NR.GENES | NORM.SCORE | P.VALUE | SET.VIEW | PATH.VIEW | GRAPH.VIEW |
| hsa05416 | Viral myocarditis | 55 | 0.532 | 0.0006 |  |  |  |
| hsa04520 | Adherens junction | 68 | 0.523 | 0.0245 |  |  |  |
| hsa05217 | Basal cell carcinoma | 35 | 0.500 | 0.0187 |  |  |  |
| hsa04350 | TGF-beta signaling pathway | 71 | 0.488 | 0.0205 |  |  |  |

| GENE.SET | TITLE | NR.GENES | NORM.SCORE | P.VALUE | SET.VIEW | PATH.VIEW | GRAPH.VIEW |
| --- | --- | --- | --- | --- | --- | --- | --- |

(Page generated on Tue Aug 25 17:43:39 2015 by ReportingTools and hwriter )
