# Supplementary material for: Bioconductor’s EnrichmentBrowser: seamless navigation through combined results of set- & network-based enrichment analysis
Source: BMC Bioinformatics. 2016 Jan 20;17:45. doi: 10.1186/s12859-016-0884-1 (PMC4721010; doi:10.1186/s12859-016-0884-1)
Supplement: Supplementary file 2 — EnrichmentBrowser output (ALL microarray data). Unzip and open the contained index.html in the browser to view the contents of this file (tested with Firefox 39.0). (ZIP 2775 kb) [file 12859_2016_884_MOESM2_ESM.zip › hsa04210.html]

hsa04210: Gene Report


## hsa04210: Gene Report

| ENTREZID | SYMBOL | GENENAME | FC | ADJ.PVAL |
| --- | --- | --- | --- | --- |
| ENTREZID | SYMBOL | GENENAME | FC | ADJ.PVAL |
| 10000 | AKT3 | v-akt murine thymoma viral oncogene homolog 3 | 0.04 | 0.8600 |
| 11213 | IRAK3 | interleukin-1 receptor-associated kinase 3 | 0.65 | 0.2100 |
| 1147 | CHUK | conserved helix-loop-helix ubiquitous kinase | 0.03 | 0.9700 |
| 1439 | CSF2RB | colony stimulating factor 2 receptor, beta, low-affinity (granulocyte-macrophage) | -0.02 | 0.9600 |
| 1676 | DFFA | DNA fragmentation factor, 45kDa, alpha polypeptide | -0.18 | 0.3500 |
| 1677 | DFFB | DNA fragmentation factor, 40kDa, beta polypeptide (caspase-activated DNase) | -0.07 | 0.8600 |
| 2021 | ENDOG | endonuclease G | -0.05 | 0.8600 |
| 207 | AKT1 | v-akt murine thymoma viral oncogene homolog 1 | -0.17 | 0.7800 |
| 208 | AKT2 | v-akt murine thymoma viral oncogene homolog 2 | -0.01 | 0.9600 |
| 317 | APAF1 | apoptotic peptidase activating factor 1 | -0.01 | 0.9900 |
| 329 | BIRC2 | baculoviral IAP repeat containing 2 | 0.20 | 0.6400 |
| 330 | BIRC3 | baculoviral IAP repeat containing 3 | 0.39 | 0.2800 |
| 331 | XIAP | X-linked inhibitor of apoptosis, E3 ubiquitin protein ligase | 0.01 | 0.9900 |
| 355 | FAS | Fas cell surface death receptor | 0.06 | 0.6600 |
| 3551 | IKBKB | inhibitor of kappa light polypeptide gene enhancer in B-cells, kinase beta | -0.10 | 0.6700 |
| 3552 | IL1A | interleukin 1, alpha | 0.11 | 0.7900 |
| 3553 | IL1B | interleukin 1, beta | -0.02 | 0.9900 |
| 3554 | IL1R1 | interleukin 1 receptor, type I | 0.10 | 0.8300 |
| 3556 | IL1RAP | interleukin 1 receptor accessory protein | 0.75 | 0.0084 |
| 356 | FASLG | Fas ligand (TNF superfamily, member 6) | 0.00 | 0.9900 |
| 3562 | IL3 | interleukin 3 | 0.01 | 0.9900 |
| 3563 | IL3RA | interleukin 3 receptor, alpha (low affinity) | -0.22 | 0.5600 |
| 3654 | IRAK1 | interleukin-1 receptor-associated kinase 1 | -0.29 | 0.4200 |
| 4615 | MYD88 | myeloid differentiation primary response 88 | -0.11 | 0.7800 |
| 472 | ATM | ATM serine/threonine kinase | -0.01 | 0.9900 |
| 4790 | NFKB1 | nuclear factor of kappa light polypeptide gene enhancer in B-cells 1 | 0.35 | 0.2300 |
| 4792 | NFKBIA | nuclear factor of kappa light polypeptide gene enhancer in B-cells inhibitor, alpha | 0.71 | 0.0410 |
| 4803 | NGF | nerve growth factor (beta polypeptide) | -0.06 | 0.7800 |
| 4914 | NTRK1 | neurotrophic tyrosine kinase, receptor, type 1 | -0.12 | 0.5800 |
| 5290 | PIK3CA | phosphatidylinositol-4,5-bisphosphate 3-kinase, catalytic subunit alpha | 0.28 | 0.6200 |
| 5291 | PIK3CB | phosphatidylinositol-4,5-bisphosphate 3-kinase, catalytic subunit beta | -0.05 | 0.6600 |
| 5293 | PIK3CD | phosphatidylinositol-4,5-bisphosphate 3-kinase, catalytic subunit delta | -0.01 | 0.9900 |
| 5294 | PIK3CG | phosphatidylinositol-4,5-bisphosphate 3-kinase, catalytic subunit gamma | -0.01 | 1.0000 |
| 5295 | PIK3R1 | phosphoinositide-3-kinase, regulatory subunit 1 (alpha) | -0.23 | 0.6300 |
| 5296 | PIK3R2 | phosphoinositide-3-kinase, regulatory subunit 2 (beta) | 0.09 | 0.8400 |
| 54205 | CYCS | cytochrome c, somatic | -0.22 | 0.7200 |
| 5530 | PPP3CA | protein phosphatase 3, catalytic subunit, alpha isozyme | -0.08 | 0.8500 |
| 5532 | PPP3CB | protein phosphatase 3, catalytic subunit, beta isozyme | -0.01 | 0.9900 |
| 5533 | PPP3CC | protein phosphatase 3, catalytic subunit, gamma isozyme | 0.16 | 0.3500 |
| 5534 | PPP3R1 | protein phosphatase 3, regulatory subunit B, alpha | 0.04 | 0.8200 |
| 5566 | PRKACA | protein kinase, cAMP-dependent, catalytic, alpha | -0.01 | 0.9800 |
| 5567 | PRKACB | protein kinase, cAMP-dependent, catalytic, beta | 0.15 | 0.8400 |
| 5568 | PRKACG | protein kinase, cAMP-dependent, catalytic, gamma | 0.03 | 0.9300 |
| 5573 | PRKAR1A | protein kinase, cAMP-dependent, regulatory, type I, alpha | -0.05 | 0.9700 |
| 5575 | PRKAR1B | protein kinase, cAMP-dependent, regulatory, type I, beta | -0.03 | 0.9000 |
| 5576 | PRKAR2A | protein kinase, cAMP-dependent, regulatory, type II, alpha | -0.07 | 0.7900 |
| 5577 | PRKAR2B | protein kinase, cAMP-dependent, regulatory, type II, beta | 0.20 | 0.6600 |
| 5613 | PRKX | protein kinase, X-linked | -0.46 | 0.2500 |
| 572 | BAD | BCL2-associated agonist of cell death | -0.13 | 0.7400 |
| 581 | BAX | BCL2-associated X protein | -0.03 | 0.9600 |
| 596 | BCL2 | B-cell CLL/lymphoma 2 | -0.13 | 0.2100 |
| 5970 | RELA | v-rel avian reticuloendotheliosis viral oncogene homolog A | -0.04 | 0.9500 |
| 598 | BCL2L1 | BCL2-like 1 | -0.03 | 0.9700 |
| 637 | BID | BH3 interacting domain death agonist | 0.03 | 0.9400 |
| 7124 | TNF | tumor necrosis factor | 0.12 | 0.7100 |
| 7132 | TNFRSF1A | tumor necrosis factor receptor superfamily, member 1A | 0.11 | 0.7800 |
| 7157 | TP53 | tumor protein p53 | -0.10 | 0.7400 |
| 7186 | TRAF2 | TNF receptor-associated factor 2 | 0.00 | 0.9900 |
| 823 | CAPN1 | calpain 1, (mu/I) large subunit | 0.07 | 0.9100 |
| 824 | CAPN2 | calpain 2, (m/II) large subunit | 0.60 | 0.0630 |
| 836 | CASP3 | caspase 3, apoptosis-related cysteine peptidase | 0.20 | 0.4900 |
| 839 | CASP6 | caspase 6, apoptosis-related cysteine peptidase | -0.05 | 0.9600 |
| 840 | CASP7 | caspase 7, apoptosis-related cysteine peptidase | 0.17 | 0.6800 |
| 841 | CASP8 | caspase 8, apoptosis-related cysteine peptidase | 0.48 | 0.0016 |
| 842 | CASP9 | caspase 9, apoptosis-related cysteine peptidase | -0.08 | 0.8100 |
| 843 | CASP10 | caspase 10, apoptosis-related cysteine peptidase | 0.32 | 0.0038 |
| 8503 | PIK3R3 | phosphoinositide-3-kinase, regulatory subunit 3 (gamma) | -0.04 | 0.8300 |
| 8517 | IKBKG | inhibitor of kappa light polypeptide gene enhancer in B-cells, kinase gamma | -0.02 | 0.9600 |
| 8717 | TRADD | TNFRSF1A-associated via death domain | 0.03 | 0.9400 |
| 8737 | RIPK1 | receptor (TNFRSF)-interacting serine-threonine kinase 1 | 0.09 | 0.8200 |
| 8743 | TNFSF10 | tumor necrosis factor (ligand) superfamily, member 10 | 0.04 | 0.9600 |
| 8772 | FADD | Fas (TNFRSF6)-associated via death domain | 0.02 | 0.9500 |
| 8793 | TNFRSF10D | tumor necrosis factor receptor superfamily, member 10d, decoy with truncated death domain | -0.01 | 0.9600 |
| 8794 | TNFRSF10C | tumor necrosis factor receptor superfamily, member 10c, decoy without an intracellular domain | 0.00 | 1.0000 |
| 8795 | TNFRSF10B | tumor necrosis factor receptor superfamily, member 10b | 0.13 | 0.8300 |
| 8837 | CFLAR | CASP8 and FADD-like apoptosis regulator | 0.11 | 0.5500 |
| 9020 | MAP3K14 | mitogen-activated protein kinase kinase kinase 14 | 0.00 | 0.9900 |
| 9131 | AIFM1 | apoptosis-inducing factor, mitochondrion-associated, 1 | -0.16 | 0.6800 |

| ENTREZID | SYMBOL | GENENAME | FC | ADJ.PVAL |
| --- | --- | --- | --- | --- |

(Page generated on Tue Aug 25 20:46:20 2015 by ReportingTools 2.9.1 and hwriter 1.3.2)
