# Supplementary material for: Bioconductor’s EnrichmentBrowser: seamless navigation through combined results of set- & network-based enrichment analysis
Source: BMC Bioinformatics. 2016 Jan 20;17:45. doi: 10.1186/s12859-016-0884-1 (PMC4721010; doi:10.1186/s12859-016-0884-1)
Supplement: Supplementary file 2 — EnrichmentBrowser output (ALL microarray data). Unzip and open the contained index.html in the browser to view the contents of this file (tested with Firefox 39.0). (ZIP 2775 kb) [file 12859_2016_884_MOESM2_ESM.zip › hsa04350.html]

hsa04350: Gene Report


## hsa04350: Gene Report

| ENTREZID | SYMBOL | GENENAME | FC | ADJ.PVAL |
| --- | --- | --- | --- | --- |
| ENTREZID | SYMBOL | GENENAME | FC | ADJ.PVAL |
| 1030 | CDKN2B | cyclin-dependent kinase inhibitor 2B (p15, inhibits CDK4) | -0.09 | 0.7000 |
| 10468 | FST | follistatin | -0.04 | 0.9200 |
| 10637 | LEFTY1 | left-right determination factor 1 | 0.00 | 0.9900 |
| 1387 | CREBBP | CREB binding protein | 0.00 | 1.0000 |
| 1874 | E2F4 | E2F transcription factor 4, p107/p130-binding | -0.05 | 0.8600 |
| 1875 | E2F5 | E2F transcription factor 5, p130-binding | -0.21 | 0.5800 |
| 2033 | EP300 | E1A binding protein p300 | -0.02 | 0.9800 |
| 25805 | BAMBI | BMP and activin membrane-bound inhibitor | -0.14 | 0.4800 |
| 268 | AMH | anti-Mullerian hormone | -0.21 | 0.1500 |
| 269 | AMHR2 | anti-Mullerian hormone receptor, type II | -0.03 | 0.9200 |
| 3397 | ID1 | inhibitor of DNA binding 1, dominant negative helix-loop-helix protein | 0.57 | 0.0180 |
| 3398 | ID2 | inhibitor of DNA binding 2, dominant negative helix-loop-helix protein | 0.14 | 0.8300 |
| 3399 | ID3 | inhibitor of DNA binding 3, dominant negative helix-loop-helix protein | 1.10 | 0.0150 |
| 3400 | ID4 | inhibitor of DNA binding 4, dominant negative helix-loop-helix protein | -0.04 | 0.9500 |
| 3458 | IFNG | interferon, gamma | 0.26 | 0.1300 |
| 353500 | BMP8A | bone morphogenetic protein 8a | -0.10 | 0.7800 |
| 3624 | INHBA | inhibin, beta A | 0.02 | 0.9400 |
| 3625 | INHBB | inhibin, beta B | 0.19 | 0.8100 |
| 3626 | INHBC | inhibin, beta C | 0.03 | 0.9200 |
| 387 | RHOA | ras homolog family member A | 0.27 | 0.1200 |
| 4052 | LTBP1 | latent transforming growth factor beta binding protein 1 | -0.06 | 0.7400 |
| 4086 | SMAD1 | SMAD family member 1 | -0.02 | 1.0000 |
| 4087 | SMAD2 | SMAD family member 2 | -0.26 | 0.4100 |
| 4088 | SMAD3 | SMAD family member 3 | 0.13 | 0.8500 |
| 4089 | SMAD4 | SMAD family member 4 | -0.05 | 0.9600 |
| 4090 | SMAD5 | SMAD family member 5 | 0.03 | 0.9600 |
| 4091 | SMAD6 | SMAD family member 6 | 0.01 | 0.9700 |
| 4092 | SMAD7 | SMAD family member 7 | 0.31 | 0.7400 |
| 4093 | SMAD9 | SMAD family member 9 | 0.03 | 0.9100 |
| 4609 | MYC | v-myc avian myelocytomatosis viral oncogene homolog | -0.16 | 0.6200 |
| 5308 | PITX2 | paired-like homeodomain 2 | 0.04 | 0.7900 |
| 5515 | PPP2CA | protein phosphatase 2, catalytic subunit, alpha isozyme | 0.10 | 0.6000 |
| 5516 | PPP2CB | protein phosphatase 2, catalytic subunit, beta isozyme | 0.11 | 0.8500 |
| 5518 | PPP2R1A | protein phosphatase 2, regulatory subunit A, alpha | -0.13 | 0.8800 |
| 5519 | PPP2R1B | protein phosphatase 2, regulatory subunit A, beta | -0.02 | 0.9400 |
| 5594 | MAPK1 | mitogen-activated protein kinase 1 | 0.00 | 1.0000 |
| 5595 | MAPK3 | mitogen-activated protein kinase 3 | 0.04 | 0.9000 |
| 57154 | SMURF1 | SMAD specific E3 ubiquitin protein ligase 1 | -0.10 | 0.6100 |
| 5933 | RBL1 | retinoblastoma-like 1 | -0.01 | 0.9900 |
| 6093 | ROCK1 | Rho-associated, coiled-coil containing protein kinase 1 | 0.00 | 1.0000 |
| 6198 | RPS6KB1 | ribosomal protein S6 kinase, 70kDa, polypeptide 1 | -0.01 | 0.9900 |
| 6199 | RPS6KB2 | ribosomal protein S6 kinase, 70kDa, polypeptide 2 | -0.03 | 0.9400 |
| 64750 | SMURF2 | SMAD specific E3 ubiquitin protein ligase 2 | -0.01 | 1.0000 |
| 650 | BMP2 | bone morphogenetic protein 2 | 0.43 | 0.6300 |
| 6500 | SKP1 | S-phase kinase-associated protein 1 | -0.04 | 0.9500 |
| 652 | BMP4 | bone morphogenetic protein 4 | 0.02 | 0.9500 |
| 653 | BMP5 | bone morphogenetic protein 5 | 0.02 | 0.9300 |
| 654 | BMP6 | bone morphogenetic protein 6 | -0.08 | 0.4300 |
| 655 | BMP7 | bone morphogenetic protein 7 | -0.04 | 0.9000 |
| 657 | BMPR1A | bone morphogenetic protein receptor, type IA | 0.01 | 0.9900 |
| 658 | BMPR1B | bone morphogenetic protein receptor, type IB | 0.01 | 0.9900 |
| 659 | BMPR2 | bone morphogenetic protein receptor, type II (serine/threonine kinase) | 0.02 | 0.9600 |
| 6667 | SP1 | Sp1 transcription factor | 0.01 | 0.9900 |
| 7027 | TFDP1 | transcription factor Dp-1 | 0.14 | 0.7900 |
| 7040 | TGFB1 | transforming growth factor, beta 1 | -0.01 | 0.9900 |
| 7042 | TGFB2 | transforming growth factor, beta 2 | -0.03 | 0.9000 |
| 7043 | TGFB3 | transforming growth factor, beta 3 | 0.01 | 0.9800 |
| 7044 | LEFTY2 | left-right determination factor 2 | 0.01 | 0.9700 |
| 7046 | TGFBR1 | transforming growth factor, beta receptor 1 | 0.00 | 0.9900 |
| 7048 | TGFBR2 | transforming growth factor, beta receptor II (70/80kDa) | 0.04 | 0.9700 |
| 7057 | THBS1 | thrombospondin 1 | 0.08 | 0.5700 |
| 7124 | TNF | tumor necrosis factor | 0.12 | 0.7100 |
| 8200 | GDF5 | growth differentiation factor 5 | -0.01 | 0.9700 |
| 8454 | CUL1 | cullin 1 | 0.02 | 0.9800 |
| 8646 | CHRD | chordin | -0.02 | 0.9600 |
| 90 | ACVR1 | activin A receptor, type I | 0.16 | 0.5700 |
| 91 | ACVR1B | activin A receptor, type IB | -0.01 | 0.9900 |
| 92 | ACVR2A | activin A receptor, type IIA | 0.74 | 0.0012 |
| 93 | ACVR2B | activin A receptor, type IIB | -0.08 | 0.8200 |
| 9372 | ZFYVE9 | zinc finger, FYVE domain containing 9 | 0.01 | 0.9500 |
| 9765 | ZFYVE16 | zinc finger, FYVE domain containing 16 | 0.19 | 0.4700 |

| ENTREZID | SYMBOL | GENENAME | FC | ADJ.PVAL |
| --- | --- | --- | --- | --- |

(Page generated on Tue Aug 25 20:50:56 2015 by ReportingTools 2.9.1 and hwriter 1.3.2)
