# Supplementary material for: Bioconductor’s EnrichmentBrowser: seamless navigation through combined results of set- & network-based enrichment analysis
Source: BMC Bioinformatics. 2016 Jan 20;17:45. doi: 10.1186/s12859-016-0884-1 (PMC4721010; doi:10.1186/s12859-016-0884-1)
Supplement: Supplementary file 2 — EnrichmentBrowser output (ALL microarray data). Unzip and open the contained index.html in the browser to view the contents of this file (tested with Firefox 39.0). (ZIP 2775 kb) [file 12859_2016_884_MOESM2_ESM.zip › hsa04520.html]

hsa04520: Gene Report


## hsa04520: Gene Report

| ENTREZID | SYMBOL | GENENAME | FC | ADJ.PVAL |
| --- | --- | --- | --- | --- |
| ENTREZID | SYMBOL | GENENAME | FC | ADJ.PVAL |
| 10163 | WASF2 | WAS protein family, member 2 | -0.08 | 7.4e-01 |
| 10458 | BAIAP2 | BAI1-associated protein 2 | -0.01 | 9.8e-01 |
| 10580 | SORBS1 | sorbin and SH3 domain containing 1 | -0.03 | 9.4e-01 |
| 10810 | WASF3 | WAS protein family, member 3 | 0.00 | 9.9e-01 |
| 117178 | SSX2IP | synovial sarcoma, X breakpoint 2 interacting protein | -0.01 | 9.5e-01 |
| 1387 | CREBBP | CREB binding protein | 0.00 | 1.0e+00 |
| 1457 | CSNK2A1 | casein kinase 2, alpha 1 polypeptide | -0.04 | 8.2e-01 |
| 1459 | CSNK2A2 | casein kinase 2, alpha prime polypeptide | 0.03 | 9.2e-01 |
| 1460 | CSNK2B | casein kinase 2, beta polypeptide | -0.16 | 7.5e-01 |
| 1495 | CTNNA1 | catenin (cadherin-associated protein), alpha 1, 102kDa | 0.03 | 9.8e-01 |
| 1496 | CTNNA2 | catenin (cadherin-associated protein), alpha 2 | -0.01 | 9.7e-01 |
| 1499 | CTNNB1 | catenin (cadherin-associated protein), beta 1, 88kDa | 0.26 | 5.5e-01 |
| 1500 | CTNND1 | catenin (cadherin-associated protein), delta 1 | 0.13 | 3.1e-01 |
| 1956 | EGFR | epidermal growth factor receptor | 0.00 | 9.9e-01 |
| 2033 | EP300 | E1A binding protein p300 | -0.02 | 9.8e-01 |
| 2064 | ERBB2 | erb-b2 receptor tyrosine kinase 2 | 0.08 | 7.3e-01 |
| 2241 | FER | fer (fps/fes related) tyrosine kinase | 0.01 | 9.7e-01 |
| 2260 | FGFR1 | fibroblast growth factor receptor 1 | 0.29 | 2.1e-01 |
| 2534 | FYN | FYN proto-oncogene, Src family tyrosine kinase | 0.47 | 1.4e-03 |
| 25945 | PVRL3 | poliovirus receptor-related 3 | -0.06 | 7.3e-01 |
| 3480 | IGF1R | insulin-like growth factor 1 receptor | -0.09 | 5.7e-01 |
| 3643 | INSR | insulin receptor | -0.11 | 7.3e-01 |
| 387 | RHOA | ras homolog family member A | 0.27 | 1.2e-01 |
| 4008 | LMO7 | LIM domain 7 | 0.04 | 8.7e-01 |
| 4087 | SMAD2 | SMAD family member 2 | -0.26 | 4.1e-01 |
| 4088 | SMAD3 | SMAD family member 3 | 0.13 | 8.5e-01 |
| 4089 | SMAD4 | SMAD family member 4 | -0.05 | 9.6e-01 |
| 4233 | MET | MET proto-oncogene, receptor tyrosine kinase | 0.01 | 9.7e-01 |
| 4301 | MLLT4 | myeloid/lymphoid or mixed-lineage leukemia (trithorax homolog, Drosophila); translocated to, 4 | 0.05 | 7.4e-01 |
| 51176 | LEF1 | lymphoid enhancer-binding factor 1 | 0.93 | 2.8e-02 |
| 52 | ACP1 | acid phosphatase 1, soluble | -0.03 | 9.5e-01 |
| 5594 | MAPK1 | mitogen-activated protein kinase 1 | 0.00 | 1.0e+00 |
| 5595 | MAPK3 | mitogen-activated protein kinase 3 | 0.04 | 9.0e-01 |
| 56288 | PARD3 | par-3 family cell polarity regulator | 0.07 | 8.1e-01 |
| 5770 | PTPN1 | protein tyrosine phosphatase, non-receptor type 1 | 0.03 | 9.6e-01 |
| 5777 | PTPN6 | protein tyrosine phosphatase, non-receptor type 6 | -0.19 | 8.2e-01 |
| 5787 | PTPRB | protein tyrosine phosphatase, receptor type, B | 0.02 | 9.3e-01 |
| 5792 | PTPRF | protein tyrosine phosphatase, receptor type, F | -0.05 | 8.9e-01 |
| 5795 | PTPRJ | protein tyrosine phosphatase, receptor type, J | 0.04 | 7.4e-01 |
| 5797 | PTPRM | protein tyrosine phosphatase, receptor type, M | -0.29 | 8.2e-01 |
| 5818 | PVRL1 | poliovirus receptor-related 1 (herpesvirus entry mediator C) | -0.07 | 7.1e-01 |
| 5819 | PVRL2 | poliovirus receptor-related 2 (herpesvirus entry mediator B) | 0.27 | 5.1e-01 |
| 5879 | RAC1 | ras-related C3 botulinum toxin substrate 1 (rho family, small GTP binding protein Rac1) | -0.03 | 9.8e-01 |
| 5880 | RAC2 | ras-related C3 botulinum toxin substrate 2 (rho family, small GTP binding protein Rac2) | -0.09 | 9.3e-01 |
| 5881 | RAC3 | ras-related C3 botulinum toxin substrate 3 (rho family, small GTP binding protein Rac3) | -0.03 | 9.3e-01 |
| 60 | ACTB | actin, beta | 0.01 | 1.0e+00 |
| 6591 | SNAI2 | snail family zinc finger 2 | 0.01 | 9.9e-01 |
| 6714 | SRC | SRC proto-oncogene, non-receptor tyrosine kinase | -0.03 | 9.2e-01 |
| 6885 | MAP3K7 | mitogen-activated protein kinase kinase kinase 7 | 0.05 | 9.5e-01 |
| 6932 | TCF7 | transcription factor 7 (T-cell specific, HMG-box) | 0.41 | 1.2e-01 |
| 6934 | TCF7L2 | transcription factor 7-like 2 (T-cell specific, HMG-box) | 0.18 | 6.2e-01 |
| 7046 | TGFBR1 | transforming growth factor, beta receptor 1 | 0.00 | 9.9e-01 |
| 7048 | TGFBR2 | transforming growth factor, beta receptor II (70/80kDa) | 0.04 | 9.7e-01 |
| 7082 | TJP1 | tight junction protein 1 | 0.03 | 9.4e-01 |
| 71 | ACTG1 | actin gamma 1 | -0.03 | 9.6e-01 |
| 7414 | VCL | vinculin | 0.32 | 5.2e-01 |
| 7454 | WAS | Wiskott-Aldrich syndrome | -0.06 | 7.8e-01 |
| 7525 | YES1 | YES proto-oncogene 1, Src family tyrosine kinase | 1.43 | 5.9e-06 |
| 81 | ACTN4 | actinin, alpha 4 | 0.23 | 2.0e-01 |
| 87 | ACTN1 | actinin, alpha 1 | 0.88 | 1.6e-03 |
| 88 | ACTN2 | actinin, alpha 2 | 0.02 | 9.1e-01 |
| 8826 | IQGAP1 | IQ motif containing GTPase activating protein 1 | 0.22 | 6.0e-01 |
| 89 | ACTN3 | actinin, alpha 3 (gene/pseudogene) | -0.06 | 8.0e-01 |
| 8936 | WASF1 | WAS protein family, member 1 | -0.14 | 8.9e-01 |
| 8976 | WASL | Wiskott-Aldrich syndrome-like | -0.02 | 9.6e-01 |
| 9855 | FARP2 | FERM, RhoGEF and pleckstrin domain protein 2 | -0.05 | 7.9e-01 |
| 998 | CDC42 | cell division cycle 42 | 0.09 | 9.3e-01 |
| 999 | CDH1 | cadherin 1, type 1, E-cadherin (epithelial) | -0.03 | 8.3e-01 |

| ENTREZID | SYMBOL | GENENAME | FC | ADJ.PVAL |
| --- | --- | --- | --- | --- |

(Page generated on Tue Aug 25 20:50:56 2015 by ReportingTools 2.9.1 and hwriter 1.3.2)
