# Supplementary material for: Bioconductor’s EnrichmentBrowser: seamless navigation through combined results of set- & network-based enrichment analysis
Source: BMC Bioinformatics. 2016 Jan 20;17:45. doi: 10.1186/s12859-016-0884-1 (PMC4721010; doi:10.1186/s12859-016-0884-1)
Supplement: Supplementary file 2 — EnrichmentBrowser output (ALL microarray data). Unzip and open the contained index.html in the browser to view the contents of this file (tested with Firefox 39.0). (ZIP 2775 kb) [file 12859_2016_884_MOESM2_ESM.zip › hsa04622.html]

hsa04622: Gene Report


## hsa04622: Gene Report

| ENTREZID | SYMBOL | GENENAME | FC | ADJ.PVAL |
| --- | --- | --- | --- | --- |
| ENTREZID | SYMBOL | GENENAME | FC | ADJ.PVAL |
| 10010 | TANK | TRAF family member-associated NFKB activator | 0.23 | 0.2300 |
| 1147 | CHUK | conserved helix-loop-helix ubiquitous kinase | 0.03 | 0.9700 |
| 1432 | MAPK14 | mitogen-activated protein kinase 14 | -0.02 | 0.9600 |
| 1540 | CYLD | cylindromatosis (turban tumor syndrome) | 0.21 | 0.6800 |
| 1654 | DDX3X | DEAD (Asp-Glu-Ala-Asp) box helicase 3, X-linked | 0.12 | 0.8700 |
| 3439 | IFNA1 | interferon, alpha 1 | 0.02 | 0.9600 |
| 3440 | IFNA2 | interferon, alpha 2 | -0.01 | 0.9600 |
| 3441 | IFNA4 | interferon, alpha 4 | -0.02 | 0.9500 |
| 3442 | IFNA5 | interferon, alpha 5 | 0.00 | 1.0000 |
| 3445 | IFNA8 | interferon, alpha 8 | 0.02 | 0.9500 |
| 3446 | IFNA10 | interferon, alpha 10 | -0.01 | 0.9800 |
| 3448 | IFNA14 | interferon, alpha 14 | 0.03 | 0.9300 |
| 3449 | IFNA16 | interferon, alpha 16 | -0.01 | 0.9600 |
| 3452 | IFNA21 | interferon, alpha 21 | 0.06 | 0.8100 |
| 3456 | IFNB1 | interferon, beta 1, fibroblast | -0.12 | 0.3800 |
| 3467 | IFNW1 | interferon, omega 1 | 0.02 | 0.9500 |
| 3551 | IKBKB | inhibitor of kappa light polypeptide gene enhancer in B-cells, kinase beta | -0.10 | 0.6700 |
| 3576 | CXCL8 | chemokine (C-X-C motif) ligand 8 | 0.50 | 0.6200 |
| 3592 | IL12A | interleukin 12A | 0.21 | 0.1900 |
| 3593 | IL12B | interleukin 12B | 0.06 | 0.6300 |
| 3627 | CXCL10 | chemokine (C-X-C motif) ligand 10 | -0.04 | 0.8200 |
| 3661 | IRF3 | interferon regulatory factor 3 | -0.13 | 0.7000 |
| 3665 | IRF7 | interferon regulatory factor 7 | -0.60 | 0.0600 |
| 4214 | MAP3K1 | mitogen-activated protein kinase kinase kinase 1, E3 ubiquitin protein ligase | -0.12 | 0.8500 |
| 4790 | NFKB1 | nuclear factor of kappa light polypeptide gene enhancer in B-cells 1 | 0.35 | 0.2300 |
| 4792 | NFKBIA | nuclear factor of kappa light polypeptide gene enhancer in B-cells inhibitor, alpha | 0.71 | 0.0410 |
| 4793 | NFKBIB | nuclear factor of kappa light polypeptide gene enhancer in B-cells inhibitor, beta | -0.08 | 0.7500 |
| 5300 | PIN1 | peptidylprolyl cis/trans isomerase, NIMA-interacting 1 | -0.13 | 0.7500 |
| 5599 | MAPK8 | mitogen-activated protein kinase 8 | -0.03 | 0.8500 |
| 5600 | MAPK11 | mitogen-activated protein kinase 11 | -0.07 | 0.6400 |
| 5601 | MAPK9 | mitogen-activated protein kinase 9 | -0.01 | 0.9900 |
| 5602 | MAPK10 | mitogen-activated protein kinase 10 | 0.03 | 0.8400 |
| 5603 | MAPK13 | mitogen-activated protein kinase 13 | 0.01 | 0.9900 |
| 5970 | RELA | v-rel avian reticuloendotheliosis viral oncogene homolog A | -0.04 | 0.9500 |
| 6300 | MAPK12 | mitogen-activated protein kinase 12 | -0.03 | 0.9200 |
| 64135 | IFIH1 | interferon induced with helicase C domain 1 | -0.01 | 0.9800 |
| 6885 | MAP3K7 | mitogen-activated protein kinase kinase kinase 7 | 0.05 | 0.9500 |
| 7124 | TNF | tumor necrosis factor | 0.12 | 0.7100 |
| 7186 | TRAF2 | TNF receptor-associated factor 2 | 0.00 | 0.9900 |
| 7187 | TRAF3 | TNF receptor-associated factor 3 | -0.03 | 0.9300 |
| 7189 | TRAF6 | TNF receptor-associated factor 6, E3 ubiquitin protein ligase | 0.14 | 0.6900 |
| 7706 | TRIM25 | tripartite motif containing 25 | -0.02 | 0.9600 |
| 80143 | SIKE1 | suppressor of IKBKE 1 | 0.05 | 0.7900 |
| 841 | CASP8 | caspase 8, apoptosis-related cysteine peptidase | 0.48 | 0.0016 |
| 843 | CASP10 | caspase 10, apoptosis-related cysteine peptidase | 0.32 | 0.0038 |
| 8517 | IKBKG | inhibitor of kappa light polypeptide gene enhancer in B-cells, kinase gamma | -0.02 | 0.9600 |
| 8717 | TRADD | TNFRSF1A-associated via death domain | 0.03 | 0.9400 |
| 8737 | RIPK1 | receptor (TNFRSF)-interacting serine-threonine kinase 1 | 0.09 | 0.8200 |
| 8772 | FADD | Fas (TNFRSF6)-associated via death domain | 0.02 | 0.9500 |
| 9140 | ATG12 | autophagy related 12 | 0.05 | 0.8800 |
| 9474 | ATG5 | autophagy related 5 | 0.06 | 0.8000 |
| 9636 | ISG15 | ISG15 ubiquitin-like modifier | -0.59 | 0.0160 |
| 9641 | IKBKE | inhibitor of kappa light polypeptide gene enhancer in B-cells, kinase epsilon | 0.01 | 0.9700 |
| 9755 | TBKBP1 | TBK1 binding protein 1 | -0.06 | 0.8900 |

| ENTREZID | SYMBOL | GENENAME | FC | ADJ.PVAL |
| --- | --- | --- | --- | --- |

(Page generated on Tue Aug 25 20:48:53 2015 by ReportingTools 2.9.1 and hwriter 1.3.2)
