# Supplementary material for: Bioconductor’s EnrichmentBrowser: seamless navigation through combined results of set- & network-based enrichment analysis
Source: BMC Bioinformatics. 2016 Jan 20;17:45. doi: 10.1186/s12859-016-0884-1 (PMC4721010; doi:10.1186/s12859-016-0884-1)
Supplement: Supplementary file 2 — EnrichmentBrowser output (ALL microarray data). Unzip and open the contained index.html in the browser to view the contents of this file (tested with Firefox 39.0). (ZIP 2775 kb) [file 12859_2016_884_MOESM2_ESM.zip › hsa05130.html]

hsa05130: Gene Report


## hsa05130: Gene Report

| ENTREZID | SYMBOL | GENENAME | FC | ADJ.PVAL |
| --- | --- | --- | --- | --- |
| ENTREZID | SYMBOL | GENENAME | FC | ADJ.PVAL |
| 100506658 | OCLN | occludin | 0.03 | 9.6e-01 |
| 10092 | ARPC5 | actin related protein 2/3 complex, subunit 5, 16kDa | 0.14 | 9.0e-01 |
| 10093 | ARPC4 | actin related protein 2/3 complex, subunit 4, 20kDa | -0.07 | 9.2e-01 |
| 10094 | ARPC3 | actin related protein 2/3 complex, subunit 3, 21kDa | -0.04 | 9.6e-01 |
| 10095 | ARPC1B | actin related protein 2/3 complex, subunit 1B, 41kDa | 0.07 | 9.4e-01 |
| 10109 | ARPC2 | actin related protein 2/3 complex, subunit 2, 34kDa | 0.09 | 8.5e-01 |
| 10376 | TUBA1B | tubulin, alpha 1b | 0.03 | 9.7e-01 |
| 10381 | TUBB3 | tubulin, beta 3 class III | -0.17 | 7.3e-01 |
| 10382 | TUBB4A | tubulin, beta 4A class IVa | -0.14 | 5.2e-01 |
| 10383 | TUBB4B | tubulin, beta 4B class IVb | -0.16 | 7.6e-01 |
| 10552 | ARPC1A | actin related protein 2/3 complex, subunit 1A, 41kDa | 0.00 | 1.0e+00 |
| 10971 | YWHAQ | tyrosine 3-monooxygenase/tryptophan 5-monooxygenase activation protein, theta | 0.18 | 6.2e-01 |
| 1499 | CTNNB1 | catenin (cadherin-associated protein), beta 1, 88kDa | 0.26 | 5.5e-01 |
| 2017 | CTTN | cortactin | -0.10 | 5.1e-01 |
| 203068 | TUBB | tubulin, beta class I | -0.21 | 4.1e-01 |
| 23643 | LY96 | lymphocyte antigen 96 | 0.04 | 9.5e-01 |
| 25 | ABL1 | ABL proto-oncogene 1, non-receptor tyrosine kinase | 0.64 | 1.8e-05 |
| 2534 | FYN | FYN proto-oncogene, Src family tyrosine kinase | 0.47 | 1.4e-03 |
| 3059 | HCLS1 | hematopoietic cell-specific Lyn substrate 1 | -0.05 | 9.4e-01 |
| 347733 | TUBB2B | tubulin, beta 2B class IIb | -0.02 | 9.6e-01 |
| 3688 | ITGB1 | integrin, beta 1 (fibronectin receptor, beta polypeptide, antigen CD29 includes MDF2, MSK12) | 0.28 | 7.8e-02 |
| 387 | RHOA | ras homolog family member A | 0.27 | 1.2e-01 |
| 3875 | KRT18 | keratin 18, type I | 0.19 | 7.4e-01 |
| 4690 | NCK1 | NCK adaptor protein 1 | -0.01 | 9.9e-01 |
| 4691 | NCL | nucleolin | -0.03 | 9.6e-01 |
| 5578 | PRKCA | protein kinase C, alpha | 0.01 | 9.6e-01 |
| 60 | ACTB | actin, beta | 0.01 | 1.0e+00 |
| 6093 | ROCK1 | Rho-associated, coiled-coil containing protein kinase 1 | 0.00 | 1.0e+00 |
| 71 | ACTG1 | actin gamma 1 | -0.03 | 9.6e-01 |
| 7100 | TLR5 | toll-like receptor 5 | 0.10 | 3.0e-01 |
| 7277 | TUBA4A | tubulin, alpha 4a | 0.95 | 3.4e-03 |
| 7278 | TUBA3C | tubulin, alpha 3c | -0.08 | 8.8e-01 |
| 7280 | TUBB2A | tubulin, beta 2A class IIa | 0.12 | 9.5e-01 |
| 7430 | EZR | ezrin | -0.15 | 9.0e-01 |
| 7454 | WAS | Wiskott-Aldrich syndrome | -0.06 | 7.8e-01 |
| 7534 | YWHAZ | tyrosine 3-monooxygenase/tryptophan 5-monooxygenase activation protein, zeta | -0.13 | 6.6e-01 |
| 8440 | NCK2 | NCK adaptor protein 2 | -0.09 | 8.0e-01 |
| 8976 | WASL | Wiskott-Aldrich syndrome-like | -0.02 | 9.6e-01 |
| 9181 | ARHGEF2 | Rho/Rac guanine nucleotide exchange factor (GEF) 2 | 0.01 | 9.9e-01 |
| 929 | CD14 | CD14 molecule | 0.18 | 3.0e-01 |
| 9475 | ROCK2 | Rho-associated, coiled-coil containing protein kinase 2 | 0.11 | 7.8e-01 |
| 998 | CDC42 | cell division cycle 42 | 0.09 | 9.3e-01 |
| 999 | CDH1 | cadherin 1, type 1, E-cadherin (epithelial) | -0.03 | 8.3e-01 |

| ENTREZID | SYMBOL | GENENAME | FC | ADJ.PVAL |
| --- | --- | --- | --- | --- |

(Page generated on Tue Aug 25 20:48:53 2015 by ReportingTools 2.9.1 and hwriter 1.3.2)
