# Supplementary material for: Bioconductor’s EnrichmentBrowser: seamless navigation through combined results of set- & network-based enrichment analysis
Source: BMC Bioinformatics. 2016 Jan 20;17:45. doi: 10.1186/s12859-016-0884-1 (PMC4721010; doi:10.1186/s12859-016-0884-1)
Supplement: Supplementary file 2 — EnrichmentBrowser output (ALL microarray data). Unzip and open the contained index.html in the browser to view the contents of this file (tested with Firefox 39.0). (ZIP 2775 kb) [file 12859_2016_884_MOESM2_ESM.zip › hsa05134.html]

hsa05134: Gene Report


## hsa05134: Gene Report

| ENTREZID | SYMBOL | GENENAME | FC | ADJ.PVAL |
| --- | --- | --- | --- | --- |
| ENTREZID | SYMBOL | GENENAME | FC | ADJ.PVAL |
| 10767 | HBS1L | HBS1-like translational GTPase | 0.66 | 0.0120 |
| 1195 | CLK1 | CDC-like kinase 1 | 0.10 | 0.9300 |
| 1378 | CR1 | complement component (3b/4b) receptor 1 (Knops blood group) | -0.02 | 0.9500 |
| 1915 | EEF1A1 | eukaryotic translation elongation factor 1 alpha 1 | -0.09 | 0.8100 |
| 1917 | EEF1A2 | eukaryotic translation elongation factor 1 alpha 2 | -0.13 | 0.5400 |
| 1937 | EEF1G | eukaryotic translation elongation factor 1 gamma | -0.15 | 0.5100 |
| 2919 | CXCL1 | chemokine (C-X-C motif) ligand 1 (melanoma growth stimulating activity, alpha) | 0.05 | 0.9000 |
| 2920 | CXCL2 | chemokine (C-X-C motif) ligand 2 | 0.07 | 0.9700 |
| 2921 | CXCL3 | chemokine (C-X-C motif) ligand 3 | 0.15 | 0.7400 |
| 317 | APAF1 | apoptotic peptidase activating factor 1 | -0.01 | 0.9900 |
| 3297 | HSF1 | heat shock transcription factor 1 | -0.05 | 0.8900 |
| 3303 | HSPA1A | heat shock 70kDa protein 1A | 0.31 | 0.7500 |
| 3304 | HSPA1B | heat shock 70kDa protein 1B | 0.01 | 0.9900 |
| 3305 | HSPA1L | heat shock 70kDa protein 1-like | 0.01 | 0.9700 |
| 3306 | HSPA2 | heat shock 70kDa protein 2 | 0.00 | 1.0000 |
| 3310 | HSPA6 | heat shock 70kDa protein 6 (HSP70B') | 0.14 | 0.6900 |
| 3312 | HSPA8 | heat shock 70kDa protein 8 | 0.10 | 0.8500 |
| 3329 | HSPD1 | heat shock 60kDa protein 1 (chaperonin) | 0.07 | 0.9500 |
| 3553 | IL1B | interleukin 1, beta | -0.02 | 0.9900 |
| 3569 | IL6 | interleukin 6 | 0.15 | 0.8600 |
| 3576 | CXCL8 | chemokine (C-X-C motif) ligand 8 | 0.50 | 0.6200 |
| 3592 | IL12A | interleukin 12A | 0.21 | 0.1900 |
| 3593 | IL12B | interleukin 12B | 0.06 | 0.6300 |
| 3606 | IL18 | interleukin 18 | 0.16 | 0.5300 |
| 3684 | ITGAM | integrin, alpha M (complement component 3 receptor 3 subunit) | 0.16 | 0.3800 |
| 3689 | ITGB2 | integrin, beta 2 (complement component 3 receptor 3 and 4 subunit) | 0.31 | 0.6400 |
| 375 | ARF1 | ADP-ribosylation factor 1 | -0.01 | 0.9900 |
| 4615 | MYD88 | myeloid differentiation primary response 88 | -0.11 | 0.7800 |
| 4671 | NAIP | NLR family, apoptosis inhibitory protein | 0.03 | 0.9600 |
| 4790 | NFKB1 | nuclear factor of kappa light polypeptide gene enhancer in B-cells 1 | 0.35 | 0.2300 |
| 4791 | NFKB2 | nuclear factor of kappa light polypeptide gene enhancer in B-cells 2 (p49/p100) | -0.02 | 0.9700 |
| 4792 | NFKBIA | nuclear factor of kappa light polypeptide gene enhancer in B-cells inhibitor, alpha | 0.71 | 0.0410 |
| 54205 | CYCS | cytochrome c, somatic | -0.22 | 0.7200 |
| 56681 | SAR1A | secretion associated, Ras related GTPase 1A | -0.18 | 0.4500 |
| 5861 | RAB1A | RAB1A, member RAS oncogene family | 0.25 | 0.1600 |
| 5970 | RELA | v-rel avian reticuloendotheliosis viral oncogene homolog A | -0.04 | 0.9500 |
| 664 | BNIP3 | BCL2/adenovirus E1B 19kDa interacting protein 3 | -0.16 | 0.6800 |
| 7097 | TLR2 | toll-like receptor 2 | 0.18 | 0.7800 |
| 7100 | TLR5 | toll-like receptor 5 | 0.10 | 0.3000 |
| 7124 | TNF | tumor necrosis factor | 0.12 | 0.7100 |
| 7415 | VCP | valosin containing protein | -0.05 | 0.9400 |
| 834 | CASP1 | caspase 1, apoptosis-related cysteine peptidase | 0.07 | 0.8900 |
| 836 | CASP3 | caspase 3, apoptosis-related cysteine peptidase | 0.20 | 0.4900 |
| 840 | CASP7 | caspase 7, apoptosis-related cysteine peptidase | 0.17 | 0.6800 |
| 841 | CASP8 | caspase 8, apoptosis-related cysteine peptidase | 0.48 | 0.0016 |
| 842 | CASP9 | caspase 9, apoptosis-related cysteine peptidase | -0.08 | 0.8100 |
| 929 | CD14 | CD14 molecule | 0.18 | 0.3000 |
| 9554 | SEC22B | SEC22 vesicle trafficking protein homolog B (S. cerevisiae) (gene/pseudogene) | 0.05 | 0.9200 |

| ENTREZID | SYMBOL | GENENAME | FC | ADJ.PVAL |
| --- | --- | --- | --- | --- |

(Page generated on Tue Aug 25 20:48:54 2015 by ReportingTools 2.9.1 and hwriter 1.3.2)
