# Supplementary material for: Bioconductor’s EnrichmentBrowser: seamless navigation through combined results of set- & network-based enrichment analysis
Source: BMC Bioinformatics. 2016 Jan 20;17:45. doi: 10.1186/s12859-016-0884-1 (PMC4721010; doi:10.1186/s12859-016-0884-1)
Supplement: Supplementary file 2 — EnrichmentBrowser output (ALL microarray data). Unzip and open the contained index.html in the browser to view the contents of this file (tested with Firefox 39.0). (ZIP 2775 kb) [file 12859_2016_884_MOESM2_ESM.zip › hsa05202.html]

hsa05202: Gene Report


## hsa05202: Gene Report

| ENTREZID | SYMBOL | GENENAME | FC | ADJ.PVAL |
| --- | --- | --- | --- | --- |
| ENTREZID | SYMBOL | GENENAME | FC | ADJ.PVAL |
| 1025 | CDK9 | cyclin-dependent kinase 9 | 0.20 | 0.6500 |
| 1026 | CDKN1A | cyclin-dependent kinase inhibitor 1A (p21, Cip1) | 0.37 | 0.5500 |
| 1027 | CDKN1B | cyclin-dependent kinase inhibitor 1B (p27, Kip1) | 0.00 | 1.0000 |
| 1031 | CDKN2C | cyclin-dependent kinase inhibitor 2C (p18, inhibits CDK4) | 0.33 | 0.0190 |
| 1050 | CEBPA | CCAAT/enhancer binding protein (C/EBP), alpha | -0.21 | 0.1700 |
| 1051 | CEBPB | CCAAT/enhancer binding protein (C/EBP), beta | 0.27 | 0.8700 |
| 1053 | CEBPE | CCAAT/enhancer binding protein (C/EBP), epsilon | -0.02 | 0.9700 |
| 1236 | CCR7 | chemokine (C-C motif) receptor 7 | 0.06 | 0.9600 |
| 1436 | CSF1R | colony stimulating factor 1 receptor | 0.09 | 0.7700 |
| 1437 | CSF2 | colony stimulating factor 2 (granulocyte-macrophage) | -0.03 | 0.9000 |
| 1649 | DDIT3 | DNA-damage-inducible transcript 3 | 0.10 | 0.9400 |
| 1655 | DDX5 | DEAD (Asp-Glu-Ala-Asp) box helicase 5 | 0.16 | 0.7400 |
| 171558 | PTCRA | pre T-cell antigen receptor alpha | -0.02 | 0.9600 |
| 1848 | DUSP6 | dual specificity phosphatase 6 | 0.87 | 0.0410 |
| 1991 | ELANE | elastase, neutrophil expressed | 0.47 | 0.1300 |
| 2005 | ELK4 | ELK4, ETS-domain protein (SRF accessory protein 1) | 0.04 | 0.9000 |
| 2078 | ERG | v-ets avian erythroblastosis virus E26 oncogene homolog | 0.00 | 1.0000 |
| 2115 | ETV1 | ets variant 1 | -0.03 | 0.9000 |
| 2118 | ETV4 | ets variant 4 | -0.05 | 0.7800 |
| 2119 | ETV5 | ets variant 5 | 0.37 | 0.3000 |
| 2120 | ETV6 | ets variant 6 | -0.07 | 0.9200 |
| 2130 | EWSR1 | EWS RNA-binding protein 1 | -0.11 | 0.8200 |
| 2138 | EYA1 | EYA transcriptional coactivator and phosphatase 1 | 0.00 | 1.0000 |
| 2209 | FCGR1A | Fc fragment of IgG, high affinity Ia, receptor (CD64) | 0.06 | 0.8700 |
| 221037 | JMJD1C | jumonji domain containing 1C | 0.22 | 0.6300 |
| 2308 | FOXO1 | forkhead box O1 | 0.32 | 0.7000 |
| 2313 | FLI1 | Fli-1 proto-oncogene, ETS transcription factor | -0.16 | 0.8500 |
| 2321 | FLT1 | fms-related tyrosine kinase 1 | -0.01 | 0.9900 |
| 2322 | FLT3 | fms-related tyrosine kinase 3 | -0.58 | 0.3700 |
| 2521 | FUS | FUS RNA binding protein | -0.18 | 0.7500 |
| 2530 | FUT8 | fucosyltransferase 8 (alpha (1,6) fucosyltransferase) | 0.03 | 0.9600 |
| 26471 | NUPR1 | nuclear protein, transcriptional regulator, 1 | -0.07 | 0.7400 |
| 2892 | GRIA3 | glutamate receptor, ionotropic, AMPA 3 | 0.03 | 0.8500 |
| 3002 | GZMB | granzyme B (granzyme 2, cytotoxic T-lymphocyte-associated serine esterase 1) | 0.12 | 0.9000 |
| 3020 | H3F3A | H3 histone, family 3A | -0.09 | 0.7500 |
| 3065 | HDAC1 | histone deacetylase 1 | 0.00 | 0.9900 |
| 3066 | HDAC2 | histone deacetylase 2 | 0.01 | 1.0000 |
| 3087 | HHEX | hematopoietically expressed homeobox | 0.27 | 0.7000 |
| 3195 | TLX1 | T-cell leukemia homeobox 1 | -0.03 | 0.9400 |
| 3205 | HOXA9 | homeobox A9 | -0.02 | 0.9700 |
| 3206 | HOXA10 | homeobox A10 | 0.03 | 0.9400 |
| 3207 | HOXA11 | homeobox A11 | -0.04 | 0.8400 |
| 3248 | HPGD | hydroxyprostaglandin dehydrogenase 15-(NAD) | 0.00 | 0.9900 |
| 330 | BIRC3 | baculoviral IAP repeat containing 3 | 0.39 | 0.2800 |
| 3398 | ID2 | inhibitor of DNA binding 2, dominant negative helix-loop-helix protein | 0.14 | 0.8300 |
| 3479 | IGF1 | insulin-like growth factor 1 (somatomedin C) | -0.01 | 0.9600 |
| 3480 | IGF1R | insulin-like growth factor 1 receptor | -0.09 | 0.5700 |
| 3486 | IGFBP3 | insulin-like growth factor binding protein 3 | -0.01 | 0.9900 |
| 3560 | IL2RB | interleukin 2 receptor, beta | 0.24 | 0.4600 |
| 3562 | IL3 | interleukin 3 | 0.01 | 0.9900 |
| 3569 | IL6 | interleukin 6 | 0.15 | 0.8600 |
| 3576 | CXCL8 | chemokine (C-X-C motif) ligand 8 | 0.50 | 0.6200 |
| 3684 | ITGAM | integrin, alpha M (complement component 3 receptor 3 subunit) | 0.16 | 0.3800 |
| 3695 | ITGB7 | integrin, beta 7 | 0.09 | 0.9000 |
| 3728 | JUP | junction plakoglobin | 0.01 | 0.9900 |
| 4005 | LMO2 | LIM domain only 2 (rhombotin-like 1) | -0.15 | 0.9200 |
| 4066 | LYL1 | lymphoblastic leukemia associated hematopoiesis regulator 1 | -0.08 | 0.9000 |
| 4086 | SMAD1 | SMAD family member 1 | -0.02 | 1.0000 |
| 4094 | MAF | v-maf avian musculoaponeurotic fibrosarcoma oncogene homolog | 0.18 | 0.7800 |
| 4149 | MAX | MYC associated factor X | -0.08 | 0.8700 |
| 4193 | MDM2 | MDM2 proto-oncogene, E3 ubiquitin protein ligase | 0.14 | 0.1700 |
| 4208 | MEF2C | myocyte enhancer factor 2C | -0.02 | 0.9900 |
| 4211 | MEIS1 | Meis homeobox 1 | -0.01 | 0.9900 |
| 4221 | MEN1 | multiple endocrine neoplasia I | -0.12 | 0.7100 |
| 4233 | MET | MET proto-oncogene, receptor tyrosine kinase | 0.01 | 0.9700 |
| 4291 | MLF1 | myeloid leukemia factor 1 | 0.02 | 0.9400 |
| 4297 | KMT2A | lysine (K)-specific methyltransferase 2A | 0.07 | 0.9000 |
| 4298 | MLLT1 | myeloid/lymphoid or mixed-lineage leukemia (trithorax homolog, Drosophila); translocated to, 1 | -0.02 | 0.9700 |
| 4299 | AFF1 | AF4/FMR2 family, member 1 | 0.24 | 0.1700 |
| 4300 | MLLT3 | myeloid/lymphoid or mixed-lineage leukemia (trithorax homolog, Drosophila); translocated to, 3 | 0.38 | 0.0300 |
| 4314 | MMP3 | matrix metallopeptidase 3 | 0.03 | 0.9100 |
| 4318 | MMP9 | matrix metallopeptidase 9 | 0.11 | 0.7000 |
| 4353 | MPO | myeloperoxidase | 0.82 | 0.1100 |
| 4609 | MYC | v-myc avian myelocytomatosis viral oncogene homolog | -0.16 | 0.6200 |
| 4613 | MYCN | v-myc avian myelocytomatosis viral oncogene neuroblastoma derived homolog | -0.02 | 0.9500 |
| 466 | ATF1 | activating transcription factor 1 | -0.12 | 0.6400 |
| 472 | ATM | ATM serine/threonine kinase | -0.01 | 0.9900 |
| 4790 | NFKB1 | nuclear factor of kappa light polypeptide gene enhancer in B-cells 1 | 0.35 | 0.2300 |
| 4804 | NGFR | nerve growth factor receptor | -0.07 | 0.7400 |
| 4914 | NTRK1 | neurotrophic tyrosine kinase, receptor, type 1 | -0.12 | 0.5800 |
| 5077 | PAX3 | paired box 3 | -0.04 | 0.9100 |
| 5079 | PAX5 | paired box 5 | 0.02 | 0.9700 |
| 5081 | PAX7 | paired box 7 | -0.03 | 0.9000 |
| 5087 | PBX1 | pre-B-cell leukemia homeobox 1 | -0.05 | 0.9300 |
| 5090 | PBX3 | pre-B-cell leukemia homeobox 3 | 0.68 | 0.0140 |
| 5154 | PDGFA | platelet-derived growth factor alpha polypeptide | -0.17 | 0.2700 |
| 5218 | CDK14 | cyclin-dependent kinase 14 | 0.86 | 0.0300 |
| 5327 | PLAT | plasminogen activator, tissue | 0.00 | 0.9900 |
| 5328 | PLAU | plasminogen activator, urokinase | 0.40 | 0.0720 |
| 5371 | PML | promyelocytic leukemia | 0.00 | 0.9900 |
| 5468 | PPARG | peroxisome proliferator-activated receptor gamma | 0.02 | 0.9600 |
| 54738 | FEV | FEV (ETS oncogene family) | -0.05 | 0.8900 |
| 5546 | PRCC | papillary renal cell carcinoma (translocation-associated) | -0.03 | 0.9300 |
| 55589 | BMP2K | BMP2 inducible kinase | 0.55 | 0.1700 |
| 5747 | PTK2 | protein tyrosine kinase 2 | 0.01 | 0.9900 |
| 5914 | RARA | retinoic acid receptor, alpha | -0.04 | 0.9400 |
| 5966 | REL | v-rel avian reticuloendotheliosis viral oncogene homolog | 0.21 | 0.4100 |
| 597 | BCL2A1 | BCL2-related protein A1 | 0.42 | 0.0710 |
| 5970 | RELA | v-rel avian reticuloendotheliosis viral oncogene homolog A | -0.04 | 0.9500 |
| 598 | BCL2L1 | BCL2-like 1 | -0.03 | 0.9700 |
| 604 | BCL6 | B-cell CLL/lymphoma 6 | 0.26 | 0.1400 |
| 6256 | RXRA | retinoid X receptor, alpha | -0.03 | 0.9500 |
| 6257 | RXRB | retinoid X receptor, beta | 0.07 | 0.7900 |
| 6258 | RXRG | retinoid X receptor, gamma | -0.04 | 0.9200 |
| 648 | BMI1 | BMI1 proto-oncogene, polycomb ring finger | -0.05 | 0.9600 |
| 6495 | SIX1 | SIX homeobox 1 | -0.04 | 0.9200 |
| 6667 | SP1 | Sp1 transcription factor | 0.01 | 0.9900 |
| 6688 | SPI1 | Spi-1 proto-oncogene | -0.09 | 0.8500 |
| 6692 | SPINT1 | serine peptidase inhibitor, Kunitz type 1 | -0.09 | 0.6300 |
| 6756 | SSX1 | synovial sarcoma, X breakpoint 1 | -0.04 | 0.9300 |
| 6757 | SSX2 | synovial sarcoma, X breakpoint 2 | 0.01 | 0.9500 |
| 6760 | SS18 | synovial sarcoma translocation, chromosome 18 | -0.02 | 0.9700 |
| 6929 | TCF3 | transcription factor 3 | 0.00 | 1.0000 |
| 6935 | ZEB1 | zinc finger E-box binding homeobox 1 | 1.20 | 0.0018 |
| 7030 | TFE3 | transcription factor binding to IGHM enhancer 3 | 0.01 | 0.9800 |
| 7048 | TGFBR2 | transforming growth factor, beta receptor II (70/80kDa) | 0.04 | 0.9700 |
| 7102 | TSPAN7 | tetraspanin 7 | 0.85 | 0.0130 |
| 7113 | TMPRSS2 | transmembrane protease, serine 2 | -0.04 | 0.8600 |
| 7157 | TP53 | tumor protein p53 | -0.10 | 0.7400 |
| 7185 | TRAF1 | TNF receptor-associated factor 1 | 0.08 | 0.6800 |
| 7403 | KDM6A | lysine (K)-specific demethylase 6A | 0.05 | 0.9000 |
| 7404 | UTY | ubiquitously transcribed tetratricopeptide repeat containing, Y-linked | -0.09 | 0.9300 |
| 7468 | WHSC1 | Wolf-Hirschhorn syndrome candidate 1 | -0.04 | 0.9600 |
| 7490 | WT1 | Wilms tumor 1 | -0.14 | 0.3200 |
| 7704 | ZBTB16 | zinc finger and BTB domain containing 16 | 0.03 | 0.9900 |
| 7709 | ZBTB17 | zinc finger and BTB domain containing 17 | 0.05 | 0.9200 |
| 7849 | PAX8 | paired box 8 | -0.02 | 0.9600 |
| 7850 | IL1R2 | interleukin 1 receptor, type II | -0.01 | 0.9900 |
| 8013 | NR4A3 | nuclear receptor subfamily 4, group A, member 3 | -0.29 | 0.5100 |
| 8091 | HMGA2 | high mobility group AT-hook 2 | -0.01 | 0.9800 |
| 8148 | TAF15 | TAF15 RNA polymerase II, TATA box binding protein (TBP)-associated factor, 68kDa | 0.00 | 0.9900 |
| 8350 | HIST1H3A | histone cluster 1, H3a | 0.14 | 0.9000 |
| 8351 | HIST1H3D | histone cluster 1, H3d | -0.17 | 0.9000 |
| 8353 | HIST1H3E | histone cluster 1, H3e | 0.02 | 0.9500 |
| 8354 | HIST1H3I | histone cluster 1, H3i | -0.07 | 0.7900 |
| 84444 | DOT1L | DOT1-like histone H3K79 methyltransferase | -0.03 | 0.9200 |
| 8464 | SUPT3H | suppressor of Ty 3 homolog (S. cerevisiae) | 0.10 | 0.7900 |
| 860 | RUNX2 | runt-related transcription factor 2 | -0.01 | 0.9800 |
| 861 | RUNX1 | runt-related transcription factor 1 | 0.01 | 0.9900 |
| 862 | RUNX1T1 | runt-related transcription factor 1; translocated to, 1 (cyclin D-related) | 0.03 | 0.8800 |
| 8842 | PROM1 | prominin 1 | 0.57 | 0.6000 |
| 8861 | LDB1 | LIM domain binding 1 | 0.03 | 0.9200 |
| 8864 | PER2 | period circadian clock 2 | 0.05 | 0.9500 |
| 8900 | CCNA1 | cyclin A1 | -0.66 | 0.0880 |
| 8938 | BAIAP3 | BAI1-associated protein 3 | 0.05 | 0.8800 |
| 894 | CCND2 | cyclin D2 | 0.35 | 0.3600 |
| 904 | CCNT1 | cyclin T1 | -0.06 | 0.8500 |
| 905 | CCNT2 | cyclin T2 | 0.19 | 0.5000 |
| 929 | CD14 | CD14 molecule | 0.18 | 0.3000 |
| 942 | CD86 | CD86 molecule | 0.06 | 0.8200 |
| 958 | CD40 | CD40 molecule, TNF receptor superfamily member 5 | 0.10 | 0.4500 |
| 9611 | NCOR1 | nuclear receptor corepressor 1 | -0.15 | 0.4000 |
| 9915 | ARNT2 | aryl-hydrocarbon receptor nuclear translocator 2 | -0.03 | 0.9800 |

| ENTREZID | SYMBOL | GENENAME | FC | ADJ.PVAL |
| --- | --- | --- | --- | --- |

(Page generated on Tue Aug 25 20:46:20 2015 by ReportingTools 2.9.1 and hwriter 1.3.2)
