# Supplementary material for: Bioconductor’s EnrichmentBrowser: seamless navigation through combined results of set- & network-based enrichment analysis
Source: BMC Bioinformatics. 2016 Jan 20;17:45. doi: 10.1186/s12859-016-0884-1 (PMC4721010; doi:10.1186/s12859-016-0884-1)
Supplement: Supplementary file 2 — EnrichmentBrowser output (ALL microarray data). Unzip and open the contained index.html in the browser to view the contents of this file (tested with Firefox 39.0). (ZIP 2775 kb) [file 12859_2016_884_MOESM2_ESM.zip › hsa05217.html]

hsa05217: Gene Report


## hsa05217: Gene Report

| ENTREZID | SYMBOL | GENENAME | FC | ADJ.PVAL |
| --- | --- | --- | --- | --- |
| ENTREZID | SYMBOL | GENENAME | FC | ADJ.PVAL |
| 10297 | APC2 | adenomatosis polyposis coli 2 | -0.02 | 0.9400 |
| 1499 | CTNNB1 | catenin (cadherin-associated protein), beta 1, 88kDa | 0.26 | 0.5500 |
| 1855 | DVL1 | dishevelled segment polarity protein 1 | -0.07 | 0.8800 |
| 1857 | DVL3 | dishevelled segment polarity protein 3 | -0.16 | 0.3400 |
| 2535 | FZD2 | frizzled class receptor 2 | -0.25 | 0.1600 |
| 2735 | GLI1 | GLI family zinc finger 1 | -0.09 | 0.6500 |
| 2736 | GLI2 | GLI family zinc finger 2 | 0.03 | 0.8500 |
| 2737 | GLI3 | GLI family zinc finger 3 | -0.04 | 0.9200 |
| 2932 | GSK3B | glycogen synthase kinase 3 beta | 0.15 | 0.3100 |
| 324 | APC | adenomatous polyposis coli | 0.01 | 0.9600 |
| 51176 | LEF1 | lymphoid enhancer-binding factor 1 | 0.93 | 0.0280 |
| 54361 | WNT4 | wingless-type MMTV integration site family, member 4 | -0.02 | 0.9700 |
| 5727 | PTCH1 | patched 1 | -0.01 | 0.9700 |
| 6469 | SHH | sonic hedgehog | -0.06 | 0.8500 |
| 650 | BMP2 | bone morphogenetic protein 2 | 0.43 | 0.6300 |
| 652 | BMP4 | bone morphogenetic protein 4 | 0.02 | 0.9500 |
| 6608 | SMO | smoothened, frizzled class receptor | -0.03 | 0.9400 |
| 6932 | TCF7 | transcription factor 7 (T-cell specific, HMG-box) | 0.41 | 0.1200 |
| 6934 | TCF7L2 | transcription factor 7-like 2 (T-cell specific, HMG-box) | 0.18 | 0.6200 |
| 7157 | TP53 | tumor protein p53 | -0.10 | 0.7400 |
| 7471 | WNT1 | wingless-type MMTV integration site family, member 1 | -0.07 | 0.8500 |
| 7472 | WNT2 | wingless-type MMTV integration site family member 2 | 0.02 | 0.9600 |
| 7474 | WNT5A | wingless-type MMTV integration site family, member 5A | 0.05 | 0.8000 |
| 7475 | WNT6 | wingless-type MMTV integration site family, member 6 | -0.03 | 0.9600 |
| 7476 | WNT7A | wingless-type MMTV integration site family, member 7A | -0.03 | 0.9000 |
| 7479 | WNT8B | wingless-type MMTV integration site family, member 8B | -0.01 | 0.9900 |
| 7480 | WNT10B | wingless-type MMTV integration site family, member 10B | 0.01 | 0.9900 |
| 7481 | WNT11 | wingless-type MMTV integration site family, member 11 | 0.01 | 0.9800 |
| 7482 | WNT2B | wingless-type MMTV integration site family, member 2B | 0.05 | 0.8200 |
| 7855 | FZD5 | frizzled class receptor 5 | 0.08 | 0.7400 |
| 8312 | AXIN1 | axin 1 | -0.05 | 0.7900 |
| 8321 | FZD1 | frizzled class receptor 1 | -0.01 | 0.9900 |
| 8323 | FZD6 | frizzled class receptor 6 | 1.18 | 0.0012 |
| 8324 | FZD7 | frizzled class receptor 7 | 0.08 | 0.9200 |
| 8326 | FZD9 | frizzled class receptor 9 | -0.07 | 0.7700 |

| ENTREZID | SYMBOL | GENENAME | FC | ADJ.PVAL |
| --- | --- | --- | --- | --- |

(Page generated on Tue Aug 25 20:50:56 2015 by ReportingTools 2.9.1 and hwriter 1.3.2)
