# Supplementary material for: Bioconductor’s EnrichmentBrowser: seamless navigation through combined results of set- & network-based enrichment analysis
Source: BMC Bioinformatics. 2016 Jan 20;17:45. doi: 10.1186/s12859-016-0884-1 (PMC4721010; doi:10.1186/s12859-016-0884-1)
Supplement: Supplementary file 2 — EnrichmentBrowser output (ALL microarray data). Unzip and open the contained index.html in the browser to view the contents of this file (tested with Firefox 39.0). (ZIP 2775 kb) [file 12859_2016_884_MOESM2_ESM.zip › hsa05416.html]

hsa05416: Gene Report


## hsa05416: Gene Report

| ENTREZID | SYMBOL | GENENAME | FC | ADJ.PVAL |
| --- | --- | --- | --- | --- |
| ENTREZID | SYMBOL | GENENAME | FC | ADJ.PVAL |
| 1525 | CXADR | coxsackie virus and adenovirus receptor | -0.06 | 8.5e-01 |
| 1604 | CD55 | CD55 molecule, decay accelerating factor for complement (Cromer blood group) | 0.33 | 7.2e-01 |
| 1605 | DAG1 | dystroglycan 1 (dystrophin-associated glycoprotein 1) | -0.12 | 5.3e-01 |
| 1756 | DMD | dystrophin | 0.19 | 5.6e-01 |
| 1981 | EIF4G1 | eukaryotic translation initiation factor 4 gamma, 1 | -0.08 | 9.3e-01 |
| 1982 | EIF4G2 | eukaryotic translation initiation factor 4 gamma, 2 | 0.06 | 9.3e-01 |
| 25 | ABL1 | ABL proto-oncogene 1, non-receptor tyrosine kinase | 0.64 | 1.8e-05 |
| 2534 | FYN | FYN proto-oncogene, Src family tyrosine kinase | 0.47 | 1.4e-03 |
| 27 | ABL2 | ABL proto-oncogene 2, non-receptor tyrosine kinase | -0.06 | 8.6e-01 |
| 3105 | HLA-A | major histocompatibility complex, class I, A | 0.17 | 5.0e-01 |
| 3106 | HLA-B | major histocompatibility complex, class I, B | 0.10 | 7.4e-01 |
| 3108 | HLA-DMA | major histocompatibility complex, class II, DM alpha | 0.31 | 4.1e-01 |
| 3109 | HLA-DMB | major histocompatibility complex, class II, DM beta | 0.33 | 5.0e-01 |
| 3111 | HLA-DOA | major histocompatibility complex, class II, DO alpha | -0.01 | 9.9e-01 |
| 3112 | HLA-DOB | major histocompatibility complex, class II, DO beta | 0.10 | 8.5e-01 |
| 3113 | HLA-DPA1 | major histocompatibility complex, class II, DP alpha 1 | 0.25 | 4.1e-01 |
| 3115 | HLA-DPB1 | major histocompatibility complex, class II, DP beta 1 | 0.15 | 7.8e-01 |
| 3117 | HLA-DQA1 | major histocompatibility complex, class II, DQ alpha 1 | 0.37 | 6.4e-01 |
| 3119 | HLA-DQB1 | major histocompatibility complex, class II, DQ beta 1 | 0.24 | 7.8e-01 |
| 3122 | HLA-DRA | major histocompatibility complex, class II, DR alpha | 0.19 | 3.0e-01 |
| 3123 | HLA-DRB1 | major histocompatibility complex, class II, DR beta 1 | 0.43 | 6.6e-01 |
| 3125 | HLA-DRB3 | major histocompatibility complex, class II, DR beta 3 | 0.26 | 7.0e-01 |
| 3127 | HLA-DRB5 | major histocompatibility complex, class II, DR beta 5 | 0.25 | 7.3e-01 |
| 3133 | HLA-E | major histocompatibility complex, class I, E | 0.00 | 1.0e+00 |
| 3134 | HLA-F | major histocompatibility complex, class I, F | 0.15 | 7.8e-01 |
| 3135 | HLA-G | major histocompatibility complex, class I, G | -0.02 | 9.9e-01 |
| 3383 | ICAM1 | intercellular adhesion molecule 1 | 0.33 | 1.9e-01 |
| 3683 | ITGAL | integrin, alpha L (antigen CD11A (p180), lymphocyte function-associated antigen 1; alpha polypeptide) | 0.12 | 7.6e-01 |
| 3689 | ITGB2 | integrin, beta 2 (complement component 3 receptor 3 and 4 subunit) | 0.31 | 6.4e-01 |
| 3908 | LAMA2 | laminin, alpha 2 | -0.05 | 8.6e-01 |
| 4624 | MYH6 | myosin, heavy chain 6, cardiac muscle, alpha | -0.04 | 8.5e-01 |
| 4625 | MYH7 | myosin, heavy chain 7, cardiac muscle, beta | 0.01 | 9.8e-01 |
| 54205 | CYCS | cytochrome c, somatic | -0.22 | 7.2e-01 |
| 5551 | PRF1 | perforin 1 (pore forming protein) | 0.08 | 8.9e-01 |
| 5879 | RAC1 | ras-related C3 botulinum toxin substrate 1 (rho family, small GTP binding protein Rac1) | -0.03 | 9.8e-01 |
| 5880 | RAC2 | ras-related C3 botulinum toxin substrate 2 (rho family, small GTP binding protein Rac2) | -0.09 | 9.3e-01 |
| 5881 | RAC3 | ras-related C3 botulinum toxin substrate 3 (rho family, small GTP binding protein Rac3) | -0.03 | 9.3e-01 |
| 595 | CCND1 | cyclin D1 | -0.01 | 9.8e-01 |
| 60 | ACTB | actin, beta | 0.01 | 1.0e+00 |
| 637 | BID | BH3 interacting domain death agonist | 0.03 | 9.4e-01 |
| 6442 | SGCA | sarcoglycan, alpha (50kDa dystrophin-associated glycoprotein) | 0.01 | 9.9e-01 |
| 6443 | SGCB | sarcoglycan, beta (43kDa dystrophin-associated glycoprotein) | 0.05 | 8.0e-01 |
| 6444 | SGCD | sarcoglycan, delta (35kDa dystrophin-associated glycoprotein) | -0.02 | 9.3e-01 |
| 6445 | SGCG | sarcoglycan, gamma (35kDa dystrophin-associated glycoprotein) | -0.05 | 8.9e-01 |
| 71 | ACTG1 | actin gamma 1 | -0.03 | 9.6e-01 |
| 836 | CASP3 | caspase 3, apoptosis-related cysteine peptidase | 0.20 | 4.9e-01 |
| 841 | CASP8 | caspase 8, apoptosis-related cysteine peptidase | 0.48 | 1.6e-03 |
| 842 | CASP9 | caspase 9, apoptosis-related cysteine peptidase | -0.08 | 8.1e-01 |
| 857 | CAV1 | caveolin 1, caveolae protein, 22kDa | 0.86 | 4.6e-03 |
| 8672 | EIF4G3 | eukaryotic translation initiation factor 4 gamma, 3 | 0.12 | 8.9e-01 |
| 940 | CD28 | CD28 molecule | 0.01 | 9.9e-01 |
| 941 | CD80 | CD80 molecule | 0.08 | 5.6e-01 |
| 942 | CD86 | CD86 molecule | 0.06 | 8.2e-01 |
| 958 | CD40 | CD40 molecule, TNF receptor superfamily member 5 | 0.10 | 4.5e-01 |
| 959 | CD40LG | CD40 ligand | 0.00 | 9.9e-01 |

| ENTREZID | SYMBOL | GENENAME | FC | ADJ.PVAL |
| --- | --- | --- | --- | --- |

(Page generated on Tue Aug 25 20:50:55 2015 by ReportingTools 2.9.1 and hwriter 1.3.2)
