# Supplementary material for: Bioconductor’s EnrichmentBrowser: seamless navigation through combined results of set- & network-based enrichment analysis
Source: BMC Bioinformatics. 2016 Jan 20;17:45. doi: 10.1186/s12859-016-0884-1 (PMC4721010; doi:10.1186/s12859-016-0884-1)
Supplement: Supplementary file 2 — EnrichmentBrowser output (ALL microarray data). Unzip and open the contained index.html in the browser to view the contents of this file (tested with Firefox 39.0). (ZIP 2775 kb) [file 12859_2016_884_MOESM2_ESM.zip › ora.html]

ORA - Table of Results


## ORA - Table of Results

| GENE.SET | TITLE | NR.GENES | P.VALUE | SET.VIEW | PATH.VIEW |
| --- | --- | --- | --- | --- | --- |
| GENE.SET | TITLE | NR.GENES | P.VALUE | SET.VIEW | PATH.VIEW |
| hsa05416 | Viral myocarditis | 55 | 0.00330 |  |  |
| hsa04622 | RIG-I-like receptor signaling pathway | 54 | 0.00501 |  |  |
| hsa05130 | Pathogenic Escherichia coli infection | 43 | 0.01020 |  |  |
| hsa04520 | Adherens junction | 68 | 0.01150 |  |  |
| hsa05134 | Legionellosis | 48 | 0.01300 |  |  |

| GENE.SET | TITLE | NR.GENES | P.VALUE | SET.VIEW | PATH.VIEW |
| --- | --- | --- | --- | --- | --- |

(Page generated on Tue Aug 25 16:01:47 2015 by ReportingTools and hwriter )
