# Supplementary material for: Bioconductor’s EnrichmentBrowser: seamless navigation through combined results of set- & network-based enrichment analysis
Source: BMC Bioinformatics. 2016 Jan 20;17:45. doi: 10.1186/s12859-016-0884-1 (PMC4721010; doi:10.1186/s12859-016-0884-1)
Supplement: Supplementary file 3 — EnrichmentBrowser output (TCGA RNA-seq data). Unzip and open the contained index.html in the browser to view the contents of this file (tested with Firefox 39.0). (ZIP 7116.8 kb) [file 12859_2016_884_MOESM3_ESM.zip › comb.html]

COMB - Table of Results


## COMB - Table of Results

| GENE.SET | TITLE | NR.GENES | GSEA.RANK | PATHNET.RANK | NEA.RANK | SUM.RANK | GSEA.PVAL | PATHNET.PVAL | NEA.ZSCORE | SET.VIEW | PATH.VIEW | GRAPH.VIEW |
| --- | --- | --- | --- | --- | --- | --- | --- | --- | --- | --- | --- | --- |
| GENE.SET | TITLE | NR.GENES | GSEA.RANK | PATHNET.RANK | NEA.RANK | SUM.RANK | GSEA.PVAL | PATHNET.PVAL | NEA.ZSCORE | SET.VIEW | PATH.VIEW | GRAPH.VIEW |
| hsa04270 | Vascular smooth muscle contraction | 121 | 1.3 | 9.4 | 0.6 | 11.3 | 0.003 | 1.94e-03 | 2.02 |  |  |  |
| hsa04722 | Neurotrophin signaling pathway | 120 | 3.8 | 2.5 | 8.2 | 14.5 | 0.011 | 3.23e-04 | 1.89 |  |  |  |
| hsa05205 | Proteoglycans in cancer | 204 | 1.9 | 0.6 | 18.2 | 20.8 | 0.005 | 3.92e-05 | 1.76 |  |  |  |
| hsa04020 | Calcium signaling pathway | 180 | 6.3 | 15.1 | 6.3 | 27.7 | 0.016 | 6.92e-03 | 1.92 |  |  |  |
| hsa05200 | Pathways in cancer | 327 | 7.5 | 3.8 | 17.0 | 28.3 | 0.026 | 3.58e-04 | 1.78 |  |  |  |
| hsa04068 | FoxO signaling pathway | 132 | 10.1 | 17.0 | 3.8 | 30.8 | 0.036 | 7.41e-03 | 1.97 |  |  |  |
| hsa04115 | p53 signaling pathway | 67 | 2.5 | 16.4 | 24.5 | 43.4 | 0.006 | 7.40e-03 | 1.71 |  |  |  |
| hsa04150 | mTOR signaling pathway | 60 | 11.3 | 10.1 | 24.5 | 45.9 | 0.053 | 3.05e-03 | 1.71 |  |  |  |
| hsa05206 | MicroRNAs in cancer | 220 | 24.5 | 7.5 | 13.8 | 45.9 | 0.127 | 1.25e-03 | 1.80 |  |  |  |
| hsa05220 | Chronic myeloid leukemia | 73 | 8.8 | 1.9 | 38.4 | 49.1 | 0.029 | 1.76e-04 | 1.48 |  |  |  |

| GENE.SET | TITLE | NR.GENES | GSEA.RANK | PATHNET.RANK | NEA.RANK | SUM.RANK | GSEA.PVAL | PATHNET.PVAL | NEA.ZSCORE | SET.VIEW | PATH.VIEW | GRAPH.VIEW |
| --- | --- | --- | --- | --- | --- | --- | --- | --- | --- | --- | --- | --- |

(Page generated on Mon Aug 24 22:00:31 2015 by ReportingTools 2.9.1 and hwriter 1.3.2)
