# Supplementary material for: Bioconductor’s EnrichmentBrowser: seamless navigation through combined results of set- & network-based enrichment analysis
Source: BMC Bioinformatics. 2016 Jan 20;17:45. doi: 10.1186/s12859-016-0884-1 (PMC4721010; doi:10.1186/s12859-016-0884-1)
Supplement: Supplementary file 3 — EnrichmentBrowser output (TCGA RNA-seq data). Unzip and open the contained index.html in the browser to view the contents of this file (tested with Firefox 39.0). (ZIP 7116.8 kb) [file 12859_2016_884_MOESM3_ESM.zip › gsea.html]

GSEA - Table of Results


## GSEA - Table of Results

| GENE.SET | TITLE | NR.GENES | GLOB.STAT | NGLOB.STAT | P.VALUE | SET.VIEW | PATH.VIEW | GRAPH.VIEW |
| --- | --- | --- | --- | --- | --- | --- | --- | --- |
| GENE.SET | TITLE | NR.GENES | GLOB.STAT | NGLOB.STAT | P.VALUE | SET.VIEW | PATH.VIEW | GRAPH.VIEW |
| hsa04713 | Circadian entrainment | 96 | 134 | 1.400 | 0.002 |  |  |  |
| hsa04270 | Vascular smooth muscle contraction | 121 | 148 | 1.230 | 0.003 |  |  |  |
| hsa05205 | Proteoglycans in cancer | 204 | 130 | 0.638 | 0.005 |  |  |  |

| GENE.SET | TITLE | NR.GENES | GLOB.STAT | NGLOB.STAT | P.VALUE | SET.VIEW | PATH.VIEW | GRAPH.VIEW |
| --- | --- | --- | --- | --- | --- | --- | --- | --- |

(Page generated on Tue Aug 25 12:05:04 2015 by ReportingTools 2.9.1 and hwriter 1.3.2)
