# Supplementary material for: Bioconductor’s EnrichmentBrowser: seamless navigation through combined results of set- & network-based enrichment analysis
Source: BMC Bioinformatics. 2016 Jan 20;17:45. doi: 10.1186/s12859-016-0884-1 (PMC4721010; doi:10.1186/s12859-016-0884-1)
Supplement: Supplementary file 3 — EnrichmentBrowser output (TCGA RNA-seq data). Unzip and open the contained index.html in the browser to view the contents of this file (tested with Firefox 39.0). (ZIP 7116.8 kb) [file 12859_2016_884_MOESM3_ESM.zip › hsa04020.html]

hsa04020: Gene Report


## hsa04020: Gene Report

| ENTREZID | SYMBOL | GENENAME | FC | ADJ.PVAL |
| --- | --- | --- | --- | --- |
| ENTREZID | SYMBOL | GENENAME | FC | ADJ.PVAL |
| 10105 | PPIF | peptidylprolyl isomerase F | 1.55 | 1.5e-24 |
| 107 | ADCY1 | adenylate cyclase 1 (brain) | -0.73 | 2.1e-02 |
| 108 | ADCY2 | adenylate cyclase 2 (brain) | -4.06 | 2.1e-64 |
| 10800 | CYSLTR1 | cysteinyl leukotriene receptor 1 | -1.07 | 4.0e-05 |
| 109 | ADCY3 | adenylate cyclase 3 | -1.35 | 8.4e-24 |
| 1128 | CHRM1 | cholinergic receptor, muscarinic 1 | -0.91 | 2.7e-02 |
| 1129 | CHRM2 | cholinergic receptor, muscarinic 2 | -1.25 | 3.1e-03 |
| 113 | ADCY7 | adenylate cyclase 7 | 0.15 | 3.4e-01 |
| 113026 | PLCD3 | phospholipase C, delta 3 | -0.84 | 2.6e-05 |
| 1131 | CHRM3 | cholinergic receptor, muscarinic 3 | -2.24 | 2.7e-06 |
| 1133 | CHRM5 | cholinergic receptor, muscarinic 5 | -0.06 | 8.6e-01 |
| 1139 | CHRNA7 | cholinergic receptor, nicotinic, alpha 7 (neuronal) | -1.17 | 1.1e-04 |
| 114 | ADCY8 | adenylate cyclase 8 (brain) | -2.84 | 6.3e-10 |
| 115 | ADCY9 | adenylate cyclase 9 | -2.25 | 1.1e-33 |
| 135 | ADORA2A | adenosine A2a receptor | -0.50 | 2.1e-03 |
| 136 | ADORA2B | adenosine A2b receptor | 0.16 | 5.4e-01 |
| 146 | ADRA1D | adrenoceptor alpha 1D | -4.47 | 7.5e-45 |
| 147 | ADRA1B | adrenoceptor alpha 1B | -1.17 | 1.1e-03 |
| 148 | ADRA1A | adrenoceptor alpha 1A | -1.95 | 4.6e-09 |
| 153 | ADRB1 | adrenoceptor beta 1 | 0.11 | 7.7e-01 |
| 154 | ADRB2 | adrenoceptor beta 2, surface | -2.17 | 1.1e-15 |
| 155 | ADRB3 | adrenoceptor beta 3 | -4.15 | 5.3e-37 |
| 163688 | CALML6 | calmodulin-like 6 | 0.73 | 1.2e-02 |
| 1812 | DRD1 | dopamine receptor D1 | -1.89 | 9.1e-07 |
| 1816 | DRD5 | dopamine receptor D5 | 0.09 | 8.1e-01 |
| 185 | AGTR1 | angiotensin II receptor, type 1 | -3.96 | 1.2e-29 |
| 1909 | EDNRA | endothelin receptor type A | -3.16 | 1.3e-76 |
| 1910 | EDNRB | endothelin receptor type B | -2.74 | 1.6e-49 |
| 1956 | EGFR | epidermal growth factor receptor | -0.93 | 2.7e-06 |
| 196883 | ADCY4 | adenylate cyclase 4 | -1.78 | 1.3e-29 |
| 2064 | ERBB2 | erb-b2 receptor tyrosine kinase 2 | 0.85 | 3.8e-05 |
| 2065 | ERBB3 | erb-b2 receptor tyrosine kinase 3 | 2.14 | 2.7e-21 |
| 2066 | ERBB4 | erb-b2 receptor tyrosine kinase 4 | 1.88 | 9.6e-04 |
| 2149 | F2R | coagulation factor II (thrombin) receptor | -0.38 | 2.5e-02 |
| 2185 | PTK2B | protein tyrosine kinase 2 beta | -0.48 | 1.3e-03 |
| 22953 | P2RX2 | purinergic receptor P2X, ligand gated ion channel, 2 | -0.54 | 2.5e-01 |
| 23236 | PLCB1 | phospholipase C, beta 1 (phosphoinositide-specific) | 0.13 | 5.9e-01 |
| 2767 | GNA11 | guanine nucleotide binding protein (G protein), alpha 11 (Gq class) | -0.83 | 3.2e-07 |
| 2769 | GNA15 | guanine nucleotide binding protein (G protein), alpha 15 (Gq class) | 1.38 | 1.8e-08 |
| 2774 | GNAL | guanine nucleotide binding protein (G protein), alpha activating activity polypeptide, olfactory type | -2.58 | 7.1e-52 |
| 2776 | GNAQ | guanine nucleotide binding protein (G protein), q polypeptide | -0.64 | 8.9e-05 |
| 2778 | GNAS | GNAS complex locus | 0.76 | 5.9e-09 |
| 2902 | GRIN1 | glutamate receptor, ionotropic, N-methyl D-aspartate 1 | 3.06 | 2.6e-16 |
| 2903 | GRIN2A | glutamate receptor, ionotropic, N-methyl D-aspartate 2A | -3.44 | 2.8e-24 |
| 2905 | GRIN2C | glutamate receptor, ionotropic, N-methyl D-aspartate 2C | -0.12 | 7.4e-01 |
| 2906 | GRIN2D | glutamate receptor, ionotropic, N-methyl D-aspartate 2D | 3.73 | 1.3e-10 |
| 291 | SLC25A4 | solute carrier family 25 (mitochondrial carrier; adenine nucleotide translocator), member 4 | -0.02 | 8.9e-01 |
| 2911 | GRM1 | glutamate receptor, metabotropic 1 | -0.19 | 5.6e-01 |
| 2915 | GRM5 | glutamate receptor, metabotropic 5 | 0.32 | 4.7e-01 |
| 292 | SLC25A5 | solute carrier family 25 (mitochondrial carrier; adenine nucleotide translocator), member 5 | 0.87 | 4.8e-11 |
| 2925 | GRPR | gastrin-releasing peptide receptor | 0.99 | 4.9e-04 |
| 293 | SLC25A6 | solute carrier family 25 (mitochondrial carrier; adenine nucleotide translocator), member 6 | 0.01 | 9.7e-01 |
| 3269 | HRH1 | histamine receptor H1 | -0.96 | 1.5e-06 |
| 3274 | HRH2 | histamine receptor H2 | -0.89 | 8.8e-04 |
| 3356 | HTR2A | 5-hydroxytryptamine (serotonin) receptor 2A, G protein-coupled | -3.14 | 9.2e-20 |
| 3357 | HTR2B | 5-hydroxytryptamine (serotonin) receptor 2B, G protein-coupled | -2.69 | 1.2e-34 |
| 3358 | HTR2C | 5-hydroxytryptamine (serotonin) receptor 2C, G protein-coupled | 0.86 | 2.6e-02 |
| 3360 | HTR4 | 5-hydroxytryptamine (serotonin) receptor 4, G protein-coupled | -0.44 | 1.0e-01 |
| 3361 | HTR5A | 5-hydroxytryptamine (serotonin) receptor 5A, G protein-coupled | 0.43 | 5.4e-02 |
| 3362 | HTR6 | 5-hydroxytryptamine (serotonin) receptor 6, G protein-coupled | 1.21 | 1.5e-03 |
| 3363 | HTR7 | 5-hydroxytryptamine (serotonin) receptor 7, adenylate cyclase-coupled | -0.93 | 8.3e-04 |
| 340156 | MYLK4 | myosin light chain kinase family, member 4 | -0.58 | 6.6e-03 |
| 3706 | ITPKA | inositol-trisphosphate 3-kinase A | 0.37 | 2.1e-01 |
| 3707 | ITPKB | inositol-trisphosphate 3-kinase B | -1.51 | 7.5e-27 |
| 3708 | ITPR1 | inositol 1,4,5-trisphosphate receptor, type 1 | -2.91 | 1.2e-76 |
| 3709 | ITPR2 | inositol 1,4,5-trisphosphate receptor, type 2 | -0.73 | 2.6e-05 |
| 3710 | ITPR3 | inositol 1,4,5-trisphosphate receptor, type 3 | 1.58 | 3.7e-18 |
| 3973 | LHCGR | luteinizing hormone/choriogonadotropin receptor | -1.33 | 1.4e-08 |
| 4638 | MYLK | myosin light chain kinase | -4.35 | 3.0e-79 |
| 4842 | NOS1 | nitric oxide synthase 1 (neuronal) | -1.69 | 2.2e-05 |
| 4843 | NOS2 | nitric oxide synthase 2, inducible | -1.14 | 4.1e-04 |
| 4846 | NOS3 | nitric oxide synthase 3 (endothelial cell) | -0.71 | 6.4e-05 |
| 487 | ATP2A1 | ATPase, Ca++ transporting, cardiac muscle, fast twitch 1 | 1.07 | 1.3e-07 |
| 488 | ATP2A2 | ATPase, Ca++ transporting, cardiac muscle, slow twitch 2 | 0.37 | 4.4e-04 |
| 489 | ATP2A3 | ATPase, Ca++ transporting, ubiquitous | 0.25 | 3.8e-01 |
| 490 | ATP2B1 | ATPase, Ca++ transporting, plasma membrane 1 | -0.39 | 3.5e-03 |
| 491 | ATP2B2 | ATPase, Ca++ transporting, plasma membrane 2 | -0.95 | 2.2e-02 |
| 492 | ATP2B3 | ATPase, Ca++ transporting, plasma membrane 3 | -0.45 | 3.6e-01 |
| 4923 | NTSR1 | neurotensin receptor 1 (high affinity) | 1.32 | 2.1e-04 |
| 493 | ATP2B4 | ATPase, Ca++ transporting, plasma membrane 4 | -1.39 | 3.5e-18 |
| 5021 | OXTR | oxytocin receptor | -0.33 | 1.7e-01 |
| 5023 | P2RX1 | purinergic receptor P2X, ligand gated ion channel, 1 | -2.02 | 1.5e-14 |
| 5024 | P2RX3 | purinergic receptor P2X, ligand gated ion channel, 3 | 0.19 | 5.9e-01 |
| 5025 | P2RX4 | purinergic receptor P2X, ligand gated ion channel, 4 | 0.96 | 3.1e-08 |
| 5026 | P2RX5 | purinergic receptor P2X, ligand gated ion channel, 5 | 1.62 | 1.3e-07 |
| 5027 | P2RX7 | purinergic receptor P2X, ligand gated ion channel, 7 | -2.05 | 1.4e-26 |
| 51196 | PLCE1 | phospholipase C, epsilon 1 | -1.01 | 2.2e-06 |
| 5136 | PDE1A | phosphodiesterase 1A, calmodulin-dependent | -2.21 | 3.8e-36 |
| 5137 | PDE1C | phosphodiesterase 1C, calmodulin-dependent 70kDa | -2.93 | 1.1e-21 |
| 5153 | PDE1B | phosphodiesterase 1B, calmodulin-dependent | -2.43 | 5.1e-45 |
| 5156 | PDGFRA | platelet-derived growth factor receptor, alpha polypeptide | -3.00 | 7.1e-35 |
| 5159 | PDGFRB | platelet-derived growth factor receptor, beta polypeptide | -1.85 | 6.8e-23 |
| 51806 | CALML5 | calmodulin-like 5 | 2.63 | 2.8e-08 |
| 5255 | PHKA1 | phosphorylase kinase, alpha 1 (muscle) | 0.33 | 1.8e-02 |
| 5256 | PHKA2 | phosphorylase kinase, alpha 2 (liver) | -0.30 | 3.8e-03 |
| 5257 | PHKB | phosphorylase kinase, beta | -0.21 | 7.7e-02 |
| 5260 | PHKG1 | phosphorylase kinase, gamma 1 (muscle) | -0.44 | 3.4e-03 |
| 5261 | PHKG2 | phosphorylase kinase, gamma 2 (testis) | 1.07 | 4.2e-17 |
| 5330 | PLCB2 | phospholipase C, beta 2 | 0.12 | 5.6e-01 |
| 5331 | PLCB3 | phospholipase C, beta 3 (phosphatidylinositol-specific) | 0.28 | 1.9e-02 |
| 5332 | PLCB4 | phospholipase C, beta 4 | 0.18 | 4.4e-01 |
| 5333 | PLCD1 | phospholipase C, delta 1 | -0.77 | 1.3e-11 |
| 5335 | PLCG1 | phospholipase C, gamma 1 | -0.02 | 9.1e-01 |
| 5336 | PLCG2 | phospholipase C, gamma 2 (phosphatidylinositol-specific) | -0.70 | 1.7e-05 |
| 5350 | PLN | phospholamban | -6.00 | 1.1e-113 |
| 552 | AVPR1A | arginine vasopressin receptor 1A | -3.68 | 1.1e-40 |
| 553 | AVPR1B | arginine vasopressin receptor 1B | 2.12 | 7.8e-08 |
| 5530 | PPP3CA | protein phosphatase 3, catalytic subunit, alpha isozyme | -0.56 | 6.7e-06 |
| 5532 | PPP3CB | protein phosphatase 3, catalytic subunit, beta isozyme | -1.07 | 4.0e-32 |
| 5533 | PPP3CC | protein phosphatase 3, catalytic subunit, gamma isozyme | -0.72 | 7.4e-08 |
| 5534 | PPP3R1 | protein phosphatase 3, regulatory subunit B, alpha | -0.25 | 1.5e-02 |
| 5535 | PPP3R2 | protein phosphatase 3, regulatory subunit B, beta | 0.24 | 2.3e-01 |
| 5566 | PRKACA | protein kinase, cAMP-dependent, catalytic, alpha | -0.17 | 1.0e-01 |
| 5567 | PRKACB | protein kinase, cAMP-dependent, catalytic, beta | -1.29 | 1.2e-22 |
| 5568 | PRKACG | protein kinase, cAMP-dependent, catalytic, gamma | 0.05 | 8.3e-01 |
| 5578 | PRKCA | protein kinase C, alpha | -1.99 | 8.5e-30 |
| 5579 | PRKCB | protein kinase C, beta | -1.76 | 5.6e-18 |
| 5582 | PRKCG | protein kinase C, gamma | 0.28 | 5.7e-01 |
| 5613 | PRKX | protein kinase, X-linked | 0.96 | 1.9e-08 |
| 56413 | LTB4R2 | leukotriene B4 receptor 2 | 0.00 | 1.0e+00 |
| 56848 | SPHK2 | sphingosine kinase 2 | 0.68 | 3.3e-08 |
| 57105 | CYSLTR2 | cysteinyl leukotriene receptor 2 | -2.47 | 1.3e-15 |
| 5724 | PTAFR | platelet-activating factor receptor | 0.95 | 2.8e-04 |
| 5731 | PTGER1 | prostaglandin E receptor 1 (subtype EP1), 42kDa | 0.64 | 7.6e-02 |
| 5733 | PTGER3 | prostaglandin E receptor 3 (subtype EP3) | -5.59 | 3.2e-82 |
| 5737 | PTGFR | prostaglandin F receptor (FP) | -6.14 | 5.8e-98 |
| 57620 | STIM2 | stromal interaction molecule 2 | -0.41 | 9.1e-06 |
| 623 | BDKRB1 | bradykinin receptor B1 | -0.61 | 3.4e-02 |
| 624 | BDKRB2 | bradykinin receptor B2 | -0.98 | 1.1e-07 |
| 6261 | RYR1 | ryanodine receptor 1 (skeletal) | 0.39 | 2.9e-01 |
| 6262 | RYR2 | ryanodine receptor 2 (cardiac) | -1.76 | 2.3e-16 |
| 6263 | RYR3 | ryanodine receptor 3 | -3.67 | 2.3e-98 |
| 6543 | SLC8A2 | solute carrier family 8 (sodium/calcium exchanger), member 2 | -3.58 | 1.1e-42 |
| 6546 | SLC8A1 | solute carrier family 8 (sodium/calcium exchanger), member 1 | -2.41 | 1.3e-35 |
| 6547 | SLC8A3 | solute carrier family 8 (sodium/calcium exchanger), member 3 | -1.14 | 4.8e-03 |
| 6786 | STIM1 | stromal interaction molecule 1 | -0.55 | 8.0e-06 |
| 6865 | TACR2 | tachykinin receptor 2 | -2.84 | 3.4e-50 |
| 6869 | TACR1 | tachykinin receptor 1 | -2.79 | 1.3e-23 |
| 6870 | TACR3 | tachykinin receptor 3 | 0.47 | 4.8e-02 |
| 6915 | TBXA2R | thromboxane A2 receptor | -1.65 | 1.0e-26 |
| 7125 | TNNC2 | troponin C type 2 (fast) | -0.58 | 5.6e-02 |
| 7134 | TNNC1 | troponin C type 1 (slow) | 2.36 | 2.9e-06 |
| 7201 | TRHR | thyrotropin-releasing hormone receptor | 0.61 | 6.1e-04 |
| 7416 | VDAC1 | voltage-dependent anion channel 1 | 1.13 | 2.3e-28 |
| 7417 | VDAC2 | voltage-dependent anion channel 2 | 0.01 | 9.2e-01 |
| 7419 | VDAC3 | voltage-dependent anion channel 3 | 0.50 | 8.8e-07 |
| 773 | CACNA1A | calcium channel, voltage-dependent, P/Q type, alpha 1A subunit | -0.58 | 3.9e-02 |
| 774 | CACNA1B | calcium channel, voltage-dependent, N type, alpha 1B subunit | -0.30 | 5.3e-01 |
| 775 | CACNA1C | calcium channel, voltage-dependent, L type, alpha 1C subunit | -2.86 | 5.9e-54 |
| 776 | CACNA1D | calcium channel, voltage-dependent, L type, alpha 1D subunit | -1.31 | 1.4e-07 |
| 777 | CACNA1E | calcium channel, voltage-dependent, R type, alpha 1E subunit | 0.92 | 2.5e-02 |
| 778 | CACNA1F | calcium channel, voltage-dependent, L type, alpha 1F subunit | -0.33 | 2.4e-01 |
| 779 | CACNA1S | calcium channel, voltage-dependent, L type, alpha 1S subunit | 0.94 | 4.2e-02 |
| 801 | CALM1 | calmodulin 1 (phosphorylase kinase, delta) | 0.18 | 5.9e-02 |
| 80228 | ORAI2 | ORAI calcium release-activated calcium modulator 2 | 0.39 | 2.2e-03 |
| 80271 | ITPKC | inositol-trisphosphate 3-kinase C | 0.08 | 5.0e-01 |
| 805 | CALM2 | calmodulin 2 (phosphorylase kinase, delta) | -0.31 | 3.5e-04 |
| 808 | CALM3 | calmodulin 3 (phosphorylase kinase, delta) | -0.07 | 5.5e-01 |
| 810 | CALML3 | calmodulin-like 3 | 2.15 | 5.2e-04 |
| 814 | CAMK4 | calcium/calmodulin-dependent protein kinase IV | -0.76 | 7.5e-03 |
| 815 | CAMK2A | calcium/calmodulin-dependent protein kinase II alpha | -4.90 | 5.4e-81 |
| 816 | CAMK2B | calcium/calmodulin-dependent protein kinase II beta | 1.05 | 1.4e-02 |
| 817 | CAMK2D | calcium/calmodulin-dependent protein kinase II delta | -0.96 | 7.9e-11 |
| 818 | CAMK2G | calcium/calmodulin-dependent protein kinase II gamma | -0.99 | 4.9e-27 |
| 83447 | SLC25A31 | solute carrier family 25 (mitochondrial carrier; adenine nucleotide translocator), member 31 | 0.47 | 3.2e-02 |
| 84812 | PLCD4 | phospholipase C, delta 4 | -0.47 | 1.2e-04 |
| 84876 | ORAI1 | ORAI calcium release-activated calcium modulator 1 | 0.68 | 3.3e-05 |
| 85366 | MYLK2 | myosin light chain kinase 2 | 1.75 | 2.1e-14 |
| 886 | CCKAR | cholecystokinin A receptor | -0.34 | 2.5e-01 |
| 887 | CCKBR | cholecystokinin B receptor | 0.04 | 9.4e-01 |
| 8877 | SPHK1 | sphingosine kinase 1 | -0.17 | 4.4e-01 |
| 8911 | CACNA1I | calcium channel, voltage-dependent, T type, alpha 1I subunit | 0.82 | 4.6e-02 |
| 8912 | CACNA1H | calcium channel, voltage-dependent, T type, alpha 1H subunit | -4.11 | 6.8e-100 |
| 8913 | CACNA1G | calcium channel, voltage-dependent, T type, alpha 1G subunit | -3.17 | 3.0e-37 |
| 89869 | PLCZ1 | phospholipase C, zeta 1 | -0.05 | 8.7e-01 |
| 9127 | P2RX6 | purinergic receptor P2X, ligand gated ion channel, 6 | -1.61 | 2.5e-08 |
| 91807 | MYLK3 | myosin light chain kinase 3 | -0.55 | 1.1e-01 |
| 93129 | ORAI3 | ORAI calcium release-activated calcium modulator 3 | -0.14 | 3.0e-01 |
| 952 | CD38 | CD38 molecule | 0.85 | 1.7e-02 |
| 9630 | GNA14 | guanine nucleotide binding protein (G protein), alpha 14 | -2.53 | 1.0e-49 |

| ENTREZID | SYMBOL | GENENAME | FC | ADJ.PVAL |
| --- | --- | --- | --- | --- |

(Page generated on Mon Aug 24 22:02:29 2015 by ReportingTools 2.9.1 and hwriter 1.3.2)
