# Supplementary material for: Bioconductor’s EnrichmentBrowser: seamless navigation through combined results of set- & network-based enrichment analysis
Source: BMC Bioinformatics. 2016 Jan 20;17:45. doi: 10.1186/s12859-016-0884-1 (PMC4721010; doi:10.1186/s12859-016-0884-1)
Supplement: Supplementary file 3 — EnrichmentBrowser output (TCGA RNA-seq data). Unzip and open the contained index.html in the browser to view the contents of this file (tested with Firefox 39.0). (ZIP 7116.8 kb) [file 12859_2016_884_MOESM3_ESM.zip › hsa04068.html]

hsa04068: Gene Report


## hsa04068: Gene Report

| ENTREZID | SYMBOL | GENENAME | FC | ADJ.PVAL |
| --- | --- | --- | --- | --- |
| ENTREZID | SYMBOL | GENENAME | FC | ADJ.PVAL |
| 10000 | AKT3 | v-akt murine thymoma viral oncogene homolog 3 | -3.49 | 6.4e-108 |
| 10018 | BCL2L11 | BCL2-like 11 (apoptosis facilitator) | 0.97 | 2.1e-13 |
| 100533105 | C8orf44-SGK3 | C8orf44-SGK3 readthrough | -0.35 | 8.1e-02 |
| 10110 | SGK2 | serum/glucocorticoid regulated kinase 2 | 1.27 | 1.7e-03 |
| 1017 | CDK2 | cyclin-dependent kinase 2 | 0.55 | 2.3e-05 |
| 1026 | CDKN1A | cyclin-dependent kinase inhibitor 1A (p21, Cip1) | 0.08 | 7.6e-01 |
| 1027 | CDKN1B | cyclin-dependent kinase inhibitor 1B (p27, Kip1) | -1.42 | 1.6e-15 |
| 1030 | CDKN2B | cyclin-dependent kinase inhibitor 2B (p15, inhibits CDK4) | 1.13 | 5.8e-05 |
| 1032 | CDKN2D | cyclin-dependent kinase inhibitor 2D (p19, inhibits CDK4) | 0.64 | 3.0e-04 |
| 10365 | KLF2 | Kruppel-like factor 2 | -3.34 | 8.7e-91 |
| 10733 | PLK4 | polo-like kinase 4 | 2.16 | 4.6e-23 |
| 10769 | PLK2 | polo-like kinase 2 | -0.57 | 1.0e-02 |
| 10912 | GADD45G | growth arrest and DNA-damage-inducible, gamma | -1.60 | 1.2e-15 |
| 11337 | GABARAP | GABA(A) receptor-associated protein | 0.05 | 7.0e-01 |
| 11345 | GABARAPL2 | GABA(A) receptor-associated protein-like 2 | -0.72 | 4.6e-17 |
| 1147 | CHUK | conserved helix-loop-helix ubiquitous kinase | 0.13 | 1.9e-01 |
| 114907 | FBXO32 | F-box protein 32 | -3.47 | 1.8e-75 |
| 116986 | AGAP2 | ArfGAP with GTPase domain, ankyrin repeat and PH domain 2 | -0.19 | 3.2e-01 |
| 1263 | PLK3 | polo-like kinase 3 | -0.63 | 5.9e-08 |
| 1387 | CREBBP | CREB binding protein | -0.60 | 2.6e-08 |
| 1432 | MAPK14 | mitogen-activated protein kinase 14 | -0.56 | 2.9e-13 |
| 1454 | CSNK1E | casein kinase 1, epsilon | -0.10 | 3.6e-01 |
| 1647 | GADD45A | growth arrest and DNA-damage-inducible, alpha | -0.45 | 6.4e-03 |
| 1901 | S1PR1 | sphingosine-1-phosphate receptor 1 | -2.05 | 7.5e-46 |
| 1950 | EGF | epidermal growth factor | 0.15 | 7.1e-01 |
| 1956 | EGFR | epidermal growth factor receptor | -0.93 | 2.7e-06 |
| 2033 | EP300 | E1A binding protein p300 | -0.20 | 1.2e-01 |
| 207 | AKT1 | v-akt murine thymoma viral oncogene homolog 1 | 0.54 | 4.8e-09 |
| 208 | AKT2 | v-akt murine thymoma viral oncogene homolog 2 | 0.08 | 5.2e-01 |
| 2290 | FOXG1 | forkhead box G1 | 4.24 | 1.1e-14 |
| 2308 | FOXO1 | forkhead box O1 | -1.67 | 4.7e-33 |
| 2309 | FOXO3 | forkhead box O3 | -0.42 | 2.4e-05 |
| 23411 | SIRT1 | sirtuin 1 | -0.96 | 8.5e-18 |
| 23533 | PIK3R5 | phosphoinositide-3-kinase, regulatory subunit 5 | 0.24 | 2.6e-01 |
| 23678 | SGK3 | serum/glucocorticoid regulated kinase family, member 3 | 0.26 | 3.4e-01 |
| 23710 | GABARAPL1 | GABA(A) receptor-associated protein like 1 | -1.31 | 1.5e-16 |
| 2538 | G6PC | glucose-6-phosphatase, catalytic subunit | 0.26 | 4.1e-01 |
| 26260 | FBXO25 | F-box protein 25 | 0.19 | 9.1e-02 |
| 2885 | GRB2 | growth factor receptor-bound protein 2 | 0.00 | 9.9e-01 |
| 2911 | GRM1 | glutamate receptor, metabotropic 1 | -0.19 | 5.6e-01 |
| 3265 | HRAS | Harvey rat sarcoma viral oncogene homolog | 0.90 | 2.8e-08 |
| 3276 | PRMT1 | protein arginine methyltransferase 1 | 0.65 | 2.3e-06 |
| 3479 | IGF1 | insulin-like growth factor 1 (somatomedin C) | -2.10 | 3.2e-15 |
| 3480 | IGF1R | insulin-like growth factor 1 receptor | -0.97 | 2.1e-05 |
| 3551 | IKBKB | inhibitor of kappa light polypeptide gene enhancer in B-cells, kinase beta | -0.27 | 2.9e-03 |
| 356 | FASLG | Fas ligand (TNF superfamily, member 6) | -0.18 | 6.0e-01 |
| 3569 | IL6 | interleukin 6 | -0.95 | 5.1e-03 |
| 3575 | IL7R | interleukin 7 receptor | 0.10 | 7.8e-01 |
| 3586 | IL10 | interleukin 10 | 1.03 | 8.8e-04 |
| 3630 | INS | insulin | 0.08 | 6.2e-01 |
| 3643 | INSR | insulin receptor | -1.47 | 6.1e-25 |
| 3667 | IRS1 | insulin receptor substrate 1 | -2.16 | 2.8e-24 |
| 369 | ARAF | A-Raf proto-oncogene, serine/threonine kinase | -0.12 | 2.8e-01 |
| 3845 | KRAS | Kirsten rat sarcoma viral oncogene homolog | 0.33 | 1.3e-02 |
| 4087 | SMAD2 | SMAD family member 2 | -0.18 | 4.8e-02 |
| 4088 | SMAD3 | SMAD family member 3 | -1.19 | 3.7e-21 |
| 4089 | SMAD4 | SMAD family member 4 | -0.86 | 4.9e-19 |
| 4193 | MDM2 | MDM2 proto-oncogene, E3 ubiquitin protein ligase | 0.32 | 3.8e-02 |
| 4303 | FOXO4 | forkhead box O4 | -1.55 | 9.6e-50 |
| 4616 | GADD45B | growth arrest and DNA-damage-inducible, beta | -0.82 | 1.4e-05 |
| 472 | ATM | ATM serine/threonine kinase | -0.93 | 2.3e-14 |
| 4893 | NRAS | neuroblastoma RAS viral (v-ras) oncogene homolog | 0.76 | 2.6e-07 |
| 5105 | PCK1 | phosphoenolpyruvate carboxykinase 1 (soluble) | -0.64 | 2.7e-01 |
| 5106 | PCK2 | phosphoenolpyruvate carboxykinase 2 (mitochondrial) | 1.48 | 4.7e-15 |
| 51422 | PRKAG2 | protein kinase, AMP-activated, gamma 2 non-catalytic subunit | -0.13 | 3.3e-01 |
| 5170 | PDPK1 | 3-phosphoinositide dependent protein kinase 1 | -0.68 | 8.0e-12 |
| 51701 | NLK | nemo-like kinase | 0.47 | 3.1e-06 |
| 5290 | PIK3CA | phosphatidylinositol-4,5-bisphosphate 3-kinase, catalytic subunit alpha | -0.34 | 1.2e-02 |
| 5291 | PIK3CB | phosphatidylinositol-4,5-bisphosphate 3-kinase, catalytic subunit beta | 0.19 | 8.7e-02 |
| 5293 | PIK3CD | phosphatidylinositol-4,5-bisphosphate 3-kinase, catalytic subunit delta | -0.71 | 1.6e-06 |
| 5294 | PIK3CG | phosphatidylinositol-4,5-bisphosphate 3-kinase, catalytic subunit gamma | -0.98 | 4.5e-05 |
| 5295 | PIK3R1 | phosphoinositide-3-kinase, regulatory subunit 1 (alpha) | -0.80 | 2.1e-03 |
| 5296 | PIK3R2 | phosphoinositide-3-kinase, regulatory subunit 2 (beta) | 0.67 | 6.5e-08 |
| 5347 | PLK1 | polo-like kinase 1 | 3.96 | 1.4e-32 |
| 53632 | PRKAG3 | protein kinase, AMP-activated, gamma 3 non-catalytic subunit | -0.24 | 4.7e-01 |
| 5562 | PRKAA1 | protein kinase, AMP-activated, alpha 1 catalytic subunit | -0.79 | 1.2e-10 |
| 5563 | PRKAA2 | protein kinase, AMP-activated, alpha 2 catalytic subunit | -0.64 | 3.1e-03 |
| 5564 | PRKAB1 | protein kinase, AMP-activated, beta 1 non-catalytic subunit | 0.33 | 9.2e-03 |
| 5565 | PRKAB2 | protein kinase, AMP-activated, beta 2 non-catalytic subunit | -0.82 | 2.7e-11 |
| 5571 | PRKAG1 | protein kinase, AMP-activated, gamma 1 non-catalytic subunit | 0.15 | 5.4e-02 |
| 5594 | MAPK1 | mitogen-activated protein kinase 1 | -0.24 | 2.6e-02 |
| 5595 | MAPK3 | mitogen-activated protein kinase 3 | -0.86 | 1.2e-13 |
| 5599 | MAPK8 | mitogen-activated protein kinase 8 | 0.06 | 7.3e-01 |
| 5600 | MAPK11 | mitogen-activated protein kinase 11 | -1.30 | 3.5e-16 |
| 5601 | MAPK9 | mitogen-activated protein kinase 9 | -0.12 | 1.0e-01 |
| 5602 | MAPK10 | mitogen-activated protein kinase 10 | -2.79 | 1.3e-41 |
| 5603 | MAPK13 | mitogen-activated protein kinase 13 | 2.49 | 3.5e-23 |
| 5604 | MAP2K1 | mitogen-activated protein kinase kinase 1 | -0.05 | 6.3e-01 |
| 5605 | MAP2K2 | mitogen-activated protein kinase kinase 2 | 0.76 | 2.3e-06 |
| 5728 | PTEN | phosphatase and tensin homolog | -0.86 | 7.4e-09 |
| 57818 | G6PC2 | glucose-6-phosphatase, catalytic, 2 | -0.43 | 4.5e-02 |
| 5894 | RAF1 | Raf-1 proto-oncogene, serine/threonine kinase | -0.11 | 1.2e-01 |
| 5896 | RAG1 | recombination activating gene 1 | -0.71 | 3.2e-03 |
| 5897 | RAG2 | recombination activating gene 2 | 0.16 | 5.6e-01 |
| 5934 | RBL2 | retinoblastoma-like 2 | -1.12 | 1.4e-20 |
| 595 | CCND1 | cyclin D1 | 0.84 | 4.4e-04 |
| 604 | BCL6 | B-cell CLL/lymphoma 6 | -0.82 | 4.7e-10 |
| 6300 | MAPK12 | mitogen-activated protein kinase 12 | -0.73 | 1.2e-03 |
| 6446 | SGK1 | serum/glucocorticoid regulated kinase 1 | -1.04 | 1.2e-08 |
| 6502 | SKP2 | S-phase kinase-associated protein 2, E3 ubiquitin protein ligase | 1.04 | 3.9e-10 |
| 6517 | SLC2A4 | solute carrier family 2 (facilitated glucose transporter), member 4 | -3.97 | 4.0e-85 |
| 664 | BNIP3 | BCL2/adenovirus E1B 19kDa interacting protein 3 | 1.11 | 4.3e-07 |
| 6648 | SOD2 | superoxide dismutase 2, mitochondrial | 0.44 | 3.6e-02 |
| 6654 | SOS1 | son of sevenless homolog 1 (Drosophila) | -0.44 | 1.5e-06 |
| 6655 | SOS2 | son of sevenless homolog 2 (Drosophila) | -0.83 | 1.9e-19 |
| 673 | BRAF | B-Raf proto-oncogene, serine/threonine kinase | 0.82 | 1.7e-09 |
| 6774 | STAT3 | signal transducer and activator of transcription 3 (acute-phase response factor) | -0.02 | 8.3e-01 |
| 6789 | STK4 | serine/threonine kinase 4 | 0.07 | 4.8e-01 |
| 6794 | STK11 | serine/threonine kinase 11 | 0.12 | 3.0e-01 |
| 7040 | TGFB1 | transforming growth factor, beta 1 | -0.65 | 7.3e-05 |
| 7042 | TGFB2 | transforming growth factor, beta 2 | -1.33 | 1.4e-08 |
| 7043 | TGFB3 | transforming growth factor, beta 3 | -1.52 | 1.4e-25 |
| 7046 | TGFBR1 | transforming growth factor, beta receptor 1 | -0.62 | 1.0e-09 |
| 7048 | TGFBR2 | transforming growth factor, beta receptor II (70/80kDa) | -1.75 | 6.2e-30 |
| 7874 | USP7 | ubiquitin specific peptidase 7 (herpes virus-associated) | 0.11 | 1.3e-01 |
| 80854 | SETD7 | SET domain containing (lysine methyltransferase) 7 | -2.38 | 2.7e-45 |
| 847 | CAT | catalase | -0.58 | 3.3e-05 |
| 8471 | IRS4 | insulin receptor substrate 4 | -4.11 | 4.0e-32 |
| 8503 | PIK3R3 | phosphoinositide-3-kinase, regulatory subunit 3 (gamma) | 1.28 | 1.7e-10 |
| 85417 | CCNB3 | cyclin B3 | 1.24 | 3.0e-07 |
| 8660 | IRS2 | insulin receptor substrate 2 | -2.11 | 3.7e-22 |
| 8698 | S1PR4 | sphingosine-1-phosphate receptor 4 | -0.01 | 9.7e-01 |
| 8743 | TNFSF10 | tumor necrosis factor (ligand) superfamily, member 10 | -0.19 | 4.7e-01 |
| 891 | CCNB1 | cyclin B1 | 3.13 | 1.8e-37 |
| 894 | CCND2 | cyclin D2 | -2.90 | 1.3e-37 |
| 901 | CCNG2 | cyclin G2 | -0.28 | 3.4e-02 |
| 9133 | CCNB2 | cyclin B2 | 4.19 | 5.5e-41 |
| 9140 | ATG12 | autophagy related 12 | -0.34 | 3.0e-04 |
| 92579 | G6PC3 | glucose 6 phosphatase, catalytic, 3 | 0.73 | 1.3e-07 |
| 9454 | HOMER3 | homer scaffolding protein 3 | 0.87 | 2.6e-06 |
| 9455 | HOMER2 | homer scaffolding protein 2 | 2.48 | 5.5e-10 |
| 9456 | HOMER1 | homer scaffolding protein 1 | -0.09 | 6.9e-01 |

| ENTREZID | SYMBOL | GENENAME | FC | ADJ.PVAL |
| --- | --- | --- | --- | --- |

(Page generated on Tue Aug 25 12:04:49 2015 by ReportingTools 2.9.1 and hwriter 1.3.2)
