# Supplementary material for: Bioconductor’s EnrichmentBrowser: seamless navigation through combined results of set- & network-based enrichment analysis
Source: BMC Bioinformatics. 2016 Jan 20;17:45. doi: 10.1186/s12859-016-0884-1 (PMC4721010; doi:10.1186/s12859-016-0884-1)
Supplement: Supplementary file 3 — EnrichmentBrowser output (TCGA RNA-seq data). Unzip and open the contained index.html in the browser to view the contents of this file (tested with Firefox 39.0). (ZIP 7116.8 kb) [file 12859_2016_884_MOESM3_ESM.zip › hsa04080.html]

hsa04080: Gene Report


## hsa04080: Gene Report

| ENTREZID | SYMBOL | GENENAME | FC | ADJ.PVAL |
| --- | --- | --- | --- | --- |
| ENTREZID | SYMBOL | GENENAME | FC | ADJ.PVAL |
| 10161 | LPAR6 | lysophosphatidic acid receptor 6 | -1.64 | 3.5e-27 |
| 10203 | CALCRL | calcitonin receptor-like | -2.17 | 6.2e-33 |
| 10316 | NMUR1 | neuromedin U receptor 1 | -2.14 | 6.7e-21 |
| 10800 | CYSLTR1 | cysteinyl leukotriene receptor 1 | -1.07 | 4.0e-05 |
| 1081 | CGA | glycoprotein hormones, alpha polypeptide | 1.47 | 1.4e-04 |
| 10886 | NPFFR2 | neuropeptide FF receptor 2 | -0.16 | 7.2e-01 |
| 10888 | GPR83 | G protein-coupled receptor 83 | 1.40 | 4.8e-04 |
| 11255 | HRH3 | histamine receptor H3 | 1.35 | 1.2e-05 |
| 1128 | CHRM1 | cholinergic receptor, muscarinic 1 | -0.91 | 2.7e-02 |
| 1129 | CHRM2 | cholinergic receptor, muscarinic 2 | -1.25 | 3.1e-03 |
| 1131 | CHRM3 | cholinergic receptor, muscarinic 3 | -2.24 | 2.7e-06 |
| 1132 | CHRM4 | cholinergic receptor, muscarinic 4 | 0.37 | 3.0e-01 |
| 1133 | CHRM5 | cholinergic receptor, muscarinic 5 | -0.06 | 8.6e-01 |
| 1134 | CHRNA1 | cholinergic receptor, nicotinic, alpha 1 (muscle) | 3.88 | 4.1e-32 |
| 1135 | CHRNA2 | cholinergic receptor, nicotinic, alpha 2 (neuronal) | -0.51 | 5.1e-02 |
| 1136 | CHRNA3 | cholinergic receptor, nicotinic, alpha 3 (neuronal) | -0.05 | 9.1e-01 |
| 1137 | CHRNA4 | cholinergic receptor, nicotinic, alpha 4 (neuronal) | -1.67 | 3.0e-04 |
| 1138 | CHRNA5 | cholinergic receptor, nicotinic, alpha 5 (neuronal) | 1.41 | 1.4e-05 |
| 1139 | CHRNA7 | cholinergic receptor, nicotinic, alpha 7 (neuronal) | -1.17 | 1.1e-04 |
| 1140 | CHRNB1 | cholinergic receptor, nicotinic, beta 1 (muscle) | 0.09 | 5.7e-01 |
| 1141 | CHRNB2 | cholinergic receptor, nicotinic, beta 2 (neuronal) | 0.37 | 2.3e-01 |
| 1142 | CHRNB3 | cholinergic receptor, nicotinic, beta 3 (neuronal) | -0.05 | 8.3e-01 |
| 1143 | CHRNB4 | cholinergic receptor, nicotinic, beta 4 (neuronal) | -0.57 | 1.2e-01 |
| 1144 | CHRND | cholinergic receptor, nicotinic, delta (muscle) | 1.62 | 5.8e-07 |
| 1145 | CHRNE | cholinergic receptor, nicotinic, epsilon (muscle) | -0.68 | 2.7e-03 |
| 1146 | CHRNG | cholinergic receptor, nicotinic, gamma (muscle) | 1.63 | 1.3e-09 |
| 116443 | GRIN3A | glutamate receptor, ionotropic, N-methyl-D-aspartate 3A | -0.80 | 6.3e-05 |
| 116444 | GRIN3B | glutamate receptor, ionotropic, N-methyl-D-aspartate 3B | 0.07 | 8.9e-01 |
| 117 | ADCYAP1R1 | adenylate cyclase activating polypeptide 1 (pituitary) receptor type I | -2.14 | 1.9e-08 |
| 122042 | RXFP2 | relaxin/insulin-like family peptide receptor 2 | -1.59 | 6.9e-09 |
| 1241 | LTB4R | leukotriene B4 receptor | 0.28 | 1.6e-01 |
| 1268 | CNR1 | cannabinoid receptor 1 (brain) | -2.50 | 7.3e-12 |
| 1269 | CNR2 | cannabinoid receptor 2 (macrophage) | 0.30 | 3.1e-01 |
| 134 | ADORA1 | adenosine A1 receptor | 0.85 | 1.0e-02 |
| 134860 | TAAR9 | trace amine associated receptor 9 (gene/pseudogene) | 0.54 | 1.9e-04 |
| 134864 | TAAR1 | trace amine associated receptor 1 | -1.11 | 4.3e-06 |
| 135 | ADORA2A | adenosine A2a receptor | -0.50 | 2.1e-03 |
| 136 | ADORA2B | adenosine A2b receptor | 0.16 | 5.4e-01 |
| 1394 | CRHR1 | corticotropin releasing hormone receptor 1 | -3.61 | 3.8e-19 |
| 1395 | CRHR2 | corticotropin releasing hormone receptor 2 | -1.90 | 7.6e-09 |
| 140 | ADORA3 | adenosine A3 receptor | 0.14 | 5.5e-01 |
| 1442 | CSH1 | chorionic somatomammotropin hormone 1 (placental lactogen) | 0.47 | 2.2e-03 |
| 1443 | CSH2 | chorionic somatomammotropin hormone 2 | 0.79 | 5.0e-05 |
| 146 | ADRA1D | adrenoceptor alpha 1D | -4.47 | 7.5e-45 |
| 147 | ADRA1B | adrenoceptor alpha 1B | -1.17 | 1.1e-03 |
| 148 | ADRA1A | adrenoceptor alpha 1A | -1.95 | 4.6e-09 |
| 150 | ADRA2A | adrenoceptor alpha 2A | -1.41 | 7.5e-07 |
| 151 | ADRA2B | adrenoceptor alpha 2B | -1.02 | 4.2e-04 |
| 1511 | CTSG | cathepsin G | -3.87 | 1.4e-23 |
| 152 | ADRA2C | adrenoceptor alpha 2C | -1.71 | 1.8e-11 |
| 153 | ADRB1 | adrenoceptor beta 1 | 0.11 | 7.7e-01 |
| 154 | ADRB2 | adrenoceptor beta 2, surface | -2.17 | 1.1e-15 |
| 155 | ADRB3 | adrenoceptor beta 3 | -4.15 | 5.3e-37 |
| 165829 | GPR156 | G protein-coupled receptor 156 | -0.57 | 6.5e-02 |
| 1812 | DRD1 | dopamine receptor D1 | -1.89 | 9.1e-07 |
| 1813 | DRD2 | dopamine receptor D2 | -2.46 | 2.1e-11 |
| 1814 | DRD3 | dopamine receptor D3 | 0.39 | 7.3e-02 |
| 1815 | DRD4 | dopamine receptor D4 | -0.41 | 1.0e-01 |
| 1816 | DRD5 | dopamine receptor D5 | 0.09 | 8.1e-01 |
| 185 | AGTR1 | angiotensin II receptor, type 1 | -3.96 | 1.2e-29 |
| 186 | AGTR2 | angiotensin II receptor, type 2 | -2.40 | 1.3e-04 |
| 187 | APLNR | apelin receptor | -0.67 | 2.5e-03 |
| 1901 | S1PR1 | sphingosine-1-phosphate receptor 1 | -2.05 | 7.5e-46 |
| 1902 | LPAR1 | lysophosphatidic acid receptor 1 | -1.28 | 1.1e-11 |
| 1903 | S1PR3 | sphingosine-1-phosphate receptor 3 | -1.84 | 5.4e-35 |
| 1909 | EDNRA | endothelin receptor type A | -3.16 | 1.3e-76 |
| 1910 | EDNRB | endothelin receptor type B | -2.74 | 1.6e-49 |
| 200959 | GABRR3 | gamma-aminobutyric acid (GABA) A receptor, rho 3 (gene/pseudogene) | 0.28 | 6.4e-02 |
| 2147 | F2 | coagulation factor II (thrombin) | 1.23 | 1.3e-03 |
| 2149 | F2R | coagulation factor II (thrombin) receptor | -0.38 | 2.5e-02 |
| 2150 | F2RL1 | coagulation factor II (thrombin) receptor-like 1 | 2.54 | 1.3e-14 |
| 2151 | F2RL2 | coagulation factor II (thrombin) receptor-like 2 | -0.12 | 7.3e-01 |
| 22953 | P2RX2 | purinergic receptor P2X, ligand gated ion channel, 2 | -0.54 | 2.5e-01 |
| 23566 | LPAR3 | lysophosphatidic acid receptor 3 | 0.97 | 8.0e-02 |
| 2357 | FPR1 | formyl peptide receptor 1 | 1.08 | 3.5e-04 |
| 2358 | FPR2 | formyl peptide receptor 2 | 1.26 | 1.1e-04 |
| 2359 | FPR3 | formyl peptide receptor 3 | 0.79 | 2.5e-03 |
| 23620 | NTSR2 | neurotensin receptor 2 | -0.47 | 1.3e-01 |
| 2488 | FSHB | follicle stimulating hormone, beta polypeptide | 0.50 | 1.2e-03 |
| 2492 | FSHR | follicle stimulating hormone receptor | -0.70 | 2.5e-03 |
| 2550 | GABBR1 | gamma-aminobutyric acid (GABA) B receptor, 1 | -2.15 | 1.1e-23 |
| 2554 | GABRA1 | gamma-aminobutyric acid (GABA) A receptor, alpha 1 | 0.47 | 3.8e-02 |
| 2555 | GABRA2 | gamma-aminobutyric acid (GABA) A receptor, alpha 2 | -1.08 | 1.3e-02 |
| 2556 | GABRA3 | gamma-aminobutyric acid (GABA) A receptor, alpha 3 | 0.71 | 1.6e-01 |
| 2557 | GABRA4 | gamma-aminobutyric acid (GABA) A receptor, alpha 4 | -0.25 | 3.2e-01 |
| 2558 | GABRA5 | gamma-aminobutyric acid (GABA) A receptor, alpha 5 | 0.68 | 3.1e-02 |
| 2559 | GABRA6 | gamma-aminobutyric acid (GABA) A receptor, alpha 6 | 0.04 | 8.3e-01 |
| 2560 | GABRB1 | gamma-aminobutyric acid (GABA) A receptor, beta 1 | -0.83 | 6.9e-03 |
| 2561 | GABRB2 | gamma-aminobutyric acid (GABA) A receptor, beta 2 | -1.51 | 2.1e-04 |
| 2562 | GABRB3 | gamma-aminobutyric acid (GABA) A receptor, beta 3 | -3.37 | 7.1e-31 |
| 2563 | GABRD | gamma-aminobutyric acid (GABA) A receptor, delta | 1.33 | 1.6e-08 |
| 2564 | GABRE | gamma-aminobutyric acid (GABA) A receptor, epsilon | -1.46 | 1.3e-06 |
| 2565 | GABRG1 | gamma-aminobutyric acid (GABA) A receptor, gamma 1 | -2.42 | 1.1e-08 |
| 2566 | GABRG2 | gamma-aminobutyric acid (GABA) A receptor, gamma 2 | 0.33 | 2.7e-01 |
| 2567 | GABRG3 | gamma-aminobutyric acid (GABA) A receptor, gamma 3 | 0.97 | 3.6e-02 |
| 2568 | GABRP | gamma-aminobutyric acid (GABA) A receptor, pi | -0.06 | 9.2e-01 |
| 2569 | GABRR1 | gamma-aminobutyric acid (GABA) A receptor, rho 1 | 1.16 | 5.1e-04 |
| 2570 | GABRR2 | gamma-aminobutyric acid (GABA) A receptor, rho 2 | -0.11 | 7.3e-01 |
| 2587 | GALR1 | galanin receptor 1 | -0.77 | 2.3e-02 |
| 2642 | GCGR | glucagon receptor | 1.55 | 6.9e-04 |
| 2688 | GH1 | growth hormone 1 | -1.13 | 1.3e-04 |
| 2689 | GH2 | growth hormone 2 | 0.53 | 1.7e-02 |
| 2690 | GHR | growth hormone receptor | -3.69 | 1.7e-73 |
| 2692 | GHRHR | growth hormone releasing hormone receptor | 0.19 | 4.3e-01 |
| 2693 | GHSR | growth hormone secretagogue receptor | 0.86 | 1.7e-04 |
| 2696 | GIPR | gastric inhibitory polypeptide receptor | 0.35 | 3.2e-01 |
| 27334 | P2RY10 | purinergic receptor P2Y, G-protein coupled, 10 | -0.06 | 8.7e-01 |
| 2740 | GLP1R | glucagon-like peptide 1 receptor | 1.49 | 8.1e-05 |
| 2741 | GLRA1 | glycine receptor, alpha 1 | 0.38 | 1.3e-01 |
| 2742 | GLRA2 | glycine receptor, alpha 2 | 0.03 | 9.5e-01 |
| 2743 | GLRB | glycine receptor, beta | -0.20 | 4.3e-01 |
| 2798 | GNRHR | gonadotropin-releasing hormone receptor | 0.18 | 5.6e-01 |
| 2831 | NPBWR1 | neuropeptides B/W receptor 1 | 0.90 | 6.2e-05 |
| 2832 | NPBWR2 | neuropeptides B/W receptor 2 | 0.49 | 4.3e-03 |
| 2834 | PRLHR | prolactin releasing hormone receptor | -4.76 | 6.7e-31 |
| 2837 | UTS2R | urotensin 2 receptor | 1.04 | 2.4e-03 |
| 2846 | LPAR4 | lysophosphatidic acid receptor 4 | -2.29 | 5.8e-14 |
| 2847 | MCHR1 | melanin-concentrating hormone receptor 1 | -2.36 | 2.5e-13 |
| 2859 | GPR35 | G protein-coupled receptor 35 | 1.42 | 4.9e-05 |
| 2862 | MLNR | motilin receptor | 1.30 | 7.6e-06 |
| 286530 | P2RY8 | purinergic receptor P2Y, G-protein coupled, 8 | -0.59 | 3.2e-02 |
| 2890 | GRIA1 | glutamate receptor, ionotropic, AMPA 1 | -2.86 | 8.5e-13 |
| 2891 | GRIA2 | glutamate receptor, ionotropic, AMPA 2 | -1.48 | 2.0e-03 |
| 2892 | GRIA3 | glutamate receptor, ionotropic, AMPA 3 | -3.62 | 9.0e-33 |
| 2893 | GRIA4 | glutamate receptor, ionotropic, AMPA 4 | -1.25 | 1.4e-03 |
| 2894 | GRID1 | glutamate receptor, ionotropic, delta 1 | -2.77 | 1.2e-54 |
| 2895 | GRID2 | glutamate receptor, ionotropic, delta 2 | -1.43 | 9.0e-04 |
| 2897 | GRIK1 | glutamate receptor, ionotropic, kainate 1 | -0.58 | 6.1e-02 |
| 2898 | GRIK2 | glutamate receptor, ionotropic, kainate 2 | 0.54 | 2.1e-01 |
| 2899 | GRIK3 | glutamate receptor, ionotropic, kainate 3 | 0.89 | 3.3e-02 |
| 2900 | GRIK4 | glutamate receptor, ionotropic, kainate 4 | 0.20 | 6.1e-01 |
| 2901 | GRIK5 | glutamate receptor, ionotropic, kainate 5 | -2.19 | 1.8e-15 |
| 2902 | GRIN1 | glutamate receptor, ionotropic, N-methyl D-aspartate 1 | 3.06 | 2.6e-16 |
| 2903 | GRIN2A | glutamate receptor, ionotropic, N-methyl D-aspartate 2A | -3.44 | 2.8e-24 |
| 2904 | GRIN2B | glutamate receptor, ionotropic, N-methyl D-aspartate 2B | 0.88 | 6.8e-03 |
| 2905 | GRIN2C | glutamate receptor, ionotropic, N-methyl D-aspartate 2C | -0.12 | 7.4e-01 |
| 2906 | GRIN2D | glutamate receptor, ionotropic, N-methyl D-aspartate 2D | 3.73 | 1.3e-10 |
| 2908 | NR3C1 | nuclear receptor subfamily 3, group C, member 1 (glucocorticoid receptor) | -2.71 | 2.9e-62 |
| 2911 | GRM1 | glutamate receptor, metabotropic 1 | -0.19 | 5.6e-01 |
| 2912 | GRM2 | glutamate receptor, metabotropic 2 | 0.97 | 3.5e-04 |
| 2913 | GRM3 | glutamate receptor, metabotropic 3 | -0.53 | 1.8e-01 |
| 2914 | GRM4 | glutamate receptor, metabotropic 4 | 0.66 | 6.5e-02 |
| 2915 | GRM5 | glutamate receptor, metabotropic 5 | 0.32 | 4.7e-01 |
| 2916 | GRM6 | glutamate receptor, metabotropic 6 | -1.49 | 4.2e-06 |
| 2917 | GRM7 | glutamate receptor, metabotropic 7 | -2.40 | 6.9e-10 |
| 2918 | GRM8 | glutamate receptor, metabotropic 8 | 2.04 | 5.3e-09 |
| 2925 | GRPR | gastrin-releasing peptide receptor | 0.99 | 4.9e-04 |
| 3001 | GZMA | granzyme A (granzyme 1, cytotoxic T-lymphocyte-associated serine esterase 3) | -0.30 | 3.2e-01 |
| 3061 | HCRTR1 | hypocretin (orexin) receptor 1 | -0.41 | 1.4e-01 |
| 3062 | HCRTR2 | hypocretin (orexin) receptor 2 | -0.56 | 8.4e-02 |
| 319100 | TAAR6 | trace amine associated receptor 6 | 0.67 | 3.0e-04 |
| 3269 | HRH1 | histamine receptor H1 | -0.96 | 1.5e-06 |
| 3274 | HRH2 | histamine receptor H2 | -0.89 | 8.8e-04 |
| 3350 | HTR1A | 5-hydroxytryptamine (serotonin) receptor 1A, G protein-coupled | 0.52 | 2.1e-02 |
| 3351 | HTR1B | 5-hydroxytryptamine (serotonin) receptor 1B, G protein-coupled | -1.16 | 4.2e-05 |
| 3352 | HTR1D | 5-hydroxytryptamine (serotonin) receptor 1D, G protein-coupled | 0.89 | 5.0e-03 |
| 3354 | HTR1E | 5-hydroxytryptamine (serotonin) receptor 1E, G protein-coupled | -2.79 | 8.7e-09 |
| 3355 | HTR1F | 5-hydroxytryptamine (serotonin) receptor 1F, G protein-coupled | -0.11 | 7.2e-01 |
| 3356 | HTR2A | 5-hydroxytryptamine (serotonin) receptor 2A, G protein-coupled | -3.14 | 9.2e-20 |
| 3357 | HTR2B | 5-hydroxytryptamine (serotonin) receptor 2B, G protein-coupled | -2.69 | 1.2e-34 |
| 3358 | HTR2C | 5-hydroxytryptamine (serotonin) receptor 2C, G protein-coupled | 0.86 | 2.6e-02 |
| 3360 | HTR4 | 5-hydroxytryptamine (serotonin) receptor 4, G protein-coupled | -0.44 | 1.0e-01 |
| 3361 | HTR5A | 5-hydroxytryptamine (serotonin) receptor 5A, G protein-coupled | 0.43 | 5.4e-02 |
| 3362 | HTR6 | 5-hydroxytryptamine (serotonin) receptor 6, G protein-coupled | 1.21 | 1.5e-03 |
| 3363 | HTR7 | 5-hydroxytryptamine (serotonin) receptor 7, adenylate cyclase-coupled | -0.93 | 8.3e-04 |
| 3952 | LEP | leptin | 0.16 | 6.2e-01 |
| 3953 | LEPR | leptin receptor | -3.31 | 3.8e-80 |
| 3972 | LHB | luteinizing hormone beta polypeptide | 1.64 | 3.9e-09 |
| 3973 | LHCGR | luteinizing hormone/choriogonadotropin receptor | -1.33 | 1.4e-08 |
| 4142 | MAS1 | MAS1 proto-oncogene, G protein-coupled receptor | -2.03 | 2.9e-24 |
| 4157 | MC1R | melanocortin 1 receptor (alpha melanocyte stimulating hormone receptor) | 0.46 | 4.0e-02 |
| 4158 | MC2R | melanocortin 2 receptor (adrenocorticotropic hormone) | 0.51 | 3.2e-03 |
| 4159 | MC3R | melanocortin 3 receptor | 0.31 | 2.6e-01 |
| 4160 | MC4R | melanocortin 4 receptor | -0.94 | 4.0e-02 |
| 4161 | MC5R | melanocortin 5 receptor | 0.85 | 8.5e-06 |
| 4543 | MTNR1A | melatonin receptor 1A | 0.32 | 3.1e-01 |
| 4544 | MTNR1B | melatonin receptor 1B | 0.51 | 2.2e-02 |
| 4829 | NMBR | neuromedin B receptor | -1.33 | 9.2e-07 |
| 4886 | NPY1R | neuropeptide Y receptor Y1 | -3.52 | 5.0e-32 |
| 4887 | NPY2R | neuropeptide Y receptor Y2 | 0.39 | 2.3e-01 |
| 4889 | NPY5R | neuropeptide Y receptor Y5 | -3.39 | 6.8e-22 |
| 4923 | NTSR1 | neurotensin receptor 1 (high affinity) | 1.32 | 2.1e-04 |
| 4985 | OPRD1 | opioid receptor, delta 1 | 0.89 | 5.0e-04 |
| 4986 | OPRK1 | opioid receptor, kappa 1 | 2.98 | 1.5e-10 |
| 4987 | OPRL1 | opiate receptor-like 1 | -0.10 | 6.6e-01 |
| 4988 | OPRM1 | opioid receptor, mu 1 | 0.55 | 5.0e-02 |
| 5021 | OXTR | oxytocin receptor | -0.33 | 1.7e-01 |
| 5023 | P2RX1 | purinergic receptor P2X, ligand gated ion channel, 1 | -2.02 | 1.5e-14 |
| 5024 | P2RX3 | purinergic receptor P2X, ligand gated ion channel, 3 | 0.19 | 5.9e-01 |
| 5025 | P2RX4 | purinergic receptor P2X, ligand gated ion channel, 4 | 0.96 | 3.1e-08 |
| 5026 | P2RX5 | purinergic receptor P2X, ligand gated ion channel, 5 | 1.62 | 1.3e-07 |
| 5027 | P2RX7 | purinergic receptor P2X, ligand gated ion channel, 7 | -2.05 | 1.4e-26 |
| 5028 | P2RY1 | purinergic receptor P2Y, G-protein coupled, 1 | -2.20 | 1.2e-21 |
| 5029 | P2RY2 | purinergic receptor P2Y, G-protein coupled, 2 | 1.53 | 9.7e-08 |
| 5030 | P2RY4 | pyrimidinergic receptor P2Y, G-protein coupled, 4 | -0.10 | 6.8e-01 |
| 5031 | P2RY6 | pyrimidinergic receptor P2Y, G-protein coupled, 6 | 1.45 | 3.7e-08 |
| 5032 | P2RY11 | purinergic receptor P2Y, G-protein coupled, 11 | 0.19 | 2.0e-01 |
| 5340 | PLG | plasminogen | -1.24 | 3.5e-03 |
| 53637 | S1PR5 | sphingosine-1-phosphate receptor 5 | 0.53 | 7.4e-02 |
| 53829 | P2RY13 | purinergic receptor P2Y, G-protein coupled, 13 | -1.15 | 6.7e-06 |
| 552 | AVPR1A | arginine vasopressin receptor 1A | -3.68 | 1.1e-40 |
| 553 | AVPR1B | arginine vasopressin receptor 1B | 2.12 | 7.8e-08 |
| 554 | AVPR2 | arginine vasopressin receptor 2 | -2.95 | 1.3e-23 |
| 5540 | NPY4R | neuropeptide Y receptor Y4 | 0.26 | 4.1e-01 |
| 55584 | CHRNA9 | cholinergic receptor, nicotinic, alpha 9 (neuronal) | 1.54 | 1.9e-05 |
| 55879 | GABRQ | gamma-aminobutyric acid (GABA) A receptor, theta | -0.97 | 3.9e-02 |
| 5617 | PRL | prolactin | -0.71 | 7.4e-02 |
| 5618 | PRLR | prolactin receptor | -2.20 | 1.3e-17 |
| 56288 | PARD3 | par-3 family cell polarity regulator | -0.28 | 6.2e-03 |
| 56413 | LTB4R2 | leukotriene B4 receptor 2 | 0.00 | 1.0e+00 |
| 5644 | PRSS1 | protease, serine, 1 (trypsin 1) | 4.51 | 1.1e-21 |
| 5646 | PRSS3 | protease, serine, 3 | 2.32 | 2.4e-10 |
| 56923 | NMUR2 | neuromedin U receptor 2 | 1.76 | 2.2e-06 |
| 57053 | CHRNA10 | cholinergic receptor, nicotinic, alpha 10 (neuronal) | 0.41 | 3.0e-02 |
| 57105 | CYSLTR2 | cysteinyl leukotriene receptor 2 | -2.47 | 1.3e-15 |
| 5724 | PTAFR | platelet-activating factor receptor | 0.95 | 2.8e-04 |
| 5729 | PTGDR | prostaglandin D2 receptor (DP) | -2.30 | 2.5e-19 |
| 5731 | PTGER1 | prostaglandin E receptor 1 (subtype EP1), 42kDa | 0.64 | 7.6e-02 |
| 5732 | PTGER2 | prostaglandin E receptor 2 (subtype EP2), 53kDa | -2.01 | 4.6e-24 |
| 5733 | PTGER3 | prostaglandin E receptor 3 (subtype EP3) | -5.59 | 3.2e-82 |
| 5734 | PTGER4 | prostaglandin E receptor 4 (subtype EP4) | -0.99 | 5.2e-09 |
| 5737 | PTGFR | prostaglandin F receptor (FP) | -6.14 | 5.8e-98 |
| 5739 | PTGIR | prostaglandin I2 (prostacyclin) receptor (IP) | -0.31 | 1.0e-01 |
| 5745 | PTH1R | parathyroid hormone 1 receptor | -2.91 | 4.7e-38 |
| 5746 | PTH2R | parathyroid hormone 2 receptor | 0.53 | 4.1e-01 |
| 59340 | HRH4 | histamine receptor H4 | -0.62 | 1.5e-02 |
| 59350 | RXFP1 | relaxin/insulin-like family peptide receptor 1 | -0.39 | 3.4e-01 |
| 623 | BDKRB1 | bradykinin receptor B1 | -0.61 | 3.4e-02 |
| 624 | BDKRB2 | bradykinin receptor B2 | -0.98 | 1.1e-07 |
| 6344 | SCTR | secretin receptor | 0.92 | 4.4e-02 |
| 64106 | NPFFR1 | neuropeptide FF receptor 1 | 0.88 | 3.2e-03 |
| 6751 | SSTR1 | somatostatin receptor 1 | -2.83 | 2.9e-16 |
| 6752 | SSTR2 | somatostatin receptor 2 | 0.58 | 5.5e-02 |
| 6753 | SSTR3 | somatostatin receptor 3 | -2.34 | 1.1e-10 |
| 6754 | SSTR4 | somatostatin receptor 4 | -0.58 | 4.6e-03 |
| 6755 | SSTR5 | somatostatin receptor 5 | -0.17 | 7.2e-01 |
| 680 | BRS3 | bombesin-like receptor 3 | -0.89 | 1.8e-03 |
| 6865 | TACR2 | tachykinin receptor 2 | -2.84 | 3.4e-50 |
| 6869 | TACR1 | tachykinin receptor 1 | -2.79 | 1.3e-23 |
| 6870 | TACR3 | tachykinin receptor 3 | 0.47 | 4.8e-02 |
| 6915 | TBXA2R | thromboxane A2 receptor | -1.65 | 1.0e-26 |
| 706 | TSPO | translocator protein (18kDa) | 0.72 | 5.1e-05 |
| 7067 | THRA | thyroid hormone receptor, alpha | -1.87 | 3.6e-33 |
| 7068 | THRB | thyroid hormone receptor, beta | -2.12 | 2.0e-15 |
| 719 | C3AR1 | complement component 3a receptor 1 | -0.08 | 7.3e-01 |
| 7201 | TRHR | thyrotropin-releasing hormone receptor | 0.61 | 6.1e-04 |
| 7252 | TSHB | thyroid stimulating hormone, beta | -2.58 | 8.3e-33 |
| 7253 | TSHR | thyroid stimulating hormone receptor | -1.18 | 8.5e-03 |
| 728 | C5AR1 | complement component 5a receptor 1 | 0.15 | 4.8e-01 |
| 7433 | VIPR1 | vasoactive intestinal peptide receptor 1 | 1.00 | 1.5e-03 |
| 7434 | VIPR2 | vasoactive intestinal peptide receptor 2 | -2.76 | 6.9e-30 |
| 7442 | TRPV1 | transient receptor potential cation channel, subfamily V, member 1 | -0.05 | 7.1e-01 |
| 799 | CALCR | calcitonin receptor | 0.98 | 8.0e-04 |
| 8001 | GLRA3 | glycine receptor, alpha 3 | -0.47 | 7.4e-02 |
| 83551 | TAAR8 | trace amine associated receptor 8 | 0.48 | 7.8e-03 |
| 84539 | MCHR2 | melanin-concentrating hormone receptor 2 | 0.25 | 3.3e-01 |
| 84634 | KISS1R | KISS1 receptor | 3.35 | 1.5e-19 |
| 8484 | GALR3 | galanin receptor 3 | 1.40 | 1.1e-07 |
| 8698 | S1PR4 | sphingosine-1-phosphate receptor 4 | -0.01 | 9.7e-01 |
| 8811 | GALR2 | galanin receptor 2 | 2.89 | 1.6e-24 |
| 886 | CCKAR | cholecystokinin A receptor | -0.34 | 2.5e-01 |
| 887 | CCKBR | cholecystokinin B receptor | 0.04 | 9.4e-01 |
| 8973 | CHRNA6 | cholinergic receptor, nicotinic, alpha 6 (neuronal) | -0.20 | 5.6e-01 |
| 9002 | F2RL3 | coagulation factor II (thrombin) receptor-like 3 | 0.90 | 2.6e-05 |
| 9038 | TAAR5 | trace amine associated receptor 5 | 0.35 | 1.5e-01 |
| 9127 | P2RX6 | purinergic receptor P2X, ligand gated ion channel, 6 | -1.61 | 2.5e-08 |
| 9170 | LPAR2 | lysophosphatidic acid receptor 2 | 2.12 | 2.4e-23 |
| 9248 | GPR50 | G protein-coupled receptor 50 | -0.21 | 6.3e-01 |
| 9294 | S1PR2 | sphingosine-1-phosphate receptor 2 | 0.41 | 9.4e-03 |
| 9340 | GLP2R | glucagon-like peptide 2 receptor | -1.96 | 5.5e-10 |
| 9568 | GABBR2 | gamma-aminobutyric acid (GABA) B receptor, 2 | 0.15 | 7.0e-01 |
| 9934 | P2RY14 | purinergic receptor P2Y, G-protein coupled, 14 | -2.11 | 3.8e-16 |

| ENTREZID | SYMBOL | GENENAME | FC | ADJ.PVAL |
| --- | --- | --- | --- | --- |

(Page generated on Tue Aug 25 12:04:49 2015 by ReportingTools 2.9.1 and hwriter 1.3.2)
