# Supplementary material for: Bioconductor’s EnrichmentBrowser: seamless navigation through combined results of set- & network-based enrichment analysis
Source: BMC Bioinformatics. 2016 Jan 20;17:45. doi: 10.1186/s12859-016-0884-1 (PMC4721010; doi:10.1186/s12859-016-0884-1)
Supplement: Supplementary file 3 — EnrichmentBrowser output (TCGA RNA-seq data). Unzip and open the contained index.html in the browser to view the contents of this file (tested with Firefox 39.0). (ZIP 7116.8 kb) [file 12859_2016_884_MOESM3_ESM.zip › hsa04115.html]

hsa04115: Gene Report


## hsa04115: Gene Report

| ENTREZID | SYMBOL | GENENAME | FC | ADJ.PVAL |
| --- | --- | --- | --- | --- |
| ENTREZID | SYMBOL | GENENAME | FC | ADJ.PVAL |
| 1017 | CDK2 | cyclin-dependent kinase 2 | 0.55 | 2.3e-05 |
| 1019 | CDK4 | cyclin-dependent kinase 4 | 0.28 | 8.6e-03 |
| 1021 | CDK6 | cyclin-dependent kinase 6 | -0.93 | 6.5e-06 |
| 1026 | CDKN1A | cyclin-dependent kinase inhibitor 1A (p21, Cip1) | 0.08 | 7.6e-01 |
| 1029 | CDKN2A | cyclin-dependent kinase inhibitor 2A | 3.96 | 1.9e-09 |
| 10912 | GADD45G | growth arrest and DNA-damage-inducible, gamma | -1.60 | 1.2e-15 |
| 1111 | CHEK1 | checkpoint kinase 1 | 1.71 | 1.4e-17 |
| 11200 | CHEK2 | checkpoint kinase 2 | 1.34 | 7.9e-17 |
| 143686 | SESN3 | sestrin 3 | 0.10 | 6.4e-01 |
| 1643 | DDB2 | damage-specific DNA binding protein 2, 48kDa | -0.29 | 3.2e-02 |
| 1647 | GADD45A | growth arrest and DNA-damage-inducible, alpha | -0.45 | 6.4e-03 |
| 25898 | RCHY1 | ring finger and CHY zinc finger domain containing 1, E3 ubiquitin protein ligase | -0.37 | 5.1e-05 |
| 27113 | BBC3 | BCL2 binding component 3 | 0.58 | 1.6e-03 |
| 27244 | SESN1 | sestrin 1 | -1.19 | 9.4e-25 |
| 2810 | SFN | stratifin | 5.06 | 2.8e-15 |
| 317 | APAF1 | apoptotic peptidase activating factor 1 | -0.22 | 1.4e-01 |
| 3479 | IGF1 | insulin-like growth factor 1 (somatomedin C) | -2.10 | 3.2e-15 |
| 3486 | IGFBP3 | insulin-like growth factor binding protein 3 | -0.41 | 7.1e-02 |
| 355 | FAS | Fas cell surface death receptor | -1.02 | 8.0e-12 |
| 3732 | CD82 | CD82 molecule | 0.71 | 2.9e-03 |
| 4193 | MDM2 | MDM2 proto-oncogene, E3 ubiquitin protein ligase | 0.32 | 3.8e-02 |
| 4194 | MDM4 | MDM4, p53 regulator | -0.10 | 4.3e-01 |
| 4616 | GADD45B | growth arrest and DNA-damage-inducible, beta | -0.82 | 1.4e-05 |
| 472 | ATM | ATM serine/threonine kinase | -0.93 | 2.3e-14 |
| 50484 | RRM2B | ribonucleotide reductase M2 B (TP53 inducible) | -0.10 | 4.9e-01 |
| 5054 | SERPINE1 | serpin peptidase inhibitor, clade E (nexin, plasminogen activator inhibitor type 1), member 1 | -0.41 | 9.3e-02 |
| 51246 | SHISA5 | shisa family member 5 | 0.48 | 6.0e-05 |
| 51512 | GTSE1 | G-2 and S-phase expressed 1 | 4.17 | 3.2e-41 |
| 5268 | SERPINB5 | serpin peptidase inhibitor, clade B (ovalbumin), member 5 | 2.74 | 7.2e-08 |
| 5366 | PMAIP1 | phorbol-12-myristate-13-acetate-induced protein 1 | 0.94 | 6.2e-04 |
| 54205 | CYCS | cytochrome c, somatic | 0.47 | 1.4e-03 |
| 545 | ATR | ATR serine/threonine kinase | 0.42 | 9.9e-05 |
| 55240 | STEAP3 | STEAP family member 3, metalloreductase | 1.03 | 5.7e-05 |
| 56475 | RPRM | reprimo, TP53 dependent G2 arrest mediator candidate | -4.80 | 5.8e-65 |
| 5728 | PTEN | phosphatase and tensin homolog | -0.86 | 7.4e-09 |
| 575 | ADGRB1 | adhesion G protein-coupled receptor B1 | -2.15 | 9.7e-14 |
| 581 | BAX | BCL2-associated X protein | 0.89 | 9.4e-11 |
| 595 | CCND1 | cyclin D1 | 0.84 | 4.4e-04 |
| 6241 | RRM2 | ribonucleotide reductase M2 | 4.62 | 1.0e-32 |
| 637 | BID | BH3 interacting domain death agonist | 0.92 | 2.4e-14 |
| 63970 | TP53AIP1 | tumor protein p53 regulated apoptosis inducing protein 1 | 1.44 | 6.9e-04 |
| 64065 | PERP | PERP, TP53 apoptosis effector | 1.05 | 3.4e-08 |
| 64326 | RFWD2 | ring finger and WD repeat domain 2, E3 ubiquitin protein ligase | 0.70 | 1.0e-17 |
| 64393 | ZMAT3 | zinc finger, matrin-type 3 | -0.78 | 4.2e-10 |
| 6477 | SIAH1 | siah E3 ubiquitin protein ligase 1 | -0.74 | 1.6e-13 |
| 7057 | THBS1 | thrombospondin 1 | -2.40 | 1.1e-22 |
| 7157 | TP53 | tumor protein p53 | 0.53 | 2.8e-03 |
| 7161 | TP73 | tumor protein p73 | 1.55 | 1.2e-04 |
| 7249 | TSC2 | tuberous sclerosis 2 | 0.35 | 2.2e-03 |
| 836 | CASP3 | caspase 3, apoptosis-related cysteine peptidase | 0.88 | 6.8e-18 |
| 83667 | SESN2 | sestrin 2 | 0.45 | 1.0e-03 |
| 841 | CASP8 | caspase 8, apoptosis-related cysteine peptidase | 0.30 | 1.2e-02 |
| 842 | CASP9 | caspase 9, apoptosis-related cysteine peptidase | -0.14 | 1.2e-01 |
| 8493 | PPM1D | protein phosphatase, Mg2+/Mn2+ dependent, 1D | -0.71 | 1.1e-17 |
| 85417 | CCNB3 | cyclin B3 | 1.24 | 3.0e-07 |
| 8795 | TNFRSF10B | tumor necrosis factor receptor superfamily, member 10b | 0.82 | 7.3e-08 |
| 891 | CCNB1 | cyclin B1 | 3.13 | 1.8e-37 |
| 894 | CCND2 | cyclin D2 | -2.90 | 1.3e-37 |
| 896 | CCND3 | cyclin D3 | -0.38 | 5.4e-04 |
| 898 | CCNE1 | cyclin E1 | 3.74 | 4.9e-19 |
| 900 | CCNG1 | cyclin G1 | -0.67 | 7.8e-07 |
| 901 | CCNG2 | cyclin G2 | -0.28 | 3.4e-02 |
| 9133 | CCNB2 | cyclin B2 | 4.19 | 5.5e-41 |
| 9134 | CCNE2 | cyclin E2 | 2.01 | 6.1e-12 |
| 9538 | EI24 | etoposide induced 2.4 | 0.35 | 3.5e-04 |
| 9540 | TP53I3 | tumor protein p53 inducible protein 3 | 1.26 | 2.5e-13 |
| 983 | CDK1 | cyclin-dependent kinase 1 | 2.97 | 2.5e-23 |

| ENTREZID | SYMBOL | GENENAME | FC | ADJ.PVAL |
| --- | --- | --- | --- | --- |

(Page generated on Mon Aug 24 22:02:12 2015 by ReportingTools 2.9.1 and hwriter 1.3.2)
