# Supplementary material for: Bioconductor’s EnrichmentBrowser: seamless navigation through combined results of set- & network-based enrichment analysis
Source: BMC Bioinformatics. 2016 Jan 20;17:45. doi: 10.1186/s12859-016-0884-1 (PMC4721010; doi:10.1186/s12859-016-0884-1)
Supplement: Supplementary file 3 — EnrichmentBrowser output (TCGA RNA-seq data). Unzip and open the contained index.html in the browser to view the contents of this file (tested with Firefox 39.0). (ZIP 7116.8 kb) [file 12859_2016_884_MOESM3_ESM.zip › hsa04150.html]

hsa04150: Gene Report


## hsa04150: Gene Report

| ENTREZID | SYMBOL | GENENAME | FC | ADJ.PVAL |
| --- | --- | --- | --- | --- |
| ENTREZID | SYMBOL | GENENAME | FC | ADJ.PVAL |
| 10000 | AKT3 | v-akt murine thymoma viral oncogene homolog 3 | -3.49 | 6.4e-108 |
| 10325 | RRAGB | Ras-related GTP binding B | -1.05 | 2.3e-23 |
| 10670 | RRAGA | Ras-related GTP binding A | -0.85 | 2.2e-10 |
| 1975 | EIF4B | eukaryotic translation initiation factor 4B | -1.06 | 1.3e-23 |
| 1977 | EIF4E | eukaryotic translation initiation factor 4E | 0.08 | 5.0e-01 |
| 1978 | EIF4EBP1 | eukaryotic translation initiation factor 4E binding protein 1 | 1.82 | 1.3e-15 |
| 207 | AKT1 | v-akt murine thymoma viral oncogene homolog 1 | 0.54 | 4.8e-09 |
| 208 | AKT2 | v-akt murine thymoma viral oncogene homolog 2 | 0.08 | 5.2e-01 |
| 23533 | PIK3R5 | phosphoinositide-3-kinase, regulatory subunit 5 | 0.24 | 2.6e-01 |
| 2475 | MTOR | mechanistic target of rapamycin (serine/threonine kinase) | 0.74 | 9.0e-12 |
| 253260 | RICTOR | RPTOR independent companion of MTOR, complex 2 | -1.16 | 1.1e-19 |
| 253314 | EIF4E1B | eukaryotic translation initiation factor 4E family member 1B | 0.19 | 4.7e-01 |
| 25989 | ULK3 | unc-51 like kinase 3 | 0.60 | 3.7e-06 |
| 27330 | RPS6KA6 | ribosomal protein S6 kinase, 90kDa, polypeptide 6 | -4.25 | 7.1e-37 |
| 3091 | HIF1A | hypoxia inducible factor 1, alpha subunit (basic helix-loop-helix transcription factor) | 0.28 | 2.5e-01 |
| 3479 | IGF1 | insulin-like growth factor 1 (somatomedin C) | -2.10 | 3.2e-15 |
| 3551 | IKBKB | inhibitor of kappa light polypeptide gene enhancer in B-cells, kinase beta | -0.27 | 2.9e-03 |
| 3630 | INS | insulin | 0.08 | 6.2e-01 |
| 3667 | IRS1 | insulin receptor substrate 1 | -2.16 | 2.8e-24 |
| 5170 | PDPK1 | 3-phosphoinositide dependent protein kinase 1 | -0.68 | 8.0e-12 |
| 51719 | CAB39 | calcium binding protein 39 | -0.44 | 1.9e-07 |
| 5290 | PIK3CA | phosphatidylinositol-4,5-bisphosphate 3-kinase, catalytic subunit alpha | -0.34 | 1.2e-02 |
| 5291 | PIK3CB | phosphatidylinositol-4,5-bisphosphate 3-kinase, catalytic subunit beta | 0.19 | 8.7e-02 |
| 5293 | PIK3CD | phosphatidylinositol-4,5-bisphosphate 3-kinase, catalytic subunit delta | -0.71 | 1.6e-06 |
| 5294 | PIK3CG | phosphatidylinositol-4,5-bisphosphate 3-kinase, catalytic subunit gamma | -0.98 | 4.5e-05 |
| 5295 | PIK3R1 | phosphoinositide-3-kinase, regulatory subunit 1 (alpha) | -0.80 | 2.1e-03 |
| 5296 | PIK3R2 | phosphoinositide-3-kinase, regulatory subunit 2 (beta) | 0.67 | 6.5e-08 |
| 54541 | DDIT4 | DNA-damage-inducible transcript 4 | 1.10 | 8.0e-06 |
| 5562 | PRKAA1 | protein kinase, AMP-activated, alpha 1 catalytic subunit | -0.79 | 1.2e-10 |
| 5563 | PRKAA2 | protein kinase, AMP-activated, alpha 2 catalytic subunit | -0.64 | 3.1e-03 |
| 5578 | PRKCA | protein kinase C, alpha | -1.99 | 8.5e-30 |
| 5579 | PRKCB | protein kinase C, beta | -1.76 | 5.6e-18 |
| 5582 | PRKCG | protein kinase C, gamma | 0.28 | 5.7e-01 |
| 5594 | MAPK1 | mitogen-activated protein kinase 1 | -0.24 | 2.6e-02 |
| 5595 | MAPK3 | mitogen-activated protein kinase 3 | -0.86 | 1.2e-13 |
| 5728 | PTEN | phosphatase and tensin homolog | -0.86 | 7.4e-09 |
| 57521 | RPTOR | regulatory associated protein of MTOR, complex 1 | -0.24 | 9.8e-03 |
| 58528 | RRAGD | Ras-related GTP binding D | 0.64 | 9.3e-03 |
| 6009 | RHEB | Ras homolog enriched in brain | 0.13 | 3.0e-01 |
| 6194 | RPS6 | ribosomal protein S6 | -0.28 | 4.6e-02 |
| 6195 | RPS6KA1 | ribosomal protein S6 kinase, 90kDa, polypeptide 1 | 1.93 | 5.5e-38 |
| 6196 | RPS6KA2 | ribosomal protein S6 kinase, 90kDa, polypeptide 2 | -1.40 | 9.1e-26 |
| 6197 | RPS6KA3 | ribosomal protein S6 kinase, 90kDa, polypeptide 3 | -0.98 | 4.1e-15 |
| 6198 | RPS6KB1 | ribosomal protein S6 kinase, 70kDa, polypeptide 1 | -0.37 | 1.3e-04 |
| 6199 | RPS6KB2 | ribosomal protein S6 kinase, 70kDa, polypeptide 2 | 1.03 | 2.9e-17 |
| 64121 | RRAGC | Ras-related GTP binding C | -0.55 | 8.2e-04 |
| 64223 | MLST8 | MTOR associated protein, LST8 homolog (S. cerevisiae) | 0.58 | 9.6e-06 |
| 673 | BRAF | B-Raf proto-oncogene, serine/threonine kinase | 0.82 | 1.7e-09 |
| 6794 | STK11 | serine/threonine kinase 11 | 0.12 | 3.0e-01 |
| 7124 | TNF | tumor necrosis factor | 1.73 | 1.2e-05 |
| 7248 | TSC1 | tuberous sclerosis 1 | -0.67 | 1.5e-14 |
| 7249 | TSC2 | tuberous sclerosis 2 | 0.35 | 2.2e-03 |
| 7422 | VEGFA | vascular endothelial growth factor A | 0.57 | 2.4e-03 |
| 81617 | CAB39L | calcium binding protein 39-like | -1.80 | 3.4e-57 |
| 8408 | ULK1 | unc-51 like autophagy activating kinase 1 | -0.28 | 9.3e-03 |
| 84335 | AKT1S1 | AKT1 substrate 1 (proline-rich) | 0.35 | 1.8e-03 |
| 8503 | PIK3R3 | phosphoinositide-3-kinase, regulatory subunit 3 (gamma) | 1.28 | 1.7e-10 |
| 92335 | STRADA | STE20-related kinase adaptor alpha | 0.11 | 2.2e-01 |
| 9470 | EIF4E2 | eukaryotic translation initiation factor 4E family member 2 | 1.11 | 1.0e-21 |
| 9706 | ULK2 | unc-51 like autophagy activating kinase 2 | -0.91 | 1.5e-17 |

| ENTREZID | SYMBOL | GENENAME | FC | ADJ.PVAL |
| --- | --- | --- | --- | --- |

(Page generated on Mon Aug 24 22:00:29 2015 by ReportingTools 2.9.1 and hwriter 1.3.2)
