# Supplementary material for: Bioconductor’s EnrichmentBrowser: seamless navigation through combined results of set- & network-based enrichment analysis
Source: BMC Bioinformatics. 2016 Jan 20;17:45. doi: 10.1186/s12859-016-0884-1 (PMC4721010; doi:10.1186/s12859-016-0884-1)
Supplement: Supplementary file 3 — EnrichmentBrowser output (TCGA RNA-seq data). Unzip and open the contained index.html in the browser to view the contents of this file (tested with Firefox 39.0). (ZIP 7116.8 kb) [file 12859_2016_884_MOESM3_ESM.zip › hsa04270.html]

hsa04270: Gene Report


## hsa04270: Gene Report

| ENTREZID | SYMBOL | GENENAME | FC | ADJ.PVAL |
| --- | --- | --- | --- | --- |
| ENTREZID | SYMBOL | GENENAME | FC | ADJ.PVAL |
| 100137049 | PLA2G4B | phospholipase A2, group IVB (cytosolic) | -0.39 | 1.5e-01 |
| 10203 | CALCRL | calcitonin receptor-like | -2.17 | 6.2e-33 |
| 10242 | KCNMB2 | potassium channel subfamily M regulatory beta subunit 2 | -2.64 | 3.3e-20 |
| 10266 | RAMP2 | receptor (G protein-coupled) activity modifying protein 2 | -1.67 | 2.8e-27 |
| 10267 | RAMP1 | receptor (G protein-coupled) activity modifying protein 1 | -3.56 | 1.1e-46 |
| 10268 | RAMP3 | receptor (G protein-coupled) activity modifying protein 3 | -0.97 | 6.0e-06 |
| 10335 | MRVI1 | murine retrovirus integration site 1 homolog | -3.56 | 1.6e-84 |
| 10398 | MYL9 | myosin, light chain 9, regulatory | -3.46 | 6.2e-42 |
| 10672 | GNA13 | guanine nucleotide binding protein (G protein), alpha 13 | -0.42 | 2.3e-04 |
| 107 | ADCY1 | adenylate cyclase 1 (brain) | -0.73 | 2.1e-02 |
| 108 | ADCY2 | adenylate cyclase 2 (brain) | -4.06 | 2.1e-64 |
| 109 | ADCY3 | adenylate cyclase 3 | -1.35 | 8.4e-24 |
| 111 | ADCY5 | adenylate cyclase 5 | -1.97 | 1.3e-20 |
| 112 | ADCY6 | adenylate cyclase 6 | -0.27 | 2.2e-02 |
| 113 | ADCY7 | adenylate cyclase 7 | 0.15 | 3.4e-01 |
| 114 | ADCY8 | adenylate cyclase 8 (brain) | -2.84 | 6.3e-10 |
| 115 | ADCY9 | adenylate cyclase 9 | -2.25 | 1.1e-33 |
| 123745 | PLA2G4E | phospholipase A2, group IVE | 1.15 | 1.7e-04 |
| 135 | ADORA2A | adenosine A2a receptor | -0.50 | 2.1e-03 |
| 136 | ADORA2B | adenosine A2b receptor | 0.16 | 5.4e-01 |
| 140465 | MYL6B | myosin, light chain 6B, alkali, smooth muscle and non-muscle | 0.80 | 1.9e-07 |
| 146 | ADRA1D | adrenoceptor alpha 1D | -4.47 | 7.5e-45 |
| 147 | ADRA1B | adrenoceptor alpha 1B | -1.17 | 1.1e-03 |
| 148 | ADRA1A | adrenoceptor alpha 1A | -1.95 | 4.6e-09 |
| 157855 | KCNU1 | potassium channel, subfamily U, member 1 | 0.98 | 1.0e-03 |
| 1579 | CYP4A11 | cytochrome P450, family 4, subfamily A, polypeptide 11 | 0.11 | 7.7e-01 |
| 163688 | CALML6 | calmodulin-like 6 | 0.73 | 1.2e-02 |
| 185 | AGTR1 | angiotensin II receptor, type 1 | -3.96 | 1.2e-29 |
| 1909 | EDNRA | endothelin receptor type A | -3.16 | 1.3e-76 |
| 196883 | ADCY4 | adenylate cyclase 4 | -1.78 | 1.3e-29 |
| 23236 | PLCB1 | phospholipase C, beta 1 (phosphoinositide-specific) | 0.13 | 5.9e-01 |
| 23365 | ARHGEF12 | Rho guanine nucleotide exchange factor (GEF) 12 | -0.53 | 2.9e-06 |
| 255189 | PLA2G4F | phospholipase A2, group IVF | 3.67 | 7.2e-18 |
| 26279 | PLA2G2D | phospholipase A2, group IID | 1.39 | 6.1e-03 |
| 27094 | KCNMB3 | potassium channel subfamily M regulatory beta subunit 3 | -0.05 | 7.2e-01 |
| 27345 | KCNMB4 | potassium channel subfamily M regulatory beta subunit 4 | -0.88 | 2.7e-03 |
| 2767 | GNA11 | guanine nucleotide binding protein (G protein), alpha 11 (Gq class) | -0.83 | 3.2e-07 |
| 2768 | GNA12 | guanine nucleotide binding protein (G protein) alpha 12 | -0.70 | 1.3e-12 |
| 2776 | GNAQ | guanine nucleotide binding protein (G protein), q polypeptide | -0.64 | 8.9e-05 |
| 2778 | GNAS | GNAS complex locus | 0.76 | 5.9e-09 |
| 283748 | PLA2G4D | phospholipase A2, group IVD (cytosolic) | 1.15 | 2.7e-03 |
| 284541 | CYP4A22 | cytochrome P450, family 4, subfamily A, polypeptide 22 | 1.02 | 2.1e-05 |
| 2977 | GUCY1A2 | guanylate cyclase 1, soluble, alpha 2 | -3.49 | 5.4e-75 |
| 2982 | GUCY1A3 | guanylate cyclase 1, soluble, alpha 3 | -1.26 | 8.1e-11 |
| 2983 | GUCY1B3 | guanylate cyclase 1, soluble, beta 3 | -1.26 | 2.1e-15 |
| 30814 | PLA2G2E | phospholipase A2, group IIE | 0.76 | 1.1e-04 |
| 340156 | MYLK4 | myosin light chain kinase family, member 4 | -0.58 | 6.6e-03 |
| 369 | ARAF | A-Raf proto-oncogene, serine/threonine kinase | -0.12 | 2.8e-01 |
| 3708 | ITPR1 | inositol 1,4,5-trisphosphate receptor, type 1 | -2.91 | 1.2e-76 |
| 3709 | ITPR2 | inositol 1,4,5-trisphosphate receptor, type 2 | -0.73 | 2.6e-05 |
| 3710 | ITPR3 | inositol 1,4,5-trisphosphate receptor, type 3 | 1.58 | 3.7e-18 |
| 3778 | KCNMA1 | potassium channel, calcium activated large conductance subfamily M alpha, member 1 | -4.05 | 3.5e-70 |
| 3779 | KCNMB1 | potassium channel subfamily M regulatory beta subunit 1 | -4.30 | 6.7e-93 |
| 387 | RHOA | ras homolog family member A | -0.24 | 2.2e-03 |
| 391013 | PLA2G2C | phospholipase A2, group IIC | -0.32 | 3.6e-01 |
| 4629 | MYH11 | myosin, heavy chain 11, smooth muscle | -6.39 | 7.4e-70 |
| 4637 | MYL6 | myosin, light chain 6, alkali, smooth muscle and non-muscle | -0.20 | 1.1e-01 |
| 4638 | MYLK | myosin light chain kinase | -4.35 | 3.0e-79 |
| 4659 | PPP1R12A | protein phosphatase 1, regulatory subunit 12A | -1.38 | 8.5e-37 |
| 4660 | PPP1R12B | protein phosphatase 1, regulatory subunit 12B | -4.10 | 4.3e-98 |
| 4881 | NPR1 | natriuretic peptide receptor 1 | -2.92 | 3.2e-31 |
| 4882 | NPR2 | natriuretic peptide receptor 2 | -2.34 | 1.2e-67 |
| 50487 | PLA2G3 | phospholipase A2, group III | 4.23 | 3.7e-30 |
| 51806 | CALML5 | calmodulin-like 5 | 2.63 | 2.8e-08 |
| 5319 | PLA2G1B | phospholipase A2, group IB (pancreas) | -0.67 | 2.8e-02 |
| 5320 | PLA2G2A | phospholipase A2, group IIA (platelets, synovial fluid) | -2.46 | 9.4e-08 |
| 5321 | PLA2G4A | phospholipase A2, group IVA (cytosolic, calcium-dependent) | -0.47 | 1.6e-01 |
| 5322 | PLA2G5 | phospholipase A2, group V | -2.20 | 4.8e-09 |
| 5330 | PLCB2 | phospholipase C, beta 2 | 0.12 | 5.6e-01 |
| 5331 | PLCB3 | phospholipase C, beta 3 (phosphatidylinositol-specific) | 0.28 | 1.9e-02 |
| 5332 | PLCB4 | phospholipase C, beta 4 | 0.18 | 4.4e-01 |
| 54776 | PPP1R12C | protein phosphatase 1, regulatory subunit 12C | -1.22 | 6.9e-32 |
| 5499 | PPP1CA | protein phosphatase 1, catalytic subunit, alpha isozyme | 1.41 | 9.0e-36 |
| 5500 | PPP1CB | protein phosphatase 1, catalytic subunit, beta isozyme | -0.25 | 5.2e-02 |
| 5501 | PPP1CC | protein phosphatase 1, catalytic subunit, gamma isozyme | -0.17 | 1.0e-01 |
| 552 | AVPR1A | arginine vasopressin receptor 1A | -3.68 | 1.1e-40 |
| 553 | AVPR1B | arginine vasopressin receptor 1B | 2.12 | 7.8e-08 |
| 5566 | PRKACA | protein kinase, cAMP-dependent, catalytic, alpha | -0.17 | 1.0e-01 |
| 5567 | PRKACB | protein kinase, cAMP-dependent, catalytic, beta | -1.29 | 1.2e-22 |
| 5568 | PRKACG | protein kinase, cAMP-dependent, catalytic, gamma | 0.05 | 8.3e-01 |
| 5578 | PRKCA | protein kinase C, alpha | -1.99 | 8.5e-30 |
| 5579 | PRKCB | protein kinase C, beta | -1.76 | 5.6e-18 |
| 5580 | PRKCD | protein kinase C, delta | 1.29 | 1.5e-16 |
| 5581 | PRKCE | protein kinase C, epsilon | -0.81 | 7.9e-11 |
| 5582 | PRKCG | protein kinase C, gamma | 0.28 | 5.7e-01 |
| 5583 | PRKCH | protein kinase C, eta | -0.95 | 2.9e-13 |
| 5588 | PRKCQ | protein kinase C, theta | -0.57 | 8.2e-02 |
| 5592 | PRKG1 | protein kinase, cGMP-dependent, type I | -3.67 | 4.7e-93 |
| 5594 | MAPK1 | mitogen-activated protein kinase 1 | -0.24 | 2.6e-02 |
| 5595 | MAPK3 | mitogen-activated protein kinase 3 | -0.86 | 1.2e-13 |
| 5604 | MAP2K1 | mitogen-activated protein kinase kinase 1 | -0.05 | 6.3e-01 |
| 5605 | MAP2K2 | mitogen-activated protein kinase kinase 2 | 0.76 | 2.3e-06 |
| 5613 | PRKX | protein kinase, X-linked | 0.96 | 1.9e-08 |
| 5739 | PTGIR | prostaglandin I2 (prostacyclin) receptor (IP) | -0.31 | 1.0e-01 |
| 5894 | RAF1 | Raf-1 proto-oncogene, serine/threonine kinase | -0.11 | 1.2e-01 |
| 59 | ACTA2 | actin, alpha 2, smooth muscle, aorta | -4.13 | 2.1e-47 |
| 6093 | ROCK1 | Rho-associated, coiled-coil containing protein kinase 1 | -0.82 | 1.6e-12 |
| 64600 | PLA2G2F | phospholipase A2, group IIF | 1.89 | 5.4e-09 |
| 673 | BRAF | B-Raf proto-oncogene, serine/threonine kinase | 0.82 | 1.7e-09 |
| 72 | ACTG2 | actin, gamma 2, smooth muscle, enteric | -5.42 | 6.9e-66 |
| 775 | CACNA1C | calcium channel, voltage-dependent, L type, alpha 1C subunit | -2.86 | 5.9e-54 |
| 776 | CACNA1D | calcium channel, voltage-dependent, L type, alpha 1D subunit | -1.31 | 1.4e-07 |
| 778 | CACNA1F | calcium channel, voltage-dependent, L type, alpha 1F subunit | -0.33 | 2.4e-01 |
| 779 | CACNA1S | calcium channel, voltage-dependent, L type, alpha 1S subunit | 0.94 | 4.2e-02 |
| 800 | CALD1 | caldesmon 1 | -2.84 | 3.5e-55 |
| 801 | CALM1 | calmodulin 1 (phosphorylase kinase, delta) | 0.18 | 5.9e-02 |
| 805 | CALM2 | calmodulin 2 (phosphorylase kinase, delta) | -0.31 | 3.5e-04 |
| 808 | CALM3 | calmodulin 3 (phosphorylase kinase, delta) | -0.07 | 5.5e-01 |
| 810 | CALML3 | calmodulin-like 3 | 2.15 | 5.2e-04 |
| 81579 | PLA2G12A | phospholipase A2, group XIIA | 0.07 | 5.2e-01 |
| 8398 | PLA2G6 | phospholipase A2, group VI (cytosolic, calcium-independent) | -0.03 | 8.8e-01 |
| 8399 | PLA2G10 | phospholipase A2, group X | 1.73 | 3.4e-07 |
| 84647 | PLA2G12B | phospholipase A2, group XIIB | 0.45 | 8.3e-02 |
| 85366 | MYLK2 | myosin light chain kinase 2 | 1.75 | 2.1e-14 |
| 8605 | PLA2G4C | phospholipase A2, group IVC (cytosolic, calcium-independent) | -1.79 | 4.8e-31 |
| 8681 | JMJD7-PLA2G4B | JMJD7-PLA2G4B readthrough | -0.31 | 6.3e-02 |
| 9138 | ARHGEF1 | Rho guanine nucleotide exchange factor (GEF) 1 | 0.13 | 2.6e-01 |
| 91807 | MYLK3 | myosin light chain kinase 3 | -0.55 | 1.1e-01 |
| 94274 | PPP1R14A | protein phosphatase 1, regulatory (inhibitor) subunit 14A | -2.05 | 1.6e-21 |
| 9475 | ROCK2 | Rho-associated, coiled-coil containing protein kinase 2 | -1.35 | 3.4e-18 |
| 9826 | ARHGEF11 | Rho guanine nucleotide exchange factor (GEF) 11 | 0.21 | 5.2e-02 |

| ENTREZID | SYMBOL | GENENAME | FC | ADJ.PVAL |
| --- | --- | --- | --- | --- |

(Page generated on Tue Aug 25 12:05:02 2015 by ReportingTools 2.9.1 and hwriter 1.3.2)
