# Supplementary material for: Bioconductor’s EnrichmentBrowser: seamless navigation through combined results of set- & network-based enrichment analysis
Source: BMC Bioinformatics. 2016 Jan 20;17:45. doi: 10.1186/s12859-016-0884-1 (PMC4721010; doi:10.1186/s12859-016-0884-1)
Supplement: Supplementary file 3 — EnrichmentBrowser output (TCGA RNA-seq data). Unzip and open the contained index.html in the browser to view the contents of this file (tested with Firefox 39.0). (ZIP 7116.8 kb) [file 12859_2016_884_MOESM3_ESM.zip › hsa04270_gview.html]

hsa04270 
