# Supplementary material for: Bioconductor’s EnrichmentBrowser: seamless navigation through combined results of set- & network-based enrichment analysis
Source: BMC Bioinformatics. 2016 Jan 20;17:45. doi: 10.1186/s12859-016-0884-1 (PMC4721010; doi:10.1186/s12859-016-0884-1)
Supplement: Supplementary file 3 — EnrichmentBrowser output (TCGA RNA-seq data). Unzip and open the contained index.html in the browser to view the contents of this file (tested with Firefox 39.0). (ZIP 7116.8 kb) [file 12859_2016_884_MOESM3_ESM.zip › hsa04713.html]

hsa04713: Gene Report


## hsa04713: Gene Report

| ENTREZID | SYMBOL | GENENAME | FC | ADJ.PVAL |
| --- | --- | --- | --- | --- |
| ENTREZID | SYMBOL | GENENAME | FC | ADJ.PVAL |
| 10681 | GNB5 | guanine nucleotide binding protein (G protein), beta 5 | -0.95 | 7.0e-10 |
| 107 | ADCY1 | adenylate cyclase 1 (brain) | -0.73 | 2.1e-02 |
| 108 | ADCY2 | adenylate cyclase 2 (brain) | -4.06 | 2.1e-64 |
| 109 | ADCY3 | adenylate cyclase 3 | -1.35 | 8.4e-24 |
| 111 | ADCY5 | adenylate cyclase 5 | -1.97 | 1.3e-20 |
| 112 | ADCY6 | adenylate cyclase 6 | -0.27 | 2.2e-02 |
| 113 | ADCY7 | adenylate cyclase 7 | 0.15 | 3.4e-01 |
| 114 | ADCY8 | adenylate cyclase 8 (brain) | -2.84 | 6.3e-10 |
| 115 | ADCY9 | adenylate cyclase 9 | -2.25 | 1.1e-33 |
| 117 | ADCYAP1R1 | adenylate cyclase activating polypeptide 1 (pituitary) receptor type I | -2.14 | 1.9e-08 |
| 1385 | CREB1 | cAMP responsive element binding protein 1 | -0.50 | 9.6e-09 |
| 163688 | CALML6 | calmodulin-like 6 | 0.73 | 1.2e-02 |
| 196883 | ADCY4 | adenylate cyclase 4 | -1.78 | 1.3e-29 |
| 23236 | PLCB1 | phospholipase C, beta 1 (phosphoinositide-specific) | 0.13 | 5.9e-01 |
| 2353 | FOS | FBJ murine osteosarcoma viral oncogene homolog | -2.84 | 1.5e-21 |
| 2770 | GNAI1 | guanine nucleotide binding protein (G protein), alpha inhibiting activity polypeptide 1 | -0.76 | 1.6e-05 |
| 2771 | GNAI2 | guanine nucleotide binding protein (G protein), alpha inhibiting activity polypeptide 2 | -0.40 | 1.3e-04 |
| 2773 | GNAI3 | guanine nucleotide binding protein (G protein), alpha inhibiting activity polypeptide 3 | 0.15 | 6.1e-02 |
| 2775 | GNAO1 | guanine nucleotide binding protein (G protein), alpha activating activity polypeptide O | -3.23 | 6.2e-71 |
| 2776 | GNAQ | guanine nucleotide binding protein (G protein), q polypeptide | -0.64 | 8.9e-05 |
| 2778 | GNAS | GNAS complex locus | 0.76 | 5.9e-09 |
| 2782 | GNB1 | guanine nucleotide binding protein (G protein), beta polypeptide 1 | 0.21 | 7.6e-03 |
| 2783 | GNB2 | guanine nucleotide binding protein (G protein), beta polypeptide 2 | 0.46 | 9.7e-05 |
| 2784 | GNB3 | guanine nucleotide binding protein (G protein), beta polypeptide 3 | -0.61 | 2.3e-03 |
| 2785 | GNG3 | guanine nucleotide binding protein (G protein), gamma 3 | 1.12 | 8.9e-09 |
| 2786 | GNG4 | guanine nucleotide binding protein (G protein), gamma 4 | -2.01 | 7.8e-12 |
| 2787 | GNG5 | guanine nucleotide binding protein (G protein), gamma 5 | 0.70 | 9.5e-13 |
| 2788 | GNG7 | guanine nucleotide binding protein (G protein), gamma 7 | -3.43 | 1.1e-83 |
| 2790 | GNG10 | guanine nucleotide binding protein (G protein), gamma 10 | 0.60 | 1.0e-01 |
| 2791 | GNG11 | guanine nucleotide binding protein (G protein), gamma 11 | -2.53 | 2.0e-46 |
| 2792 | GNGT1 | guanine nucleotide binding protein (G protein), gamma transducing activity polypeptide 1 | 2.69 | 5.4e-19 |
| 2793 | GNGT2 | guanine nucleotide binding protein (G protein), gamma transducing activity polypeptide 2 | -0.53 | 8.4e-03 |
| 2890 | GRIA1 | glutamate receptor, ionotropic, AMPA 1 | -2.86 | 8.5e-13 |
| 2891 | GRIA2 | glutamate receptor, ionotropic, AMPA 2 | -1.48 | 2.0e-03 |
| 2892 | GRIA3 | glutamate receptor, ionotropic, AMPA 3 | -3.62 | 9.0e-33 |
| 2893 | GRIA4 | glutamate receptor, ionotropic, AMPA 4 | -1.25 | 1.4e-03 |
| 2902 | GRIN1 | glutamate receptor, ionotropic, N-methyl D-aspartate 1 | 3.06 | 2.6e-16 |
| 2903 | GRIN2A | glutamate receptor, ionotropic, N-methyl D-aspartate 2A | -3.44 | 2.8e-24 |
| 2904 | GRIN2B | glutamate receptor, ionotropic, N-methyl D-aspartate 2B | 0.88 | 6.8e-03 |
| 2905 | GRIN2C | glutamate receptor, ionotropic, N-methyl D-aspartate 2C | -0.12 | 7.4e-01 |
| 2906 | GRIN2D | glutamate receptor, ionotropic, N-methyl D-aspartate 2D | 3.73 | 1.3e-10 |
| 2977 | GUCY1A2 | guanylate cyclase 1, soluble, alpha 2 | -3.49 | 5.4e-75 |
| 2982 | GUCY1A3 | guanylate cyclase 1, soluble, alpha 3 | -1.26 | 8.1e-11 |
| 2983 | GUCY1B3 | guanylate cyclase 1, soluble, beta 3 | -1.26 | 2.1e-15 |
| 3708 | ITPR1 | inositol 1,4,5-trisphosphate receptor, type 1 | -2.91 | 1.2e-76 |
| 3710 | ITPR3 | inositol 1,4,5-trisphosphate receptor, type 3 | 1.58 | 3.7e-18 |
| 3760 | KCNJ3 | potassium channel, inwardly rectifying subfamily J, member 3 | -1.17 | 3.8e-03 |
| 3762 | KCNJ5 | potassium channel, inwardly rectifying subfamily J, member 5 | -0.04 | 9.0e-01 |
| 3763 | KCNJ6 | potassium channel, inwardly rectifying subfamily J, member 6 | 1.22 | 4.4e-05 |
| 3765 | KCNJ9 | potassium channel, inwardly rectifying subfamily J, member 9 | 0.62 | 2.2e-02 |
| 4543 | MTNR1A | melatonin receptor 1A | 0.32 | 3.1e-01 |
| 4544 | MTNR1B | melatonin receptor 1B | 0.51 | 2.2e-02 |
| 4842 | NOS1 | nitric oxide synthase 1 (neuronal) | -1.69 | 2.2e-05 |
| 51655 | RASD1 | RAS, dexamethasone-induced 1 | -0.28 | 4.5e-01 |
| 51764 | GNG13 | guanine nucleotide binding protein (G protein), gamma 13 | 1.23 | 5.6e-05 |
| 51806 | CALML5 | calmodulin-like 5 | 2.63 | 2.8e-08 |
| 5187 | PER1 | period circadian clock 1 | -1.98 | 9.0e-45 |
| 5330 | PLCB2 | phospholipase C, beta 2 | 0.12 | 5.6e-01 |
| 5331 | PLCB3 | phospholipase C, beta 3 (phosphatidylinositol-specific) | 0.28 | 1.9e-02 |
| 5332 | PLCB4 | phospholipase C, beta 4 | 0.18 | 4.4e-01 |
| 54331 | GNG2 | guanine nucleotide binding protein (G protein), gamma 2 | -1.46 | 2.0e-20 |
| 5566 | PRKACA | protein kinase, cAMP-dependent, catalytic, alpha | -0.17 | 1.0e-01 |
| 5567 | PRKACB | protein kinase, cAMP-dependent, catalytic, beta | -1.29 | 1.2e-22 |
| 5568 | PRKACG | protein kinase, cAMP-dependent, catalytic, gamma | 0.05 | 8.3e-01 |
| 5578 | PRKCA | protein kinase C, alpha | -1.99 | 8.5e-30 |
| 5579 | PRKCB | protein kinase C, beta | -1.76 | 5.6e-18 |
| 55811 | ADCY10 | adenylate cyclase 10 (soluble) | 0.41 | 1.1e-01 |
| 5582 | PRKCG | protein kinase C, gamma | 0.28 | 5.7e-01 |
| 5592 | PRKG1 | protein kinase, cGMP-dependent, type I | -3.67 | 4.7e-93 |
| 5593 | PRKG2 | protein kinase, cGMP-dependent, type II | -0.55 | 1.7e-01 |
| 5594 | MAPK1 | mitogen-activated protein kinase 1 | -0.24 | 2.6e-02 |
| 5595 | MAPK3 | mitogen-activated protein kinase 3 | -0.86 | 1.2e-13 |
| 55970 | GNG12 | guanine nucleotide binding protein (G protein), gamma 12 | -1.39 | 1.5e-19 |
| 5613 | PRKX | protein kinase, X-linked | 0.96 | 1.9e-08 |
| 59345 | GNB4 | guanine nucleotide binding protein (G protein), beta polypeptide 4 | -0.90 | 1.3e-07 |
| 6261 | RYR1 | ryanodine receptor 1 (skeletal) | 0.39 | 2.9e-01 |
| 6262 | RYR2 | ryanodine receptor 2 (cardiac) | -1.76 | 2.3e-16 |
| 6263 | RYR3 | ryanodine receptor 3 | -3.67 | 2.3e-98 |
| 775 | CACNA1C | calcium channel, voltage-dependent, L type, alpha 1C subunit | -2.86 | 5.9e-54 |
| 776 | CACNA1D | calcium channel, voltage-dependent, L type, alpha 1D subunit | -1.31 | 1.4e-07 |
| 801 | CALM1 | calmodulin 1 (phosphorylase kinase, delta) | 0.18 | 5.9e-02 |
| 805 | CALM2 | calmodulin 2 (phosphorylase kinase, delta) | -0.31 | 3.5e-04 |
| 808 | CALM3 | calmodulin 3 (phosphorylase kinase, delta) | -0.07 | 5.5e-01 |
| 810 | CALML3 | calmodulin-like 3 | 2.15 | 5.2e-04 |
| 815 | CAMK2A | calcium/calmodulin-dependent protein kinase II alpha | -4.90 | 5.4e-81 |
| 816 | CAMK2B | calcium/calmodulin-dependent protein kinase II beta | 1.05 | 1.4e-02 |
| 817 | CAMK2D | calcium/calmodulin-dependent protein kinase II delta | -0.96 | 7.9e-11 |
| 818 | CAMK2G | calcium/calmodulin-dependent protein kinase II gamma | -0.99 | 4.9e-27 |
| 8863 | PER3 | period circadian clock 3 | -2.23 | 2.2e-42 |
| 8864 | PER2 | period circadian clock 2 | -0.80 | 2.1e-13 |
| 8911 | CACNA1I | calcium channel, voltage-dependent, T type, alpha 1I subunit | 0.82 | 4.6e-02 |
| 8912 | CACNA1H | calcium channel, voltage-dependent, T type, alpha 1H subunit | -4.11 | 6.8e-100 |
| 8913 | CACNA1G | calcium channel, voltage-dependent, T type, alpha 1G subunit | -3.17 | 3.0e-37 |
| 9252 | RPS6KA5 | ribosomal protein S6 kinase, 90kDa, polypeptide 5 | -1.01 | 6.3e-08 |
| 94235 | GNG8 | guanine nucleotide binding protein (G protein), gamma 8 | -1.03 | 2.8e-03 |
| 9722 | NOS1AP | nitric oxide synthase 1 (neuronal) adaptor protein | 0.85 | 5.4e-04 |

| ENTREZID | SYMBOL | GENENAME | FC | ADJ.PVAL |
| --- | --- | --- | --- | --- |

(Page generated on Tue Aug 25 12:05:02 2015 by ReportingTools 2.9.1 and hwriter 1.3.2)
