# Supplementary material for: Bioconductor’s EnrichmentBrowser: seamless navigation through combined results of set- & network-based enrichment analysis
Source: BMC Bioinformatics. 2016 Jan 20;17:45. doi: 10.1186/s12859-016-0884-1 (PMC4721010; doi:10.1186/s12859-016-0884-1)
Supplement: Supplementary file 3 — EnrichmentBrowser output (TCGA RNA-seq data). Unzip and open the contained index.html in the browser to view the contents of this file (tested with Firefox 39.0). (ZIP 7116.8 kb) [file 12859_2016_884_MOESM3_ESM.zip › hsa04722.html]

hsa04722: Gene Report


## hsa04722: Gene Report

| ENTREZID | SYMBOL | GENENAME | FC | ADJ.PVAL |
| --- | --- | --- | --- | --- |
| ENTREZID | SYMBOL | GENENAME | FC | ADJ.PVAL |
| 10000 | AKT3 | v-akt murine thymoma viral oncogene homolog 3 | -3.49 | 6.4e-108 |
| 10019 | SH2B3 | SH2B adaptor protein 3 | -0.96 | 1.4e-18 |
| 10603 | SH2B2 | SH2B adaptor protein 2 | 1.02 | 1.1e-06 |
| 10782 | ZNF274 | zinc finger protein 274 | -0.41 | 1.0e-04 |
| 10818 | FRS2 | fibroblast growth factor receptor substrate 2 | -0.64 | 9.2e-11 |
| 11108 | PRDM4 | PR domain containing 4 | -0.08 | 3.1e-01 |
| 11213 | IRAK3 | interleukin-1 receptor-associated kinase 3 | -1.72 | 2.0e-18 |
| 1398 | CRK | v-crk avian sarcoma virus CT10 oncogene homolog | -0.22 | 1.6e-02 |
| 1399 | CRKL | v-crk avian sarcoma virus CT10 oncogene homolog-like | -0.33 | 2.1e-04 |
| 1432 | MAPK14 | mitogen-activated protein kinase 14 | -0.56 | 2.9e-13 |
| 163688 | CALML6 | calmodulin-like 6 | 0.73 | 1.2e-02 |
| 207 | AKT1 | v-akt murine thymoma viral oncogene homolog 1 | 0.54 | 4.8e-09 |
| 208 | AKT2 | v-akt murine thymoma viral oncogene homolog 2 | 0.08 | 5.2e-01 |
| 2309 | FOXO3 | forkhead box O3 | -0.42 | 2.4e-05 |
| 23533 | PIK3R5 | phosphoinositide-3-kinase, regulatory subunit 5 | 0.24 | 2.6e-01 |
| 25 | ABL1 | ABL proto-oncogene 1, non-receptor tyrosine kinase | -1.00 | 8.4e-22 |
| 2549 | GAB1 | GRB2-associated binding protein 1 | -1.56 | 2.5e-39 |
| 25759 | SHC2 | SHC (Src homology 2 domain containing) transforming protein 2 | -2.29 | 2.9e-26 |
| 25970 | SH2B1 | SH2B adaptor protein 1 | -0.31 | 4.4e-03 |
| 27018 | NGFRAP1 | nerve growth factor receptor (TNFRSF16) associated protein 1 | -0.25 | 1.5e-01 |
| 27330 | RPS6KA6 | ribosomal protein S6 kinase, 90kDa, polypeptide 6 | -4.25 | 7.1e-37 |
| 2885 | GRB2 | growth factor receptor-bound protein 2 | 0.00 | 9.9e-01 |
| 2889 | RAPGEF1 | Rap guanine nucleotide exchange factor (GEF) 1 | -0.48 | 2.7e-08 |
| 2932 | GSK3B | glycogen synthase kinase 3 beta | 0.22 | 2.5e-02 |
| 3265 | HRAS | Harvey rat sarcoma viral oncogene homolog | 0.90 | 2.8e-08 |
| 3551 | IKBKB | inhibitor of kappa light polypeptide gene enhancer in B-cells, kinase beta | -0.27 | 2.9e-03 |
| 356 | FASLG | Fas ligand (TNF superfamily, member 6) | -0.18 | 6.0e-01 |
| 3654 | IRAK1 | interleukin-1 receptor-associated kinase 1 | 0.90 | 6.1e-11 |
| 3656 | IRAK2 | interleukin-1 receptor-associated kinase 2 | -0.49 | 4.4e-02 |
| 3667 | IRS1 | insulin receptor substrate 1 | -2.16 | 2.8e-24 |
| 3725 | JUN | jun proto-oncogene | -1.50 | 1.0e-16 |
| 3845 | KRAS | Kirsten rat sarcoma viral oncogene homolog | 0.33 | 1.3e-02 |
| 387 | RHOA | ras homolog family member A | -0.24 | 2.2e-03 |
| 396 | ARHGDIA | Rho GDP dissociation inhibitor (GDI) alpha | 0.42 | 9.1e-04 |
| 397 | ARHGDIB | Rho GDP dissociation inhibitor (GDI) beta | -0.17 | 3.2e-01 |
| 398 | ARHGDIG | Rho GDP dissociation inhibitor (GDI) gamma | 1.88 | 1.5e-06 |
| 399694 | SHC4 | SHC (Src homology 2 domain containing) family, member 4 | -1.02 | 1.9e-05 |
| 4145 | MATK | megakaryocyte-associated tyrosine kinase | -0.99 | 2.0e-05 |
| 4214 | MAP3K1 | mitogen-activated protein kinase kinase kinase 1, E3 ubiquitin protein ligase | -0.02 | 9.0e-01 |
| 4215 | MAP3K3 | mitogen-activated protein kinase kinase kinase 3 | -1.48 | 3.1e-58 |
| 4217 | MAP3K5 | mitogen-activated protein kinase kinase kinase 5 | -1.22 | 6.6e-17 |
| 468 | ATF4 | activating transcription factor 4 | 0.12 | 2.9e-01 |
| 4790 | NFKB1 | nuclear factor of kappa light polypeptide gene enhancer in B-cells 1 | -0.70 | 5.4e-11 |
| 4792 | NFKBIA | nuclear factor of kappa light polypeptide gene enhancer in B-cells inhibitor, alpha | -0.36 | 7.9e-03 |
| 4793 | NFKBIB | nuclear factor of kappa light polypeptide gene enhancer in B-cells inhibitor, beta | 0.73 | 3.4e-08 |
| 4794 | NFKBIE | nuclear factor of kappa light polypeptide gene enhancer in B-cells inhibitor, epsilon | 1.04 | 2.4e-11 |
| 4803 | NGF | nerve growth factor (beta polypeptide) | -2.85 | 8.8e-31 |
| 4804 | NGFR | nerve growth factor receptor | -3.09 | 2.4e-31 |
| 4893 | NRAS | neuroblastoma RAS viral (v-ras) oncogene homolog | 0.76 | 2.6e-07 |
| 4908 | NTF3 | neurotrophin 3 | -1.39 | 4.7e-05 |
| 4909 | NTF4 | neurotrophin 4 | -0.44 | 3.3e-01 |
| 4914 | NTRK1 | neurotrophic tyrosine kinase, receptor, type 1 | -0.24 | 4.5e-01 |
| 4915 | NTRK2 | neurotrophic tyrosine kinase, receptor, type 2 | -1.60 | 5.5e-08 |
| 4916 | NTRK3 | neurotrophic tyrosine kinase, receptor, type 3 | -3.70 | 7.5e-44 |
| 51135 | IRAK4 | interleukin-1 receptor-associated kinase 4 | -0.13 | 1.8e-01 |
| 5170 | PDPK1 | 3-phosphoinositide dependent protein kinase 1 | -0.68 | 8.0e-12 |
| 51806 | CALML5 | calmodulin-like 5 | 2.63 | 2.8e-08 |
| 5290 | PIK3CA | phosphatidylinositol-4,5-bisphosphate 3-kinase, catalytic subunit alpha | -0.34 | 1.2e-02 |
| 5291 | PIK3CB | phosphatidylinositol-4,5-bisphosphate 3-kinase, catalytic subunit beta | 0.19 | 8.7e-02 |
| 5293 | PIK3CD | phosphatidylinositol-4,5-bisphosphate 3-kinase, catalytic subunit delta | -0.71 | 1.6e-06 |
| 5294 | PIK3CG | phosphatidylinositol-4,5-bisphosphate 3-kinase, catalytic subunit gamma | -0.98 | 4.5e-05 |
| 5295 | PIK3R1 | phosphoinositide-3-kinase, regulatory subunit 1 (alpha) | -0.80 | 2.1e-03 |
| 5296 | PIK3R2 | phosphoinositide-3-kinase, regulatory subunit 2 (beta) | 0.67 | 6.5e-08 |
| 5335 | PLCG1 | phospholipase C, gamma 1 | -0.02 | 9.1e-01 |
| 53358 | SHC3 | SHC (Src homology 2 domain containing) transforming protein 3 | -1.66 | 1.2e-09 |
| 5336 | PLCG2 | phospholipase C, gamma 2 (phosphatidylinositol-specific) | -0.70 | 1.7e-05 |
| 5580 | PRKCD | protein kinase C, delta | 1.29 | 1.5e-16 |
| 5594 | MAPK1 | mitogen-activated protein kinase 1 | -0.24 | 2.6e-02 |
| 5595 | MAPK3 | mitogen-activated protein kinase 3 | -0.86 | 1.2e-13 |
| 5598 | MAPK7 | mitogen-activated protein kinase 7 | -0.23 | 3.1e-02 |
| 5599 | MAPK8 | mitogen-activated protein kinase 8 | 0.06 | 7.3e-01 |
| 5600 | MAPK11 | mitogen-activated protein kinase 11 | -1.30 | 3.5e-16 |
| 5601 | MAPK9 | mitogen-activated protein kinase 9 | -0.12 | 1.0e-01 |
| 5602 | MAPK10 | mitogen-activated protein kinase 10 | -2.79 | 1.3e-41 |
| 5603 | MAPK13 | mitogen-activated protein kinase 13 | 2.49 | 3.5e-23 |
| 5604 | MAP2K1 | mitogen-activated protein kinase kinase 1 | -0.05 | 6.3e-01 |
| 5605 | MAP2K2 | mitogen-activated protein kinase kinase 2 | 0.76 | 2.3e-06 |
| 5607 | MAP2K5 | mitogen-activated protein kinase kinase 5 | -0.64 | 5.3e-14 |
| 5609 | MAP2K7 | mitogen-activated protein kinase kinase 7 | -0.36 | 7.2e-05 |
| 5663 | PSEN1 | presenilin 1 | 0.26 | 6.4e-05 |
| 5664 | PSEN2 | presenilin 2 | 0.02 | 9.1e-01 |
| 572 | BAD | BCL2-associated agonist of cell death | 0.17 | 2.1e-01 |
| 57498 | KIDINS220 | kinase D-interacting substrate, 220kDa | -0.75 | 1.1e-08 |
| 5781 | PTPN11 | protein tyrosine phosphatase, non-receptor type 11 | -0.53 | 1.8e-06 |
| 581 | BAX | BCL2-associated X protein | 0.89 | 9.4e-11 |
| 5879 | RAC1 | ras-related C3 botulinum toxin substrate 1 (rho family, small GTP binding protein Rac1) | 0.02 | 7.8e-01 |
| 5894 | RAF1 | Raf-1 proto-oncogene, serine/threonine kinase | -0.11 | 1.2e-01 |
| 5906 | RAP1A | RAP1A, member of RAS oncogene family | -0.72 | 8.3e-12 |
| 5908 | RAP1B | RAP1B, member of RAS oncogene family | -0.51 | 5.4e-09 |
| 596 | BCL2 | B-cell CLL/lymphoma 2 | -2.24 | 1.7e-33 |
| 5970 | RELA | v-rel avian reticuloendotheliosis viral oncogene homolog A | 0.00 | 9.6e-01 |
| 6195 | RPS6KA1 | ribosomal protein S6 kinase, 90kDa, polypeptide 1 | 1.93 | 5.5e-38 |
| 6196 | RPS6KA2 | ribosomal protein S6 kinase, 90kDa, polypeptide 2 | -1.40 | 9.1e-26 |
| 6197 | RPS6KA3 | ribosomal protein S6 kinase, 90kDa, polypeptide 3 | -0.98 | 4.1e-15 |
| 627 | BDNF | brain-derived neurotrophic factor | 1.70 | 2.2e-06 |
| 6272 | SORT1 | sortilin 1 | 1.04 | 4.5e-18 |
| 6300 | MAPK12 | mitogen-activated protein kinase 12 | -0.73 | 1.2e-03 |
| 6464 | SHC1 | SHC (Src homology 2 domain containing) transforming protein 1 | -0.10 | 3.4e-01 |
| 6654 | SOS1 | son of sevenless homolog 1 (Drosophila) | -0.44 | 1.5e-06 |
| 6655 | SOS2 | son of sevenless homolog 2 (Drosophila) | -0.83 | 1.9e-19 |
| 673 | BRAF | B-Raf proto-oncogene, serine/threonine kinase | 0.82 | 1.7e-09 |
| 7157 | TP53 | tumor protein p53 | 0.53 | 2.8e-03 |
| 7161 | TP73 | tumor protein p73 | 1.55 | 1.2e-04 |
| 7189 | TRAF6 | TNF receptor-associated factor 6, E3 ubiquitin protein ligase | -0.55 | 1.3e-13 |
| 7531 | YWHAE | tyrosine 3-monooxygenase/tryptophan 5-monooxygenase activation protein, epsilon | 0.81 | 1.3e-15 |
| 801 | CALM1 | calmodulin 1 (phosphorylase kinase, delta) | 0.18 | 5.9e-02 |
| 805 | CALM2 | calmodulin 2 (phosphorylase kinase, delta) | -0.31 | 3.5e-04 |
| 808 | CALM3 | calmodulin 3 (phosphorylase kinase, delta) | -0.07 | 5.5e-01 |
| 810 | CALML3 | calmodulin-like 3 | 2.15 | 5.2e-04 |
| 814 | CAMK4 | calcium/calmodulin-dependent protein kinase IV | -0.76 | 7.5e-03 |
| 815 | CAMK2A | calcium/calmodulin-dependent protein kinase II alpha | -4.90 | 5.4e-81 |
| 816 | CAMK2B | calcium/calmodulin-dependent protein kinase II beta | 1.05 | 1.4e-02 |
| 817 | CAMK2D | calcium/calmodulin-dependent protein kinase II delta | -0.96 | 7.9e-11 |
| 818 | CAMK2G | calcium/calmodulin-dependent protein kinase II gamma | -0.99 | 4.9e-27 |
| 8503 | PIK3R3 | phosphoinositide-3-kinase, regulatory subunit 3 (gamma) | 1.28 | 1.7e-10 |
| 8767 | RIPK2 | receptor-interacting serine-threonine kinase 2 | 1.02 | 3.0e-09 |
| 9252 | RPS6KA5 | ribosomal protein S6 kinase, 90kDa, polypeptide 5 | -1.01 | 6.3e-08 |
| 9261 | MAPKAPK2 | mitogen-activated protein kinase-activated protein kinase 2 | 0.37 | 8.3e-06 |
| 9500 | MAGED1 | melanoma antigen family D1 | 0.22 | 1.5e-01 |
| 998 | CDC42 | cell division cycle 42 | -0.03 | 8.1e-01 |

| ENTREZID | SYMBOL | GENENAME | FC | ADJ.PVAL |
| --- | --- | --- | --- | --- |

(Page generated on Mon Aug 24 22:02:41 2015 by ReportingTools 2.9.1 and hwriter 1.3.2)
