# Supplementary material for: Bioconductor’s EnrichmentBrowser: seamless navigation through combined results of set- & network-based enrichment analysis
Source: BMC Bioinformatics. 2016 Jan 20;17:45. doi: 10.1186/s12859-016-0884-1 (PMC4721010; doi:10.1186/s12859-016-0884-1)
Supplement: Supplementary file 3 — EnrichmentBrowser output (TCGA RNA-seq data). Unzip and open the contained index.html in the browser to view the contents of this file (tested with Firefox 39.0). (ZIP 7116.8 kb) [file 12859_2016_884_MOESM3_ESM.zip › hsa04915.html]

hsa04915: Gene Report


## hsa04915: Gene Report

| ENTREZID | SYMBOL | GENENAME | FC | ADJ.PVAL |
| --- | --- | --- | --- | --- |
| ENTREZID | SYMBOL | GENENAME | FC | ADJ.PVAL |
| 10000 | AKT3 | v-akt murine thymoma viral oncogene homolog 3 | -3.49 | 6.4e-108 |
| 10488 | CREB3 | cAMP responsive element binding protein 3 | 0.03 | 7.5e-01 |
| 107 | ADCY1 | adenylate cyclase 1 (brain) | -0.73 | 2.1e-02 |
| 108 | ADCY2 | adenylate cyclase 2 (brain) | -4.06 | 2.1e-64 |
| 109 | ADCY3 | adenylate cyclase 3 | -1.35 | 8.4e-24 |
| 111 | ADCY5 | adenylate cyclase 5 | -1.97 | 1.3e-20 |
| 112 | ADCY6 | adenylate cyclase 6 | -0.27 | 2.2e-02 |
| 113 | ADCY7 | adenylate cyclase 7 | 0.15 | 3.4e-01 |
| 114 | ADCY8 | adenylate cyclase 8 (brain) | -2.84 | 6.3e-10 |
| 115 | ADCY9 | adenylate cyclase 9 | -2.25 | 1.1e-33 |
| 1385 | CREB1 | cAMP responsive element binding protein 1 | -0.50 | 9.6e-09 |
| 1386 | ATF2 | activating transcription factor 2 | -0.43 | 7.5e-07 |
| 1388 | ATF6B | activating transcription factor 6 beta | 0.05 | 6.5e-01 |
| 148327 | CREB3L4 | cAMP responsive element binding protein 3-like 4 | 1.21 | 3.1e-07 |
| 163688 | CALML6 | calmodulin-like 6 | 0.73 | 1.2e-02 |
| 1839 | HBEGF | heparin-binding EGF-like growth factor | -1.27 | 6.4e-22 |
| 1956 | EGFR | epidermal growth factor receptor | -0.93 | 2.7e-06 |
| 196883 | ADCY4 | adenylate cyclase 4 | -1.78 | 1.3e-29 |
| 207 | AKT1 | v-akt murine thymoma viral oncogene homolog 1 | 0.54 | 4.8e-09 |
| 208 | AKT2 | v-akt murine thymoma viral oncogene homolog 2 | 0.08 | 5.2e-01 |
| 2099 | ESR1 | estrogen receptor 1 | -1.11 | 4.1e-03 |
| 2100 | ESR2 | estrogen receptor 2 (ER beta) | 0.13 | 4.9e-01 |
| 2288 | FKBP4 | FK506 binding protein 4, 59kDa | 0.83 | 6.8e-11 |
| 2289 | FKBP5 | FK506 binding protein 5 | -1.09 | 7.1e-08 |
| 23236 | PLCB1 | phospholipase C, beta 1 (phosphoinositide-specific) | 0.13 | 5.9e-01 |
| 2353 | FOS | FBJ murine osteosarcoma viral oncogene homolog | -2.84 | 1.5e-21 |
| 23533 | PIK3R5 | phosphoinositide-3-kinase, regulatory subunit 5 | 0.24 | 2.6e-01 |
| 2550 | GABBR1 | gamma-aminobutyric acid (GABA) B receptor, 1 | -2.15 | 1.1e-23 |
| 25759 | SHC2 | SHC (Src homology 2 domain containing) transforming protein 2 | -2.29 | 2.9e-26 |
| 2770 | GNAI1 | guanine nucleotide binding protein (G protein), alpha inhibiting activity polypeptide 1 | -0.76 | 1.6e-05 |
| 2771 | GNAI2 | guanine nucleotide binding protein (G protein), alpha inhibiting activity polypeptide 2 | -0.40 | 1.3e-04 |
| 2773 | GNAI3 | guanine nucleotide binding protein (G protein), alpha inhibiting activity polypeptide 3 | 0.15 | 6.1e-02 |
| 2775 | GNAO1 | guanine nucleotide binding protein (G protein), alpha activating activity polypeptide O | -3.23 | 6.2e-71 |
| 2776 | GNAQ | guanine nucleotide binding protein (G protein), q polypeptide | -0.64 | 8.9e-05 |
| 2778 | GNAS | GNAS complex locus | 0.76 | 5.9e-09 |
| 2852 | GPER1 | G protein-coupled estrogen receptor 1 | -1.39 | 1.1e-10 |
| 2885 | GRB2 | growth factor receptor-bound protein 2 | 0.00 | 9.9e-01 |
| 2911 | GRM1 | glutamate receptor, metabotropic 1 | -0.19 | 5.6e-01 |
| 3265 | HRAS | Harvey rat sarcoma viral oncogene homolog | 0.90 | 2.8e-08 |
| 3303 | HSPA1A | heat shock 70kDa protein 1A | -0.90 | 3.7e-05 |
| 3304 | HSPA1B | heat shock 70kDa protein 1B | 0.25 | 1.6e-01 |
| 3305 | HSPA1L | heat shock 70kDa protein 1-like | -0.94 | 3.9e-14 |
| 3306 | HSPA2 | heat shock 70kDa protein 2 | -0.90 | 6.3e-06 |
| 3310 | HSPA6 | heat shock 70kDa protein 6 (HSP70B') | 1.31 | 7.3e-05 |
| 3312 | HSPA8 | heat shock 70kDa protein 8 | 0.19 | 3.1e-01 |
| 3320 | HSP90AA1 | heat shock protein 90kDa alpha (cytosolic), class A member 1 | 0.63 | 2.4e-07 |
| 3326 | HSP90AB1 | heat shock protein 90kDa alpha (cytosolic), class B member 1 | 0.04 | 7.1e-01 |
| 3708 | ITPR1 | inositol 1,4,5-trisphosphate receptor, type 1 | -2.91 | 1.2e-76 |
| 3709 | ITPR2 | inositol 1,4,5-trisphosphate receptor, type 2 | -0.73 | 2.6e-05 |
| 3710 | ITPR3 | inositol 1,4,5-trisphosphate receptor, type 3 | 1.58 | 3.7e-18 |
| 3725 | JUN | jun proto-oncogene | -1.50 | 1.0e-16 |
| 3760 | KCNJ3 | potassium channel, inwardly rectifying subfamily J, member 3 | -1.17 | 3.8e-03 |
| 3762 | KCNJ5 | potassium channel, inwardly rectifying subfamily J, member 5 | -0.04 | 9.0e-01 |
| 3763 | KCNJ6 | potassium channel, inwardly rectifying subfamily J, member 6 | 1.22 | 4.4e-05 |
| 3765 | KCNJ9 | potassium channel, inwardly rectifying subfamily J, member 9 | 0.62 | 2.2e-02 |
| 3845 | KRAS | Kirsten rat sarcoma viral oncogene homolog | 0.33 | 1.3e-02 |
| 399694 | SHC4 | SHC (Src homology 2 domain containing) family, member 4 | -1.02 | 1.9e-05 |
| 4313 | MMP2 | matrix metallopeptidase 2 | -1.07 | 2.0e-04 |
| 4318 | MMP9 | matrix metallopeptidase 9 | 3.28 | 7.7e-09 |
| 468 | ATF4 | activating transcription factor 4 | 0.12 | 2.9e-01 |
| 4846 | NOS3 | nitric oxide synthase 3 (endothelial cell) | -0.71 | 6.4e-05 |
| 4893 | NRAS | neuroblastoma RAS viral (v-ras) oncogene homolog | 0.76 | 2.6e-07 |
| 4988 | OPRM1 | opioid receptor, mu 1 | 0.55 | 5.0e-02 |
| 51806 | CALML5 | calmodulin-like 5 | 2.63 | 2.8e-08 |
| 5290 | PIK3CA | phosphatidylinositol-4,5-bisphosphate 3-kinase, catalytic subunit alpha | -0.34 | 1.2e-02 |
| 5291 | PIK3CB | phosphatidylinositol-4,5-bisphosphate 3-kinase, catalytic subunit beta | 0.19 | 8.7e-02 |
| 5293 | PIK3CD | phosphatidylinositol-4,5-bisphosphate 3-kinase, catalytic subunit delta | -0.71 | 1.6e-06 |
| 5294 | PIK3CG | phosphatidylinositol-4,5-bisphosphate 3-kinase, catalytic subunit gamma | -0.98 | 4.5e-05 |
| 5295 | PIK3R1 | phosphoinositide-3-kinase, regulatory subunit 1 (alpha) | -0.80 | 2.1e-03 |
| 5296 | PIK3R2 | phosphoinositide-3-kinase, regulatory subunit 2 (beta) | 0.67 | 6.5e-08 |
| 5330 | PLCB2 | phospholipase C, beta 2 | 0.12 | 5.6e-01 |
| 5331 | PLCB3 | phospholipase C, beta 3 (phosphatidylinositol-specific) | 0.28 | 1.9e-02 |
| 5332 | PLCB4 | phospholipase C, beta 4 | 0.18 | 4.4e-01 |
| 53358 | SHC3 | SHC (Src homology 2 domain containing) transforming protein 3 | -1.66 | 1.2e-09 |
| 5566 | PRKACA | protein kinase, cAMP-dependent, catalytic, alpha | -0.17 | 1.0e-01 |
| 5567 | PRKACB | protein kinase, cAMP-dependent, catalytic, beta | -1.29 | 1.2e-22 |
| 5568 | PRKACG | protein kinase, cAMP-dependent, catalytic, gamma | 0.05 | 8.3e-01 |
| 5580 | PRKCD | protein kinase C, delta | 1.29 | 1.5e-16 |
| 5594 | MAPK1 | mitogen-activated protein kinase 1 | -0.24 | 2.6e-02 |
| 5595 | MAPK3 | mitogen-activated protein kinase 3 | -0.86 | 1.2e-13 |
| 5604 | MAP2K1 | mitogen-activated protein kinase kinase 1 | -0.05 | 6.3e-01 |
| 5605 | MAP2K2 | mitogen-activated protein kinase kinase 2 | 0.76 | 2.3e-06 |
| 5613 | PRKX | protein kinase, X-linked | 0.96 | 1.9e-08 |
| 5894 | RAF1 | Raf-1 proto-oncogene, serine/threonine kinase | -0.11 | 1.2e-01 |
| 6464 | SHC1 | SHC (Src homology 2 domain containing) transforming protein 1 | -0.10 | 3.4e-01 |
| 64764 | CREB3L2 | cAMP responsive element binding protein 3-like 2 | -1.17 | 5.7e-23 |
| 6654 | SOS1 | son of sevenless homolog 1 (Drosophila) | -0.44 | 1.5e-06 |
| 6655 | SOS2 | son of sevenless homolog 2 (Drosophila) | -0.83 | 1.9e-19 |
| 6667 | SP1 | Sp1 transcription factor | -0.38 | 9.5e-07 |
| 6714 | SRC | SRC proto-oncogene, non-receptor tyrosine kinase | 0.08 | 5.0e-01 |
| 7184 | HSP90B1 | heat shock protein 90kDa beta (Grp94), member 1 | 0.56 | 8.0e-06 |
| 801 | CALM1 | calmodulin 1 (phosphorylase kinase, delta) | 0.18 | 5.9e-02 |
| 805 | CALM2 | calmodulin 2 (phosphorylase kinase, delta) | -0.31 | 3.5e-04 |
| 808 | CALM3 | calmodulin 3 (phosphorylase kinase, delta) | -0.07 | 5.5e-01 |
| 810 | CALML3 | calmodulin-like 3 | 2.15 | 5.2e-04 |
| 84699 | CREB3L3 | cAMP responsive element binding protein 3-like 3 | 1.03 | 1.6e-03 |
| 8503 | PIK3R3 | phosphoinositide-3-kinase, regulatory subunit 3 (gamma) | 1.28 | 1.7e-10 |
| 90993 | CREB3L1 | cAMP responsive element binding protein 3-like 1 | -0.16 | 7.1e-01 |
| 9568 | GABBR2 | gamma-aminobutyric acid (GABA) B receptor, 2 | 0.15 | 7.0e-01 |
| 9586 | CREB5 | cAMP responsive element binding protein 5 | -0.25 | 2.4e-01 |

| ENTREZID | SYMBOL | GENENAME | FC | ADJ.PVAL |
| --- | --- | --- | --- | --- |

(Page generated on Tue Aug 25 12:04:32 2015 by ReportingTools 2.9.1 and hwriter 1.3.2)
