# Supplementary material for: Bioconductor’s EnrichmentBrowser: seamless navigation through combined results of set- & network-based enrichment analysis
Source: BMC Bioinformatics. 2016 Jan 20;17:45. doi: 10.1186/s12859-016-0884-1 (PMC4721010; doi:10.1186/s12859-016-0884-1)
Supplement: Supplementary file 3 — EnrichmentBrowser output (TCGA RNA-seq data). Unzip and open the contained index.html in the browser to view the contents of this file (tested with Firefox 39.0). (ZIP 7116.8 kb) [file 12859_2016_884_MOESM3_ESM.zip › hsa05200.html]

hsa05200: Gene Report


## hsa05200: Gene Report

| ENTREZID | SYMBOL | GENENAME | FC | ADJ.PVAL |
| --- | --- | --- | --- | --- |
| ENTREZID | SYMBOL | GENENAME | FC | ADJ.PVAL |
| 10000 | AKT3 | v-akt murine thymoma viral oncogene homolog 3 | -3.49 | 6.4e-108 |
| 1017 | CDK2 | cyclin-dependent kinase 2 | 0.55 | 2.3e-05 |
| 1019 | CDK4 | cyclin-dependent kinase 4 | 0.28 | 8.6e-03 |
| 1021 | CDK6 | cyclin-dependent kinase 6 | -0.93 | 6.5e-06 |
| 1026 | CDKN1A | cyclin-dependent kinase inhibitor 1A (p21, Cip1) | 0.08 | 7.6e-01 |
| 1027 | CDKN1B | cyclin-dependent kinase inhibitor 1B (p27, Kip1) | -1.42 | 1.6e-15 |
| 1029 | CDKN2A | cyclin-dependent kinase inhibitor 2A | 3.96 | 1.9e-09 |
| 10297 | APC2 | adenomatosis polyposis coli 2 | -0.95 | 7.8e-11 |
| 1030 | CDKN2B | cyclin-dependent kinase inhibitor 2B (p15, inhibits CDK4) | 1.13 | 5.8e-05 |
| 10319 | LAMC3 | laminin, gamma 3 | 0.32 | 3.3e-01 |
| 10342 | TFG | TRK-fused gene | 0.59 | 1.5e-15 |
| 1050 | CEBPA | CCAAT/enhancer binding protein (C/EBP), alpha | -0.12 | 6.7e-01 |
| 10928 | RALBP1 | ralA binding protein 1 | -0.08 | 5.4e-01 |
| 11186 | RASSF1 | Ras association (RalGDS/AF-6) domain family member 1 | -0.17 | 8.4e-02 |
| 11211 | FZD10 | frizzled class receptor 10 | 0.84 | 5.8e-02 |
| 112398 | EGLN2 | egl-9 family hypoxia-inducible factor 2 | -1.22 | 9.2e-08 |
| 112399 | EGLN3 | egl-9 family hypoxia-inducible factor 3 | 1.54 | 1.8e-07 |
| 112401 | BIRC8 | baculoviral IAP repeat containing 8 | 0.08 | 8.0e-01 |
| 1147 | CHUK | conserved helix-loop-helix ubiquitous kinase | 0.13 | 1.9e-01 |
| 1163 | CKS1B | CDC28 protein kinase regulatory subunit 1B | 1.56 | 1.7e-12 |
| 1164 | CKS2 | CDC28 protein kinase regulatory subunit 2 | 2.80 | 8.5e-32 |
| 1282 | COL4A1 | collagen, type IV, alpha 1 | 0.34 | 5.3e-02 |
| 1284 | COL4A2 | collagen, type IV, alpha 2 | -0.18 | 2.7e-01 |
| 1285 | COL4A3 | collagen, type IV, alpha 3 (Goodpasture antigen) | -1.39 | 1.7e-05 |
| 1286 | COL4A4 | collagen, type IV, alpha 4 | -1.52 | 9.2e-08 |
| 1287 | COL4A5 | collagen, type IV, alpha 5 | -1.28 | 1.7e-06 |
| 1288 | COL4A6 | collagen, type IV, alpha 6 | -4.75 | 4.7e-103 |
| 1387 | CREBBP | CREB binding protein | -0.60 | 2.6e-08 |
| 1398 | CRK | v-crk avian sarcoma virus CT10 oncogene homolog | -0.22 | 1.6e-02 |
| 1399 | CRKL | v-crk avian sarcoma virus CT10 oncogene homolog-like | -0.33 | 2.1e-04 |
| 1436 | CSF1R | colony stimulating factor 1 receptor | -0.21 | 3.4e-01 |
| 1438 | CSF2RA | colony stimulating factor 2 receptor, alpha, low-affinity (granulocyte-macrophage) | -0.34 | 1.7e-01 |
| 1441 | CSF3R | colony stimulating factor 3 receptor (granulocyte) | 0.56 | 5.5e-02 |
| 1487 | CTBP1 | C-terminal binding protein 1 | 0.30 | 2.3e-03 |
| 1488 | CTBP2 | C-terminal binding protein 2 | 0.28 | 1.6e-02 |
| 1495 | CTNNA1 | catenin (cadherin-associated protein), alpha 1, 102kDa | 0.45 | 1.3e-06 |
| 1496 | CTNNA2 | catenin (cadherin-associated protein), alpha 2 | 0.73 | 2.4e-01 |
| 1499 | CTNNB1 | catenin (cadherin-associated protein), beta 1, 88kDa | -0.40 | 1.2e-03 |
| 1612 | DAPK1 | death-associated protein kinase 1 | -0.33 | 8.8e-02 |
| 1613 | DAPK3 | death-associated protein kinase 3 | -0.45 | 2.6e-04 |
| 1630 | DCC | DCC netrin 1 receptor | -0.48 | 1.8e-01 |
| 1855 | DVL1 | dishevelled segment polarity protein 1 | 0.22 | 8.9e-02 |
| 1856 | DVL2 | dishevelled segment polarity protein 2 | -0.46 | 4.4e-06 |
| 1857 | DVL3 | dishevelled segment polarity protein 3 | 0.28 | 2.6e-03 |
| 1869 | E2F1 | E2F transcription factor 1 | 2.99 | 8.8e-21 |
| 1870 | E2F2 | E2F transcription factor 2 | 4.05 | 2.8e-42 |
| 1871 | E2F3 | E2F transcription factor 3 | 1.47 | 5.0e-26 |
| 1950 | EGF | epidermal growth factor | 0.15 | 7.1e-01 |
| 1956 | EGFR | epidermal growth factor receptor | -0.93 | 2.7e-06 |
| 2033 | EP300 | E1A binding protein p300 | -0.20 | 1.2e-01 |
| 2034 | EPAS1 | endothelial PAS domain protein 1 | -1.26 | 1.5e-13 |
| 2064 | ERBB2 | erb-b2 receptor tyrosine kinase 2 | 0.85 | 3.8e-05 |
| 207 | AKT1 | v-akt murine thymoma viral oncogene homolog 1 | 0.54 | 4.8e-09 |
| 208 | AKT2 | v-akt murine thymoma viral oncogene homolog 2 | 0.08 | 5.2e-01 |
| 2113 | ETS1 | v-ets avian erythroblastosis virus E26 oncogene homolog 1 | -1.14 | 2.2e-15 |
| 2122 | MECOM | MDS1 and EVI1 complex locus | 1.46 | 6.4e-12 |
| 2246 | FGF1 | fibroblast growth factor 1 (acidic) | -0.78 | 1.3e-03 |
| 2247 | FGF2 | fibroblast growth factor 2 (basic) | -4.15 | 3.0e-112 |
| 2248 | FGF3 | fibroblast growth factor 3 | 2.60 | 2.3e-06 |
| 2249 | FGF4 | fibroblast growth factor 4 | 1.10 | 2.0e-03 |
| 2250 | FGF5 | fibroblast growth factor 5 | -0.54 | 9.0e-02 |
| 2251 | FGF6 | fibroblast growth factor 6 | 0.49 | 5.7e-03 |
| 2252 | FGF7 | fibroblast growth factor 7 | -5.41 | 2.5e-100 |
| 2253 | FGF8 | fibroblast growth factor 8 (androgen-induced) | 2.40 | 1.6e-08 |
| 2254 | FGF9 | fibroblast growth factor 9 | -0.01 | 9.9e-01 |
| 2255 | FGF10 | fibroblast growth factor 10 | -4.32 | 1.7e-35 |
| 2256 | FGF11 | fibroblast growth factor 11 | 1.08 | 5.7e-05 |
| 2257 | FGF12 | fibroblast growth factor 12 | -1.29 | 1.0e-04 |
| 2258 | FGF13 | fibroblast growth factor 13 | -2.47 | 1.7e-25 |
| 2259 | FGF14 | fibroblast growth factor 14 | -2.09 | 2.2e-06 |
| 2260 | FGFR1 | fibroblast growth factor receptor 1 | -0.24 | 2.4e-01 |
| 2261 | FGFR3 | fibroblast growth factor receptor 3 | 0.59 | 1.4e-01 |
| 2263 | FGFR2 | fibroblast growth factor receptor 2 | -0.60 | 3.5e-02 |
| 2271 | FH | fumarate hydratase | 0.85 | 3.0e-14 |
| 2277 | FIGF | c-fos induced growth factor (vascular endothelial growth factor D) | -2.88 | 3.5e-25 |
| 22798 | LAMB4 | laminin, beta 4 | 1.39 | 2.8e-08 |
| 2308 | FOXO1 | forkhead box O1 | -1.67 | 4.7e-33 |
| 2322 | FLT3 | fms-related tyrosine kinase 3 | -0.83 | 9.5e-03 |
| 2323 | FLT3LG | fms-related tyrosine kinase 3 ligand | -1.30 | 4.8e-21 |
| 2335 | FN1 | fibronectin 1 | -0.92 | 9.5e-04 |
| 2353 | FOS | FBJ murine osteosarcoma viral oncogene homolog | -2.84 | 1.5e-21 |
| 23533 | PIK3R5 | phosphoinositide-3-kinase, regulatory subunit 5 | 0.24 | 2.6e-01 |
| 23604 | DAPK2 | death-associated protein kinase 2 | 0.73 | 1.5e-03 |
| 23624 | CBLC | Cbl proto-oncogene C, E3 ubiquitin protein ligase | 3.28 | 7.8e-13 |
| 2475 | MTOR | mechanistic target of rapamycin (serine/threonine kinase) | 0.74 | 9.0e-12 |
| 25 | ABL1 | ABL proto-oncogene 1, non-receptor tyrosine kinase | -1.00 | 8.4e-22 |
| 2535 | FZD2 | frizzled class receptor 2 | 0.64 | 2.8e-03 |
| 26060 | APPL1 | adaptor protein, phosphotyrosine interaction, PH domain and leucine zipper containing 1 | -0.61 | 3.3e-08 |
| 26281 | FGF20 | fibroblast growth factor 20 | 0.85 | 1.6e-01 |
| 26291 | FGF21 | fibroblast growth factor 21 | 1.54 | 1.8e-07 |
| 27006 | FGF22 | fibroblast growth factor 22 | -0.63 | 3.7e-02 |
| 27148 | STK36 | serine/threonine kinase 36 | 0.86 | 1.2e-08 |
| 2735 | GLI1 | GLI family zinc finger 1 | -2.56 | 2.4e-24 |
| 2736 | GLI2 | GLI family zinc finger 2 | -3.14 | 3.3e-63 |
| 2737 | GLI3 | GLI family zinc finger 3 | -2.91 | 1.7e-49 |
| 284217 | LAMA1 | laminin, alpha 1 | 2.48 | 1.6e-04 |
| 2885 | GRB2 | growth factor receptor-bound protein 2 | 0.00 | 9.9e-01 |
| 29119 | CTNNA3 | catenin (cadherin-associated protein), alpha 3 | -3.33 | 1.8e-24 |
| 2932 | GSK3B | glycogen synthase kinase 3 beta | 0.22 | 2.5e-02 |
| 2950 | GSTP1 | glutathione S-transferase pi 1 | 1.21 | 5.8e-15 |
| 2956 | MSH6 | mutS homolog 6 | -0.43 | 4.6e-04 |
| 3065 | HDAC1 | histone deacetylase 1 | 0.41 | 8.5e-07 |
| 3066 | HDAC2 | histone deacetylase 2 | 0.46 | 4.6e-05 |
| 3082 | HGF | hepatocyte growth factor (hepapoietin A; scatter factor) | -2.52 | 1.1e-26 |
| 3091 | HIF1A | hypoxia inducible factor 1, alpha subunit (basic helix-loop-helix transcription factor) | 0.28 | 2.5e-01 |
| 324 | APC | adenomatous polyposis coli | -0.50 | 2.4e-06 |
| 3265 | HRAS | Harvey rat sarcoma viral oncogene homolog | 0.90 | 2.8e-08 |
| 329 | BIRC2 | baculoviral IAP repeat containing 2 | -0.28 | 6.0e-03 |
| 330 | BIRC3 | baculoviral IAP repeat containing 3 | -0.30 | 2.7e-01 |
| 331 | XIAP | X-linked inhibitor of apoptosis, E3 ubiquitin protein ligase | -0.20 | 4.1e-02 |
| 332 | BIRC5 | baculoviral IAP repeat containing 5 | 4.77 | 1.5e-33 |
| 3320 | HSP90AA1 | heat shock protein 90kDa alpha (cytosolic), class A member 1 | 0.63 | 2.4e-07 |
| 3326 | HSP90AB1 | heat shock protein 90kDa alpha (cytosolic), class B member 1 | 0.04 | 7.1e-01 |
| 3479 | IGF1 | insulin-like growth factor 1 (somatomedin C) | -2.10 | 3.2e-15 |
| 3480 | IGF1R | insulin-like growth factor 1 receptor | -0.97 | 2.1e-05 |
| 354 | KLK3 | kallikrein-related peptidase 3 | -0.43 | 4.0e-01 |
| 355 | FAS | Fas cell surface death receptor | -1.02 | 8.0e-12 |
| 3551 | IKBKB | inhibitor of kappa light polypeptide gene enhancer in B-cells, kinase beta | -0.27 | 2.9e-03 |
| 356 | FASLG | Fas ligand (TNF superfamily, member 6) | -0.18 | 6.0e-01 |
| 3569 | IL6 | interleukin 6 | -0.95 | 5.1e-03 |
| 3576 | CXCL8 | chemokine (C-X-C motif) ligand 8 | 2.08 | 4.3e-06 |
| 3655 | ITGA6 | integrin, alpha 6 | -0.43 | 6.6e-03 |
| 367 | AR | androgen receptor | -2.35 | 1.9e-16 |
| 3673 | ITGA2 | integrin, alpha 2 (CD49B, alpha 2 subunit of VLA-2 receptor) | -0.51 | 4.4e-02 |
| 3674 | ITGA2B | integrin, alpha 2b (platelet glycoprotein IIb of IIb/IIIa complex, antigen CD41) | 0.20 | 4.9e-01 |
| 3675 | ITGA3 | integrin, alpha 3 (antigen CD49C, alpha 3 subunit of VLA-3 receptor) | -0.07 | 7.9e-01 |
| 3685 | ITGAV | integrin, alpha V | -0.98 | 3.2e-09 |
| 3688 | ITGB1 | integrin, beta 1 (fibronectin receptor, beta polypeptide, antigen CD29 includes MDF2, MSK12) | -0.74 | 1.8e-06 |
| 369 | ARAF | A-Raf proto-oncogene, serine/threonine kinase | -0.12 | 2.8e-01 |
| 3716 | JAK1 | Janus kinase 1 | -1.04 | 5.8e-14 |
| 3725 | JUN | jun proto-oncogene | -1.50 | 1.0e-16 |
| 3728 | JUP | junction plakoglobin | 0.89 | 8.9e-09 |
| 3815 | KIT | v-kit Hardy-Zuckerman 4 feline sarcoma viral oncogene homolog | -1.69 | 1.4e-12 |
| 3845 | KRAS | Kirsten rat sarcoma viral oncogene homolog | 0.33 | 1.3e-02 |
| 387 | RHOA | ras homolog family member A | -0.24 | 2.2e-03 |
| 3908 | LAMA2 | laminin, alpha 2 | -2.77 | 6.3e-46 |
| 3909 | LAMA3 | laminin, alpha 3 | -0.38 | 1.6e-01 |
| 3910 | LAMA4 | laminin, alpha 4 | -2.53 | 8.3e-51 |
| 3911 | LAMA5 | laminin, alpha 5 | 0.91 | 1.8e-07 |
| 3912 | LAMB1 | laminin, beta 1 | 0.12 | 5.8e-01 |
| 3913 | LAMB2 | laminin, beta 2 (laminin S) | -0.77 | 2.5e-07 |
| 3914 | LAMB3 | laminin, beta 3 | 2.35 | 7.8e-08 |
| 3915 | LAMC1 | laminin, gamma 1 (formerly LAMB2) | 0.27 | 1.2e-01 |
| 3918 | LAMC2 | laminin, gamma 2 | 1.66 | 5.5e-08 |
| 405 | ARNT | aryl hydrocarbon receptor nuclear translocator | -0.69 | 5.5e-17 |
| 4087 | SMAD2 | SMAD family member 2 | -0.18 | 4.8e-02 |
| 4088 | SMAD3 | SMAD family member 3 | -1.19 | 3.7e-21 |
| 4089 | SMAD4 | SMAD family member 4 | -0.86 | 4.9e-19 |
| 4149 | MAX | MYC associated factor X | -0.61 | 1.3e-20 |
| 4193 | MDM2 | MDM2 proto-oncogene, E3 ubiquitin protein ligase | 0.32 | 3.8e-02 |
| 4233 | MET | MET proto-oncogene, receptor tyrosine kinase | 0.68 | 2.2e-02 |
| 4254 | KITLG | KIT ligand | -1.48 | 1.5e-12 |
| 4286 | MITF | microphthalmia-associated transcription factor | -2.23 | 2.1e-42 |
| 4292 | MLH1 | mutL homolog 1 | -0.91 | 5.8e-05 |
| 4312 | MMP1 | matrix metallopeptidase 1 | 4.53 | 2.3e-23 |
| 4313 | MMP2 | matrix metallopeptidase 2 | -1.07 | 2.0e-04 |
| 4318 | MMP9 | matrix metallopeptidase 9 | 3.28 | 7.7e-09 |
| 4436 | MSH2 | mutS homolog 2 | 0.85 | 1.1e-09 |
| 4437 | MSH3 | mutS homolog 3 | -0.12 | 2.5e-01 |
| 4609 | MYC | v-myc avian myelocytomatosis viral oncogene homolog | -1.35 | 1.7e-10 |
| 4790 | NFKB1 | nuclear factor of kappa light polypeptide gene enhancer in B-cells 1 | -0.70 | 5.4e-11 |
| 4791 | NFKB2 | nuclear factor of kappa light polypeptide gene enhancer in B-cells 2 (p49/p100) | 0.40 | 3.8e-03 |
| 4792 | NFKBIA | nuclear factor of kappa light polypeptide gene enhancer in B-cells inhibitor, alpha | -0.36 | 7.9e-03 |
| 4824 | NKX3-1 | NK3 homeobox 1 | -0.87 | 5.0e-04 |
| 4843 | NOS2 | nitric oxide synthase 2, inducible | -1.14 | 4.1e-04 |
| 4893 | NRAS | neuroblastoma RAS viral (v-ras) oncogene homolog | 0.76 | 2.6e-07 |
| 4914 | NTRK1 | neurotrophic tyrosine kinase, receptor, type 1 | -0.24 | 4.5e-01 |
| 51176 | LEF1 | lymphoid enhancer-binding factor 1 | 0.34 | 3.4e-01 |
| 51384 | WNT16 | wingless-type MMTV integration site family, member 16 | 0.22 | 6.2e-01 |
| 5154 | PDGFA | platelet-derived growth factor alpha polypeptide | -0.37 | 9.1e-02 |
| 5155 | PDGFB | platelet-derived growth factor beta polypeptide | -0.41 | 7.9e-03 |
| 5156 | PDGFRA | platelet-derived growth factor receptor, alpha polypeptide | -3.00 | 7.1e-35 |
| 5159 | PDGFRB | platelet-derived growth factor receptor, beta polypeptide | -1.85 | 6.8e-23 |
| 51684 | SUFU | suppressor of fused homolog (Drosophila) | -0.33 | 1.4e-03 |
| 5228 | PGF | placental growth factor | -0.82 | 3.6e-05 |
| 5290 | PIK3CA | phosphatidylinositol-4,5-bisphosphate 3-kinase, catalytic subunit alpha | -0.34 | 1.2e-02 |
| 5291 | PIK3CB | phosphatidylinositol-4,5-bisphosphate 3-kinase, catalytic subunit beta | 0.19 | 8.7e-02 |
| 5293 | PIK3CD | phosphatidylinositol-4,5-bisphosphate 3-kinase, catalytic subunit delta | -0.71 | 1.6e-06 |
| 5294 | PIK3CG | phosphatidylinositol-4,5-bisphosphate 3-kinase, catalytic subunit gamma | -0.98 | 4.5e-05 |
| 5295 | PIK3R1 | phosphoinositide-3-kinase, regulatory subunit 1 (alpha) | -0.80 | 2.1e-03 |
| 5296 | PIK3R2 | phosphoinositide-3-kinase, regulatory subunit 2 (beta) | 0.67 | 6.5e-08 |
| 5335 | PLCG1 | phospholipase C, gamma 1 | -0.02 | 9.1e-01 |
| 5336 | PLCG2 | phospholipase C, gamma 2 (phosphatidylinositol-specific) | -0.70 | 1.7e-05 |
| 5337 | PLD1 | phospholipase D1, phosphatidylcholine-specific | -0.95 | 1.4e-13 |
| 5371 | PML | promyelocytic leukemia | -0.05 | 7.3e-01 |
| 54205 | CYCS | cytochrome c, somatic | 0.47 | 1.4e-03 |
| 54361 | WNT4 | wingless-type MMTV integration site family, member 4 | -2.70 | 5.9e-33 |
| 54583 | EGLN1 | egl-9 family hypoxia-inducible factor 1 | -0.29 | 9.4e-03 |
| 5467 | PPARD | peroxisome proliferator-activated receptor delta | 0.41 | 5.4e-04 |
| 5468 | PPARG | peroxisome proliferator-activated receptor gamma | -0.63 | 4.6e-02 |
| 5578 | PRKCA | protein kinase C, alpha | -1.99 | 8.5e-30 |
| 5579 | PRKCB | protein kinase C, beta | -1.76 | 5.6e-18 |
| 5582 | PRKCG | protein kinase C, gamma | 0.28 | 5.7e-01 |
| 5594 | MAPK1 | mitogen-activated protein kinase 1 | -0.24 | 2.6e-02 |
| 5595 | MAPK3 | mitogen-activated protein kinase 3 | -0.86 | 1.2e-13 |
| 5599 | MAPK8 | mitogen-activated protein kinase 8 | 0.06 | 7.3e-01 |
| 5601 | MAPK9 | mitogen-activated protein kinase 9 | -0.12 | 1.0e-01 |
| 5602 | MAPK10 | mitogen-activated protein kinase 10 | -2.79 | 1.3e-41 |
| 5604 | MAP2K1 | mitogen-activated protein kinase kinase 1 | -0.05 | 6.3e-01 |
| 5605 | MAP2K2 | mitogen-activated protein kinase kinase 2 | 0.76 | 2.3e-06 |
| 572 | BAD | BCL2-associated agonist of cell death | 0.17 | 2.1e-01 |
| 5727 | PTCH1 | patched 1 | -1.17 | 9.4e-14 |
| 5728 | PTEN | phosphatase and tensin homolog | -0.86 | 7.4e-09 |
| 5743 | PTGS2 | prostaglandin-endoperoxide synthase 2 (prostaglandin G/H synthase and cyclooxygenase) | -1.00 | 7.9e-03 |
| 5747 | PTK2 | protein tyrosine kinase 2 | -0.01 | 9.3e-01 |
| 581 | BAX | BCL2-associated X protein | 0.89 | 9.4e-11 |
| 5879 | RAC1 | ras-related C3 botulinum toxin substrate 1 (rho family, small GTP binding protein Rac1) | 0.02 | 7.8e-01 |
| 5880 | RAC2 | ras-related C3 botulinum toxin substrate 2 (rho family, small GTP binding protein Rac2) | 0.34 | 1.5e-01 |
| 5881 | RAC3 | ras-related C3 botulinum toxin substrate 3 (rho family, small GTP binding protein Rac3) | 2.81 | 6.5e-19 |
| 5888 | RAD51 | RAD51 recombinase | 3.07 | 2.2e-35 |
| 5894 | RAF1 | Raf-1 proto-oncogene, serine/threonine kinase | -0.11 | 1.2e-01 |
| 5898 | RALA | v-ral simian leukemia viral oncogene homolog A (ras related) | 0.25 | 3.2e-03 |
| 5899 | RALB | v-ral simian leukemia viral oncogene homolog B | -0.53 | 9.8e-10 |
| 5900 | RALGDS | ral guanine nucleotide dissociation stimulator | 0.07 | 5.3e-01 |
| 5914 | RARA | retinoic acid receptor, alpha | -0.35 | 1.5e-02 |
| 5915 | RARB | retinoic acid receptor, beta | -1.21 | 9.8e-13 |
| 5925 | RB1 | retinoblastoma 1 | -0.09 | 6.1e-01 |
| 595 | CCND1 | cyclin D1 | 0.84 | 4.4e-04 |
| 596 | BCL2 | B-cell CLL/lymphoma 2 | -2.24 | 1.7e-33 |
| 5970 | RELA | v-rel avian reticuloendotheliosis viral oncogene homolog A | 0.00 | 9.6e-01 |
| 5979 | RET | ret proto-oncogene | -0.51 | 1.8e-01 |
| 598 | BCL2L1 | BCL2-like 1 | 0.66 | 6.2e-09 |
| 613 | BCR | breakpoint cluster region | 0.76 | 1.3e-11 |
| 6256 | RXRA | retinoid X receptor, alpha | -0.86 | 1.4e-12 |
| 6257 | RXRB | retinoid X receptor, beta | -0.45 | 7.7e-09 |
| 6258 | RXRG | retinoid X receptor, gamma | -1.52 | 7.0e-05 |
| 637 | BID | BH3 interacting domain death agonist | 0.92 | 2.4e-14 |
| 64399 | HHIP | hedgehog interacting protein | 0.18 | 7.0e-01 |
| 6469 | SHH | sonic hedgehog | 1.96 | 9.6e-06 |
| 650 | BMP2 | bone morphogenetic protein 2 | -1.25 | 1.7e-05 |
| 6502 | SKP2 | S-phase kinase-associated protein 2, E3 ubiquitin protein ligase | 1.04 | 3.9e-10 |
| 6513 | SLC2A1 | solute carrier family 2 (facilitated glucose transporter), member 1 | 1.81 | 9.6e-17 |
| 652 | BMP4 | bone morphogenetic protein 4 | -1.09 | 2.0e-04 |
| 6608 | SMO | smoothened, frizzled class receptor | -0.47 | 8.7e-03 |
| 6654 | SOS1 | son of sevenless homolog 1 (Drosophila) | -0.44 | 1.5e-06 |
| 6655 | SOS2 | son of sevenless homolog 2 (Drosophila) | -0.83 | 1.9e-19 |
| 6688 | SPI1 | Spi-1 proto-oncogene | 0.82 | 7.3e-04 |
| 673 | BRAF | B-Raf proto-oncogene, serine/threonine kinase | 0.82 | 1.7e-09 |
| 675 | BRCA2 | breast cancer 2, early onset | 1.68 | 5.2e-11 |
| 6772 | STAT1 | signal transducer and activator of transcription 1, 91kDa | 0.96 | 1.2e-06 |
| 6774 | STAT3 | signal transducer and activator of transcription 3 (acute-phase response factor) | -0.02 | 8.3e-01 |
| 6776 | STAT5A | signal transducer and activator of transcription 5A | -1.03 | 2.9e-16 |
| 6777 | STAT5B | signal transducer and activator of transcription 5B | -1.83 | 7.5e-74 |
| 6789 | STK4 | serine/threonine kinase 4 | 0.07 | 4.8e-01 |
| 6921 | TCEB1 | transcription elongation factor B (SIII), polypeptide 1 (15kDa, elongin C) | 0.74 | 2.0e-12 |
| 6923 | TCEB2 | transcription elongation factor B (SIII), polypeptide 2 (18kDa, elongin B) | 0.71 | 1.5e-05 |
| 6932 | TCF7 | transcription factor 7 (T-cell specific, HMG-box) | 0.60 | 1.7e-02 |
| 6934 | TCF7L2 | transcription factor 7-like 2 (T-cell specific, HMG-box) | -1.19 | 3.2e-13 |
| 7039 | TGFA | transforming growth factor, alpha | 1.93 | 2.4e-07 |
| 7040 | TGFB1 | transforming growth factor, beta 1 | -0.65 | 7.3e-05 |
| 7042 | TGFB2 | transforming growth factor, beta 2 | -1.33 | 1.4e-08 |
| 7043 | TGFB3 | transforming growth factor, beta 3 | -1.52 | 1.4e-25 |
| 7046 | TGFBR1 | transforming growth factor, beta receptor 1 | -0.62 | 1.0e-09 |
| 7048 | TGFBR2 | transforming growth factor, beta receptor II (70/80kDa) | -1.75 | 6.2e-30 |
| 7157 | TP53 | tumor protein p53 | 0.53 | 2.8e-03 |
| 7170 | TPM3 | tropomyosin 3 | 1.05 | 7.7e-27 |
| 7175 | TPR | translocated promoter region, nuclear basket protein | 0.16 | 1.3e-01 |
| 7184 | HSP90B1 | heat shock protein 90kDa beta (Grp94), member 1 | 0.56 | 8.0e-06 |
| 7185 | TRAF1 | TNF receptor-associated factor 1 | -0.33 | 2.6e-02 |
| 7186 | TRAF2 | TNF receptor-associated factor 2 | 0.83 | 3.1e-11 |
| 7187 | TRAF3 | TNF receptor-associated factor 3 | -0.04 | 6.8e-01 |
| 7188 | TRAF5 | TNF receptor-associated factor 5 | -0.48 | 5.3e-03 |
| 7189 | TRAF6 | TNF receptor-associated factor 6, E3 ubiquitin protein ligase | -0.55 | 1.3e-13 |
| 7422 | VEGFA | vascular endothelial growth factor A | 0.57 | 2.4e-03 |
| 7423 | VEGFB | vascular endothelial growth factor B | -0.13 | 3.0e-01 |
| 7424 | VEGFC | vascular endothelial growth factor C | -1.46 | 1.3e-20 |
| 7428 | VHL | von Hippel-Lindau tumor suppressor, E3 ubiquitin protein ligase | 0.42 | 4.0e-05 |
| 7471 | WNT1 | wingless-type MMTV integration site family, member 1 | 0.17 | 6.0e-01 |
| 7472 | WNT2 | wingless-type MMTV integration site family member 2 | -1.34 | 9.0e-07 |
| 7473 | WNT3 | wingless-type MMTV integration site family, member 3 | -0.63 | 5.6e-02 |
| 7474 | WNT5A | wingless-type MMTV integration site family, member 5A | -0.69 | 2.8e-03 |
| 7475 | WNT6 | wingless-type MMTV integration site family, member 6 | -0.58 | 1.4e-01 |
| 7476 | WNT7A | wingless-type MMTV integration site family, member 7A | 2.85 | 4.4e-04 |
| 7477 | WNT7B | wingless-type MMTV integration site family, member 7B | 2.71 | 8.1e-09 |
| 7478 | WNT8A | wingless-type MMTV integration site family, member 8A | -0.23 | 4.1e-01 |
| 7479 | WNT8B | wingless-type MMTV integration site family, member 8B | 0.90 | 3.9e-04 |
| 7480 | WNT10B | wingless-type MMTV integration site family, member 10B | 0.98 | 1.0e-03 |
| 7481 | WNT11 | wingless-type MMTV integration site family, member 11 | 0.69 | 1.4e-01 |
| 7482 | WNT2B | wingless-type MMTV integration site family, member 2B | -2.35 | 4.4e-27 |
| 7483 | WNT9A | wingless-type MMTV integration site family, member 9A | -1.58 | 1.0e-08 |
| 7484 | WNT9B | wingless-type MMTV integration site family, member 9B | -1.70 | 5.1e-08 |
| 7704 | ZBTB16 | zinc finger and BTB domain containing 16 | -5.37 | 8.8e-71 |
| 7849 | PAX8 | paired box 8 | 2.54 | 7.6e-12 |
| 7855 | FZD5 | frizzled class receptor 5 | 1.83 | 5.8e-12 |
| 79444 | BIRC7 | baculoviral IAP repeat containing 7 | 1.99 | 1.7e-08 |
| 7976 | FZD3 | frizzled class receptor 3 | 0.58 | 6.3e-04 |
| 8030 | CCDC6 | coiled-coil domain containing 6 | 0.58 | 7.1e-04 |
| 8031 | NCOA4 | nuclear receptor coactivator 4 | -0.40 | 2.1e-04 |
| 80326 | WNT10A | wingless-type MMTV integration site family, member 10A | 2.18 | 1.7e-05 |
| 8074 | FGF23 | fibroblast growth factor 23 | 0.55 | 7.2e-02 |
| 81029 | WNT5B | wingless-type MMTV integration site family, member 5B | -1.32 | 1.8e-06 |
| 8312 | AXIN1 | axin 1 | 0.59 | 1.3e-08 |
| 8313 | AXIN2 | axin 2 | -1.38 | 6.3e-09 |
| 8321 | FZD1 | frizzled class receptor 1 | -0.73 | 6.8e-06 |
| 8322 | FZD4 | frizzled class receptor 4 | -1.99 | 6.1e-54 |
| 8323 | FZD6 | frizzled class receptor 6 | 0.27 | 1.6e-01 |
| 8324 | FZD7 | frizzled class receptor 7 | -1.85 | 7.1e-15 |
| 8325 | FZD8 | frizzled class receptor 8 | 0.84 | 1.0e-02 |
| 8326 | FZD9 | frizzled class receptor 9 | 0.73 | 7.6e-03 |
| 83439 | TCF7L1 | transcription factor 7-like 1 (T-cell specific, HMG-box) | 0.00 | 9.9e-01 |
| 83593 | RASSF5 | Ras association (RalGDS/AF-6) domain family member 5 | -0.46 | 6.9e-03 |
| 836 | CASP3 | caspase 3, apoptosis-related cysteine peptidase | 0.88 | 6.8e-18 |
| 841 | CASP8 | caspase 8, apoptosis-related cysteine peptidase | 0.30 | 1.2e-02 |
| 842 | CASP9 | caspase 9, apoptosis-related cysteine peptidase | -0.14 | 1.2e-01 |
| 8453 | CUL2 | cullin 2 | 0.21 | 1.2e-02 |
| 8503 | PIK3R3 | phosphoinositide-3-kinase, regulatory subunit 3 (gamma) | 1.28 | 1.7e-10 |
| 8517 | IKBKG | inhibitor of kappa light polypeptide gene enhancer in B-cells, kinase gamma | -0.37 | 1.1e-04 |
| 861 | RUNX1 | runt-related transcription factor 1 | 1.56 | 2.5e-12 |
| 862 | RUNX1T1 | runt-related transcription factor 1; translocated to, 1 (cyclin D-related) | -3.69 | 2.4e-81 |
| 8643 | PTCH2 | patched 2 | -1.56 | 1.7e-18 |
| 867 | CBL | Cbl proto-oncogene, E3 ubiquitin protein ligase | -0.47 | 4.7e-05 |
| 868 | CBLB | Cbl proto-oncogene B, E3 ubiquitin protein ligase | 0.30 | 1.1e-02 |
| 8772 | FADD | Fas (TNFRSF6)-associated via death domain | 0.40 | 9.8e-06 |
| 8817 | FGF18 | fibroblast growth factor 18 | 2.72 | 2.5e-08 |
| 8822 | FGF17 | fibroblast growth factor 17 | -0.16 | 6.9e-01 |
| 8823 | FGF16 | fibroblast growth factor 16 | -0.66 | 1.0e-03 |
| 8900 | CCNA1 | cyclin A1 | 2.03 | 1.5e-03 |
| 89780 | WNT3A | wingless-type MMTV integration site family, member 3A | 1.40 | 1.3e-04 |
| 898 | CCNE1 | cyclin E1 | 3.74 | 4.9e-19 |
| 9063 | PIAS2 | protein inhibitor of activated STAT, 2 | 0.35 | 2.8e-03 |
| 9134 | CCNE2 | cyclin E2 | 2.01 | 6.1e-12 |
| 9618 | TRAF4 | TNF receptor-associated factor 4 | 2.41 | 2.0e-54 |
| 9915 | ARNT2 | aryl-hydrocarbon receptor nuclear translocator 2 | 0.21 | 3.7e-01 |
| 9965 | FGF19 | fibroblast growth factor 19 | 1.90 | 9.0e-04 |
| 9978 | RBX1 | ring-box 1, E3 ubiquitin protein ligase | 0.54 | 1.3e-07 |
| 998 | CDC42 | cell division cycle 42 | -0.03 | 8.1e-01 |
| 999 | CDH1 | cadherin 1, type 1, E-cadherin (epithelial) | 2.87 | 3.3e-31 |

| ENTREZID | SYMBOL | GENENAME | FC | ADJ.PVAL |
| --- | --- | --- | --- | --- |

(Page generated on Mon Aug 24 22:02:41 2015 by ReportingTools 2.9.1 and hwriter 1.3.2)
