# Supplementary material for: Bioconductor’s EnrichmentBrowser: seamless navigation through combined results of set- & network-based enrichment analysis
Source: BMC Bioinformatics. 2016 Jan 20;17:45. doi: 10.1186/s12859-016-0884-1 (PMC4721010; doi:10.1186/s12859-016-0884-1)
Supplement: Supplementary file 3 — EnrichmentBrowser output (TCGA RNA-seq data). Unzip and open the contained index.html in the browser to view the contents of this file (tested with Firefox 39.0). (ZIP 7116.8 kb) [file 12859_2016_884_MOESM3_ESM.zip › hsa05205.html]

hsa05205: Gene Report


## hsa05205: Gene Report

| ENTREZID | SYMBOL | GENENAME | FC | ADJ.PVAL |
| --- | --- | --- | --- | --- |
| ENTREZID | SYMBOL | GENENAME | FC | ADJ.PVAL |
| 10000 | AKT3 | v-akt murine thymoma viral oncogene homolog 3 | -3.49 | 6.4e-108 |
| 1026 | CDKN1A | cyclin-dependent kinase inhibitor 1A (p21, Cip1) | 0.08 | 7.6e-01 |
| 10818 | FRS2 | fibroblast growth factor receptor substrate 2 | -0.64 | 9.2e-11 |
| 10855 | HPSE | heparanase | -1.28 | 3.9e-12 |
| 11211 | FZD10 | frizzled class receptor 10 | 0.84 | 5.8e-02 |
| 117581 | TWIST2 | twist family bHLH transcription factor 2 | -3.23 | 1.1e-31 |
| 1432 | MAPK14 | mitogen-activated protein kinase 14 | -0.56 | 2.9e-13 |
| 1499 | CTNNB1 | catenin (cadherin-associated protein), beta 1, 88kDa | -0.40 | 1.2e-03 |
| 1514 | CTSL | cathepsin L | -0.88 | 4.6e-11 |
| 1634 | DCN | decorin | -4.34 | 5.8e-47 |
| 1655 | DDX5 | DEAD (Asp-Glu-Ala-Asp) box helicase 5 | -0.21 | 1.4e-02 |
| 1839 | HBEGF | heparin-binding EGF-like growth factor | -1.27 | 6.4e-22 |
| 1956 | EGFR | epidermal growth factor receptor | -0.93 | 2.7e-06 |
| 1975 | EIF4B | eukaryotic translation initiation factor 4B | -1.06 | 1.3e-23 |
| 2002 | ELK1 | ELK1, member of ETS oncogene family | -0.07 | 4.7e-01 |
| 2017 | CTTN | cortactin | 0.23 | 9.3e-03 |
| 2064 | ERBB2 | erb-b2 receptor tyrosine kinase 2 | 0.85 | 3.8e-05 |
| 2065 | ERBB3 | erb-b2 receptor tyrosine kinase 3 | 2.14 | 2.7e-21 |
| 2066 | ERBB4 | erb-b2 receptor tyrosine kinase 4 | 1.88 | 9.6e-04 |
| 207 | AKT1 | v-akt murine thymoma viral oncogene homolog 1 | 0.54 | 4.8e-09 |
| 208 | AKT2 | v-akt murine thymoma viral oncogene homolog 2 | 0.08 | 5.2e-01 |
| 2099 | ESR1 | estrogen receptor 1 | -1.11 | 4.1e-03 |
| 2247 | FGF2 | fibroblast growth factor 2 (basic) | -4.15 | 3.0e-112 |
| 2260 | FGFR1 | fibroblast growth factor receptor 1 | -0.24 | 2.4e-01 |
| 22800 | RRAS2 | related RAS viral (r-ras) oncogene homolog 2 | -0.20 | 1.2e-01 |
| 22808 | MRAS | muscle RAS oncogene homolog | -1.21 | 5.7e-16 |
| 2316 | FLNA | filamin A, alpha | -2.34 | 2.2e-31 |
| 2317 | FLNB | filamin B, beta | -0.25 | 4.0e-02 |
| 2318 | FLNC | filamin C, gamma | -4.70 | 3.2e-85 |
| 2335 | FN1 | fibronectin 1 | -0.92 | 9.5e-04 |
| 23365 | ARHGEF12 | Rho guanine nucleotide exchange factor (GEF) 12 | -0.53 | 2.9e-06 |
| 23533 | PIK3R5 | phosphoinositide-3-kinase, regulatory subunit 5 | 0.24 | 2.6e-01 |
| 23624 | CBLC | Cbl proto-oncogene C, E3 ubiquitin protein ligase | 3.28 | 7.8e-13 |
| 2475 | MTOR | mechanistic target of rapamycin (serine/threonine kinase) | 0.74 | 9.0e-12 |
| 2535 | FZD2 | frizzled class receptor 2 | 0.64 | 2.8e-03 |
| 2549 | GAB1 | GRB2-associated binding protein 1 | -1.56 | 2.5e-39 |
| 2719 | GPC3 | glypican 3 | -1.53 | 7.1e-07 |
| 27250 | PDCD4 | programmed cell death 4 (neoplastic transformation inhibitor) | -1.13 | 9.5e-18 |
| 2817 | GPC1 | glypican 1 | 0.48 | 6.1e-03 |
| 286 | ANK1 | ankyrin 1, erythrocytic | 1.08 | 2.7e-03 |
| 287 | ANK2 | ankyrin 2, neuronal | -2.65 | 5.5e-35 |
| 288 | ANK3 | ankyrin 3, node of Ranvier (ankyrin G) | -0.23 | 2.0e-01 |
| 2885 | GRB2 | growth factor receptor-bound protein 2 | 0.00 | 9.9e-01 |
| 29102 | DROSHA | drosha, ribonuclease type III | 0.16 | 6.3e-02 |
| 3059 | HCLS1 | hematopoietic cell-specific Lyn substrate 1 | 0.67 | 8.4e-03 |
| 3082 | HGF | hepatocyte growth factor (hepapoietin A; scatter factor) | -2.52 | 1.1e-26 |
| 3091 | HIF1A | hypoxia inducible factor 1, alpha subunit (basic helix-loop-helix transcription factor) | 0.28 | 2.5e-01 |
| 3236 | HOXD10 | homeobox D10 | -1.34 | 9.1e-11 |
| 3265 | HRAS | Harvey rat sarcoma viral oncogene homolog | 0.90 | 2.8e-08 |
| 3316 | HSPB2 | heat shock 27kDa protein 2 | -3.50 | 2.9e-82 |
| 3339 | HSPG2 | heparan sulfate proteoglycan 2 | -0.88 | 3.7e-04 |
| 3479 | IGF1 | insulin-like growth factor 1 (somatomedin C) | -2.10 | 3.2e-15 |
| 3480 | IGF1R | insulin-like growth factor 1 receptor | -0.97 | 2.1e-05 |
| 3481 | IGF2 | insulin-like growth factor 2 | -2.47 | 1.2e-10 |
| 355 | FAS | Fas cell surface death receptor | -1.02 | 8.0e-12 |
| 356 | FASLG | Fas ligand (TNF superfamily, member 6) | -0.18 | 6.0e-01 |
| 3593 | IL12B | interleukin 12B | 0.36 | 1.9e-01 |
| 3673 | ITGA2 | integrin, alpha 2 (CD49B, alpha 2 subunit of VLA-2 receptor) | -0.51 | 4.4e-02 |
| 3678 | ITGA5 | integrin, alpha 5 (fibronectin receptor, alpha polypeptide) | -0.70 | 1.5e-06 |
| 3685 | ITGAV | integrin, alpha V | -0.98 | 3.2e-09 |
| 3688 | ITGB1 | integrin, beta 1 (fibronectin receptor, beta polypeptide, antigen CD29 includes MDF2, MSK12) | -0.74 | 1.8e-06 |
| 369 | ARAF | A-Raf proto-oncogene, serine/threonine kinase | -0.12 | 2.8e-01 |
| 3690 | ITGB3 | integrin, beta 3 (platelet glycoprotein IIIa, antigen CD61) | -1.76 | 2.6e-12 |
| 3693 | ITGB5 | integrin, beta 5 | -0.04 | 8.1e-01 |
| 3708 | ITPR1 | inositol 1,4,5-trisphosphate receptor, type 1 | -2.91 | 1.2e-76 |
| 3709 | ITPR2 | inositol 1,4,5-trisphosphate receptor, type 2 | -0.73 | 2.6e-05 |
| 3710 | ITPR3 | inositol 1,4,5-trisphosphate receptor, type 3 | 1.58 | 3.7e-18 |
| 3791 | KDR | kinase insert domain receptor | -2.20 | 7.7e-50 |
| 3845 | KRAS | Kirsten rat sarcoma viral oncogene homolog | 0.33 | 1.3e-02 |
| 387 | RHOA | ras homolog family member A | -0.24 | 2.2e-03 |
| 4060 | LUM | lumican | -1.78 | 2.1e-10 |
| 406902 | MIR10A | microRNA 10a | 0.13 | 4.9e-01 |
| 406903 | MIR10B | microRNA 10b | -2.06 | 2.2e-14 |
| 406991 | MIR21 | microRNA 21 | -2.07 | 2.0e-11 |
| 4087 | SMAD2 | SMAD family member 2 | -0.18 | 4.8e-02 |
| 4193 | MDM2 | MDM2 proto-oncogene, E3 ubiquitin protein ligase | 0.32 | 3.8e-02 |
| 4233 | MET | MET proto-oncogene, receptor tyrosine kinase | 0.68 | 2.2e-02 |
| 4313 | MMP2 | matrix metallopeptidase 2 | -1.07 | 2.0e-04 |
| 4318 | MMP9 | matrix metallopeptidase 9 | 3.28 | 7.7e-09 |
| 4478 | MSN | moesin | -0.70 | 2.2e-07 |
| 4609 | MYC | v-myc avian myelocytomatosis viral oncogene homolog | -1.35 | 1.7e-10 |
| 4659 | PPP1R12A | protein phosphatase 1, regulatory subunit 12A | -1.38 | 8.5e-37 |
| 4660 | PPP1R12B | protein phosphatase 1, regulatory subunit 12B | -4.10 | 4.3e-98 |
| 4893 | NRAS | neuroblastoma RAS viral (v-ras) oncogene homolog | 0.76 | 2.6e-07 |
| 5058 | PAK1 | p21 protein (Cdc42/Rac)-activated kinase 1 | 0.69 | 2.4e-11 |
| 51196 | PLCE1 | phospholipase C, epsilon 1 | -1.01 | 2.2e-06 |
| 51384 | WNT16 | wingless-type MMTV integration site family, member 16 | 0.22 | 6.2e-01 |
| 5170 | PDPK1 | 3-phosphoinositide dependent protein kinase 1 | -0.68 | 8.0e-12 |
| 5290 | PIK3CA | phosphatidylinositol-4,5-bisphosphate 3-kinase, catalytic subunit alpha | -0.34 | 1.2e-02 |
| 5291 | PIK3CB | phosphatidylinositol-4,5-bisphosphate 3-kinase, catalytic subunit beta | 0.19 | 8.7e-02 |
| 5293 | PIK3CD | phosphatidylinositol-4,5-bisphosphate 3-kinase, catalytic subunit delta | -0.71 | 1.6e-06 |
| 5294 | PIK3CG | phosphatidylinositol-4,5-bisphosphate 3-kinase, catalytic subunit gamma | -0.98 | 4.5e-05 |
| 5295 | PIK3R1 | phosphoinositide-3-kinase, regulatory subunit 1 (alpha) | -0.80 | 2.1e-03 |
| 5296 | PIK3R2 | phosphoinositide-3-kinase, regulatory subunit 2 (beta) | 0.67 | 6.5e-08 |
| 5328 | PLAU | plasminogen activator, urokinase | 0.81 | 7.8e-04 |
| 5329 | PLAUR | plasminogen activator, urokinase receptor | 1.21 | 1.2e-07 |
| 5335 | PLCG1 | phospholipase C, gamma 1 | -0.02 | 9.1e-01 |
| 5336 | PLCG2 | phospholipase C, gamma 2 (phosphatidylinositol-specific) | -0.70 | 1.7e-05 |
| 54361 | WNT4 | wingless-type MMTV integration site family, member 4 | -2.70 | 5.9e-33 |
| 54776 | PPP1R12C | protein phosphatase 1, regulatory subunit 12C | -1.22 | 6.9e-32 |
| 5499 | PPP1CA | protein phosphatase 1, catalytic subunit, alpha isozyme | 1.41 | 9.0e-36 |
| 5500 | PPP1CB | protein phosphatase 1, catalytic subunit, beta isozyme | -0.25 | 5.2e-02 |
| 5501 | PPP1CC | protein phosphatase 1, catalytic subunit, gamma isozyme | -0.17 | 1.0e-01 |
| 5566 | PRKACA | protein kinase, cAMP-dependent, catalytic, alpha | -0.17 | 1.0e-01 |
| 5567 | PRKACB | protein kinase, cAMP-dependent, catalytic, beta | -1.29 | 1.2e-22 |
| 5568 | PRKACG | protein kinase, cAMP-dependent, catalytic, gamma | 0.05 | 8.3e-01 |
| 5578 | PRKCA | protein kinase C, alpha | -1.99 | 8.5e-30 |
| 5579 | PRKCB | protein kinase C, beta | -1.76 | 5.6e-18 |
| 5582 | PRKCG | protein kinase C, gamma | 0.28 | 5.7e-01 |
| 5594 | MAPK1 | mitogen-activated protein kinase 1 | -0.24 | 2.6e-02 |
| 5595 | MAPK3 | mitogen-activated protein kinase 3 | -0.86 | 1.2e-13 |
| 5600 | MAPK11 | mitogen-activated protein kinase 11 | -1.30 | 3.5e-16 |
| 5603 | MAPK13 | mitogen-activated protein kinase 13 | 2.49 | 3.5e-23 |
| 5604 | MAP2K1 | mitogen-activated protein kinase kinase 1 | -0.05 | 6.3e-01 |
| 5605 | MAP2K2 | mitogen-activated protein kinase kinase 2 | 0.76 | 2.3e-06 |
| 5613 | PRKX | protein kinase, X-linked | 0.96 | 1.9e-08 |
| 5727 | PTCH1 | patched 1 | -1.17 | 9.4e-14 |
| 5747 | PTK2 | protein tyrosine kinase 2 | -0.01 | 9.3e-01 |
| 5777 | PTPN6 | protein tyrosine phosphatase, non-receptor type 6 | 1.62 | 1.3e-24 |
| 5781 | PTPN11 | protein tyrosine phosphatase, non-receptor type 11 | -0.53 | 1.8e-06 |
| 5829 | PXN | paxillin | -0.52 | 2.4e-08 |
| 5879 | RAC1 | ras-related C3 botulinum toxin substrate 1 (rho family, small GTP binding protein Rac1) | 0.02 | 7.8e-01 |
| 5894 | RAF1 | Raf-1 proto-oncogene, serine/threonine kinase | -0.11 | 1.2e-01 |
| 595 | CCND1 | cyclin D1 | 0.84 | 4.4e-04 |
| 5962 | RDX | radixin | -0.49 | 8.3e-05 |
| 60 | ACTB | actin, beta | -0.02 | 8.8e-01 |
| 60495 | HPSE2 | heparanase 2 (inactive) | -4.85 | 2.5e-50 |
| 6093 | ROCK1 | Rho-associated, coiled-coil containing protein kinase 1 | -0.82 | 1.6e-12 |
| 6194 | RPS6 | ribosomal protein S6 | -0.28 | 4.6e-02 |
| 6198 | RPS6KB1 | ribosomal protein S6 kinase, 70kDa, polypeptide 1 | -0.37 | 1.3e-04 |
| 6199 | RPS6KB2 | ribosomal protein S6 kinase, 70kDa, polypeptide 2 | 1.03 | 2.9e-17 |
| 6237 | RRAS | related RAS viral (r-ras) oncogene homolog | -1.46 | 2.0e-20 |
| 6300 | MAPK12 | mitogen-activated protein kinase 12 | -0.73 | 1.2e-03 |
| 6382 | SDC1 | syndecan 1 | 3.57 | 3.8e-31 |
| 6383 | SDC2 | syndecan 2 | -1.06 | 1.3e-06 |
| 6385 | SDC4 | syndecan 4 | 0.67 | 6.3e-05 |
| 6548 | SLC9A1 | solute carrier family 9, subfamily A (NHE1, cation proton antiporter 1), member 1 | 0.51 | 1.7e-05 |
| 6608 | SMO | smoothened, frizzled class receptor | -0.47 | 8.7e-03 |
| 6654 | SOS1 | son of sevenless homolog 1 (Drosophila) | -0.44 | 1.5e-06 |
| 6655 | SOS2 | son of sevenless homolog 2 (Drosophila) | -0.83 | 1.9e-19 |
| 6714 | SRC | SRC proto-oncogene, non-receptor tyrosine kinase | 0.08 | 5.0e-01 |
| 673 | BRAF | B-Raf proto-oncogene, serine/threonine kinase | 0.82 | 1.7e-09 |
| 6774 | STAT3 | signal transducer and activator of transcription 3 (acute-phase response factor) | -0.02 | 8.3e-01 |
| 7023 | TFAP4 | transcription factor AP-4 (activating enhancer binding protein 4) | -0.07 | 5.7e-01 |
| 7040 | TGFB1 | transforming growth factor, beta 1 | -0.65 | 7.3e-05 |
| 7042 | TGFB2 | transforming growth factor, beta 2 | -1.33 | 1.4e-08 |
| 7057 | THBS1 | thrombospondin 1 | -2.40 | 1.1e-22 |
| 7074 | TIAM1 | T-cell lymphoma invasion and metastasis 1 | -1.45 | 2.9e-18 |
| 7078 | TIMP3 | TIMP metallopeptidase inhibitor 3 | -3.52 | 2.5e-51 |
| 7097 | TLR2 | toll-like receptor 2 | 0.77 | 3.3e-05 |
| 7099 | TLR4 | toll-like receptor 4 | -1.37 | 1.2e-11 |
| 71 | ACTG1 | actin gamma 1 | 0.30 | 1.9e-02 |
| 7124 | TNF | tumor necrosis factor | 1.73 | 1.2e-05 |
| 7157 | TP53 | tumor protein p53 | 0.53 | 2.8e-03 |
| 7291 | TWIST1 | twist family bHLH transcription factor 1 | -1.27 | 1.4e-05 |
| 7410 | VAV2 | vav 2 guanine nucleotide exchange factor | 0.41 | 4.8e-04 |
| 7422 | VEGFA | vascular endothelial growth factor A | 0.57 | 2.4e-03 |
| 7430 | EZR | ezrin | 1.51 | 6.5e-23 |
| 7448 | VTN | vitronectin | -2.59 | 4.3e-19 |
| 7471 | WNT1 | wingless-type MMTV integration site family, member 1 | 0.17 | 6.0e-01 |
| 7472 | WNT2 | wingless-type MMTV integration site family member 2 | -1.34 | 9.0e-07 |
| 7473 | WNT3 | wingless-type MMTV integration site family, member 3 | -0.63 | 5.6e-02 |
| 7474 | WNT5A | wingless-type MMTV integration site family, member 5A | -0.69 | 2.8e-03 |
| 7475 | WNT6 | wingless-type MMTV integration site family, member 6 | -0.58 | 1.4e-01 |
| 7476 | WNT7A | wingless-type MMTV integration site family, member 7A | 2.85 | 4.4e-04 |
| 7477 | WNT7B | wingless-type MMTV integration site family, member 7B | 2.71 | 8.1e-09 |
| 7478 | WNT8A | wingless-type MMTV integration site family, member 8A | -0.23 | 4.1e-01 |
| 7479 | WNT8B | wingless-type MMTV integration site family, member 8B | 0.90 | 3.9e-04 |
| 7480 | WNT10B | wingless-type MMTV integration site family, member 10B | 0.98 | 1.0e-03 |
| 7481 | WNT11 | wingless-type MMTV integration site family, member 11 | 0.69 | 1.4e-01 |
| 7482 | WNT2B | wingless-type MMTV integration site family, member 2B | -2.35 | 4.4e-27 |
| 7483 | WNT9A | wingless-type MMTV integration site family, member 9A | -1.58 | 1.0e-08 |
| 7484 | WNT9B | wingless-type MMTV integration site family, member 9B | -1.70 | 5.1e-08 |
| 7855 | FZD5 | frizzled class receptor 5 | 1.83 | 5.8e-12 |
| 7976 | FZD3 | frizzled class receptor 3 | 0.58 | 6.3e-04 |
| 79923 | NANOG | Nanog homeobox | -0.03 | 9.2e-01 |
| 80326 | WNT10A | wingless-type MMTV integration site family, member 10A | 2.18 | 1.7e-05 |
| 81029 | WNT5B | wingless-type MMTV integration site family, member 5B | -1.32 | 1.8e-06 |
| 815 | CAMK2A | calcium/calmodulin-dependent protein kinase II alpha | -4.90 | 5.4e-81 |
| 81578 | COL21A1 | collagen, type XXI, alpha 1 | -2.31 | 7.8e-15 |
| 816 | CAMK2B | calcium/calmodulin-dependent protein kinase II beta | 1.05 | 1.4e-02 |
| 817 | CAMK2D | calcium/calmodulin-dependent protein kinase II delta | -0.96 | 7.9e-11 |
| 818 | CAMK2G | calcium/calmodulin-dependent protein kinase II gamma | -0.99 | 4.9e-27 |
| 8321 | FZD1 | frizzled class receptor 1 | -0.73 | 6.8e-06 |
| 8322 | FZD4 | frizzled class receptor 4 | -1.99 | 6.1e-54 |
| 8323 | FZD6 | frizzled class receptor 6 | 0.27 | 1.6e-01 |
| 8324 | FZD7 | frizzled class receptor 7 | -1.85 | 7.1e-15 |
| 8325 | FZD8 | frizzled class receptor 8 | 0.84 | 1.0e-02 |
| 8326 | FZD9 | frizzled class receptor 9 | 0.73 | 7.6e-03 |
| 836 | CASP3 | caspase 3, apoptosis-related cysteine peptidase | 0.88 | 6.8e-18 |
| 84309 | NUDT16L1 | nudix (nucleoside diphosphate linked moiety X)-type motif 16-like 1 | 0.68 | 2.1e-08 |
| 8503 | PIK3R3 | phosphoinositide-3-kinase, regulatory subunit 3 (gamma) | 1.28 | 1.7e-10 |
| 857 | CAV1 | caveolin 1, caveolae protein, 22kDa | -3.34 | 1.0e-80 |
| 858 | CAV2 | caveolin 2 | -2.30 | 5.6e-54 |
| 859 | CAV3 | caveolin 3 | -0.87 | 4.5e-04 |
| 867 | CBL | Cbl proto-oncogene, E3 ubiquitin protein ligase | -0.47 | 4.7e-05 |
| 868 | CBLB | Cbl proto-oncogene B, E3 ubiquitin protein ligase | 0.30 | 1.1e-02 |
| 8826 | IQGAP1 | IQ motif containing GTPase activating protein 1 | -0.89 | 5.5e-15 |
| 89780 | WNT3A | wingless-type MMTV integration site family, member 3A | 1.40 | 1.3e-04 |
| 9138 | ARHGEF1 | Rho guanine nucleotide exchange factor (GEF) 1 | 0.13 | 2.6e-01 |
| 9475 | ROCK2 | Rho-associated, coiled-coil containing protein kinase 2 | -1.35 | 3.4e-18 |
| 960 | CD44 | CD44 molecule (Indian blood group) | -1.03 | 5.3e-06 |
| 967 | CD63 | CD63 molecule | 0.16 | 2.1e-01 |
| 998 | CDC42 | cell division cycle 42 | -0.03 | 8.1e-01 |

| ENTREZID | SYMBOL | GENENAME | FC | ADJ.PVAL |
| --- | --- | --- | --- | --- |

(Page generated on Tue Aug 25 12:05:03 2015 by ReportingTools 2.9.1 and hwriter 1.3.2)
