# Supplementary material for: Bioconductor’s EnrichmentBrowser: seamless navigation through combined results of set- & network-based enrichment analysis
Source: BMC Bioinformatics. 2016 Jan 20;17:45. doi: 10.1186/s12859-016-0884-1 (PMC4721010; doi:10.1186/s12859-016-0884-1)
Supplement: Supplementary file 3 — EnrichmentBrowser output (TCGA RNA-seq data). Unzip and open the contained index.html in the browser to view the contents of this file (tested with Firefox 39.0). (ZIP 7116.8 kb) [file 12859_2016_884_MOESM3_ESM.zip › hsa05206.html]

hsa05206: Gene Report


## hsa05206: Gene Report

| ENTREZID | SYMBOL | GENENAME | FC | ADJ.PVAL |
| --- | --- | --- | --- | --- |
| ENTREZID | SYMBOL | GENENAME | FC | ADJ.PVAL |
| 10018 | BCL2L11 | BCL2-like 11 (apoptosis facilitator) | 0.97 | 2.1e-13 |
| 100500809 | MIR23C | microRNA 23c | 0.32 | 4.4e-02 |
| 100532731 | COMMD3-BMI1 | COMMD3-BMI1 readthrough | 0.33 | 6.9e-03 |
| 1021 | CDK6 | cyclin-dependent kinase 6 | -0.93 | 6.5e-06 |
| 10253 | SPRY2 | sprouty homolog 2 (Drosophila) | -0.68 | 1.9e-03 |
| 1026 | CDKN1A | cyclin-dependent kinase inhibitor 1A (p21, Cip1) | 0.08 | 7.6e-01 |
| 1027 | CDKN1B | cyclin-dependent kinase inhibitor 1B (p27, Kip1) | -1.42 | 1.6e-15 |
| 1029 | CDKN2A | cyclin-dependent kinase inhibitor 2A | 3.96 | 1.9e-09 |
| 10297 | APC2 | adenomatosis polyposis coli 2 | -0.95 | 7.8e-11 |
| 10298 | PAK4 | p21 protein (Cdc42/Rac)-activated kinase 4 | 0.75 | 7.7e-09 |
| 10642 | IGF2BP1 | insulin-like growth factor 2 mRNA binding protein 1 | 2.10 | 1.2e-05 |
| 11186 | RASSF1 | Ras association (RalGDS/AF-6) domain family member 1 | -0.17 | 8.4e-02 |
| 113130 | CDCA5 | cell division cycle associated 5 | 3.56 | 1.9e-27 |
| 131405 | TRIM71 | tripartite motif containing 71, E3 ubiquitin protein ligase | 0.40 | 2.7e-01 |
| 1387 | CREBBP | CREB binding protein | -0.60 | 2.6e-08 |
| 1398 | CRK | v-crk avian sarcoma virus CT10 oncogene homolog | -0.22 | 1.6e-02 |
| 1399 | CRKL | v-crk avian sarcoma virus CT10 oncogene homolog-like | -0.33 | 2.1e-04 |
| 1545 | CYP1B1 | cytochrome P450, family 1, subfamily B, polypeptide 1 | -3.74 | 2.5e-73 |
| 1591 | CYP24A1 | cytochrome P450, family 24, subfamily A, polypeptide 1 | 0.81 | 1.5e-01 |
| 1786 | DNMT1 | DNA (cytosine-5-)-methyltransferase 1 | 1.30 | 1.9e-20 |
| 1788 | DNMT3A | DNA (cytosine-5-)-methyltransferase 3 alpha | 1.20 | 1.5e-18 |
| 1789 | DNMT3B | DNA (cytosine-5-)-methyltransferase 3 beta | 2.47 | 3.2e-20 |
| 1869 | E2F1 | E2F transcription factor 1 | 2.99 | 8.8e-21 |
| 1870 | E2F2 | E2F transcription factor 2 | 4.05 | 2.8e-42 |
| 1871 | E2F3 | E2F transcription factor 3 | 1.47 | 5.0e-26 |
| 1944 | EFNA3 | ephrin-A3 | 1.98 | 1.8e-13 |
| 1956 | EGFR | epidermal growth factor receptor | -0.93 | 2.7e-06 |
| 2033 | EP300 | E1A binding protein p300 | -0.20 | 1.2e-01 |
| 2064 | ERBB2 | erb-b2 receptor tyrosine kinase 2 | 0.85 | 3.8e-05 |
| 2065 | ERBB3 | erb-b2 receptor tyrosine kinase 3 | 2.14 | 2.7e-21 |
| 2146 | EZH2 | enhancer of zeste 2 polycomb repressive complex 2 subunit | 2.80 | 1.7e-35 |
| 2261 | FGFR3 | fibroblast growth factor receptor 3 | 0.59 | 1.4e-01 |
| 23405 | DICER1 | dicer 1, ribonuclease type III | -0.26 | 3.0e-02 |
| 23411 | SIRT1 | sirtuin 1 | -0.96 | 8.5e-18 |
| 23414 | ZFPM2 | zinc finger protein, FOG family member 2 | -4.83 | 4.9e-115 |
| 2475 | MTOR | mechanistic target of rapamycin (serine/threonine kinase) | 0.74 | 9.0e-12 |
| 25 | ABL1 | ABL proto-oncogene 1, non-receptor tyrosine kinase | -1.00 | 8.4e-22 |
| 27086 | FOXP1 | forkhead box P1 | -1.29 | 1.6e-22 |
| 27165 | GLS2 | glutaminase 2 (liver, mitochondrial) | -0.25 | 1.6e-01 |
| 27250 | PDCD4 | programmed cell death 4 (neoplastic transformation inhibitor) | -1.13 | 9.5e-18 |
| 2744 | GLS | glutaminase | -0.84 | 9.2e-07 |
| 2885 | GRB2 | growth factor receptor-bound protein 2 | 0.00 | 9.9e-01 |
| 3065 | HDAC1 | histone deacetylase 1 | 0.41 | 8.5e-07 |
| 3162 | HMOX1 | heme oxygenase (decycling) 1 | 0.65 | 1.3e-03 |
| 3190 | HNRNPK | heterogeneous nuclear ribonucleoprotein K | 0.17 | 2.3e-02 |
| 3236 | HOXD10 | homeobox D10 | -1.34 | 9.1e-11 |
| 324 | APC | adenomatous polyposis coli | -0.50 | 2.4e-06 |
| 3265 | HRAS | Harvey rat sarcoma viral oncogene homolog | 0.90 | 2.8e-08 |
| 3371 | TNC | tenascin C | 0.25 | 4.5e-01 |
| 3551 | IKBKB | inhibitor of kappa light polypeptide gene enhancer in B-cells, kinase beta | -0.27 | 2.9e-03 |
| 3667 | IRS1 | insulin receptor substrate 1 | -2.16 | 2.8e-24 |
| 3678 | ITGA5 | integrin, alpha 5 (fibronectin receptor, alpha polypeptide) | -0.70 | 1.5e-06 |
| 3690 | ITGB3 | integrin, beta 3 (platelet glycoprotein IIIa, antigen CD61) | -1.76 | 2.6e-12 |
| 3845 | KRAS | Kirsten rat sarcoma viral oncogene homolog | 0.33 | 1.3e-02 |
| 387 | RHOA | ras homolog family member A | -0.24 | 2.2e-03 |
| 3925 | STMN1 | stathmin 1 | 1.34 | 1.1e-13 |
| 399694 | SHC4 | SHC (Src homology 2 domain containing) family, member 4 | -1.02 | 1.9e-05 |
| 406881 | MIRLET7A1 | microRNA let-7a-1 | -0.10 | 6.1e-01 |
| 406885 | MIRLET7C | microRNA let-7c | -0.32 | 3.7e-02 |
| 406886 | MIRLET7D | microRNA let-7d | -2.69 | 4.0e-17 |
| 406888 | MIRLET7F1 | microRNA let-7f-1 | -0.53 | 7.1e-03 |
| 406890 | MIRLET7G | microRNA let-7g | 0.29 | 8.9e-02 |
| 406891 | MIRLET7I | microRNA let-7i | -0.50 | 2.0e-02 |
| 406893 | MIR101-1 | microRNA 101-1 | -0.04 | 8.0e-01 |
| 406894 | MIR101-2 | microRNA 101-2 | 0.65 | 2.5e-03 |
| 406896 | MIR103A2 | microRNA 103a-2 | -0.18 | 3.3e-01 |
| 406900 | MIR106B | microRNA 106b | -0.31 | 1.8e-01 |
| 406902 | MIR10A | microRNA 10a | 0.13 | 4.9e-01 |
| 406903 | MIR10B | microRNA 10b | -2.06 | 2.2e-14 |
| 406905 | MIR1-2 | microRNA 1-2 | 0.07 | 7.1e-01 |
| 406911 | MIR125B1 | microRNA 125b-1 | 0.05 | 7.8e-01 |
| 406912 | MIR125B2 | microRNA 125b-2 | -0.52 | 8.2e-04 |
| 406913 | MIR126 | microRNA 126 | -2.14 | 4.3e-16 |
| 406922 | MIR133A1 | microRNA 133a-1 | -0.96 | 9.5e-07 |
| 406926 | MIR135A2 | microRNA 135a-2 | -0.38 | 2.6e-02 |
| 406933 | MIR141 | microRNA 141 | 1.54 | 6.4e-07 |
| 406938 | MIR146A | microRNA 146a | 0.28 | 1.5e-01 |
| 406948 | MIR15A | microRNA 15a | 0.64 | 3.4e-04 |
| 406954 | MIR181A2 | microRNA 181a-2 | 0.55 | 1.5e-03 |
| 406955 | MIR181B1 | microRNA 181b-1 | 0.42 | 2.4e-03 |
| 406956 | MIR181B2 | microRNA 181b-2 | 0.51 | 4.4e-03 |
| 406957 | MIR181C | microRNA 181c | 0.21 | 1.9e-01 |
| 406967 | MIR192 | microRNA 192 | 0.59 | 2.2e-03 |
| 406969 | MIR194-1 | microRNA 194-1 | 0.45 | 1.5e-02 |
| 406971 | MIR195 | microRNA 195 | 0.49 | 1.6e-03 |
| 406976 | MIR199A1 | microRNA 199a-1 | -0.11 | 5.7e-01 |
| 406983 | MIR200A | microRNA 200a | 0.59 | 3.4e-02 |
| 406984 | MIR200B | microRNA 200b | 0.34 | 2.1e-01 |
| 406985 | MIR200C | microRNA 200c | 0.60 | 3.1e-04 |
| 406988 | MIR205 | microRNA 205 | 0.90 | 3.2e-05 |
| 406991 | MIR21 | microRNA 21 | -2.07 | 2.0e-11 |
| 406992 | MIR210 | microRNA 210 | 1.38 | 5.0e-08 |
| 406997 | MIR215 | microRNA 215 | 0.51 | 1.5e-02 |
| 407006 | MIR221 | microRNA 221 | -2.16 | 1.5e-11 |
| 407007 | MIR222 | microRNA 222 | -0.09 | 6.2e-01 |
| 407008 | MIR223 | microRNA 223 | -0.23 | 4.0e-01 |
| 407010 | MIR23A | microRNA 23a | -0.85 | 2.5e-03 |
| 407011 | MIR23B | microRNA 23b | -2.03 | 5.4e-18 |
| 407014 | MIR25 | microRNA 25 | 0.57 | 1.8e-02 |
| 407016 | MIR26A2 | microRNA 26a-2 | -0.58 | 3.4e-03 |
| 407017 | MIR26B | microRNA 26b | 0.49 | 1.2e-02 |
| 407018 | MIR27A | microRNA 27a | -0.51 | 8.8e-02 |
| 407019 | MIR27B | microRNA 27b | -2.66 | 1.1e-21 |
| 407021 | MIR29A | microRNA 29a | 0.34 | 2.6e-02 |
| 407024 | MIR29B1 | microRNA 29b-1 | 0.33 | 2.2e-02 |
| 407025 | MIR29B2 | microRNA 29b-2 | -1.88 | 4.2e-11 |
| 407026 | MIR29C | microRNA 29c | -2.09 | 2.0e-11 |
| 407029 | MIR30A | microRNA 30a | 0.48 | 2.6e-03 |
| 407032 | MIR30C2 | microRNA 30c-2 | -0.56 | 2.3e-02 |
| 407040 | MIR34A | microRNA 34a | -0.76 | 1.4e-03 |
| 407041 | MIR34B | microRNA 34b | 0.23 | 2.2e-01 |
| 407042 | MIR34C | microRNA 34c | -0.66 | 5.6e-02 |
| 407043 | MIR7-1 | microRNA 7-1 | -0.44 | 6.6e-02 |
| 407045 | MIR7-3 | microRNA 7-3 | 0.43 | 6.1e-03 |
| 4082 | MARCKS | myristoylated alanine-rich protein kinase C substrate | -0.36 | 3.5e-02 |
| 4170 | MCL1 | myeloid cell leukemia 1 | -0.12 | 2.4e-01 |
| 4193 | MDM2 | MDM2 proto-oncogene, E3 ubiquitin protein ligase | 0.32 | 3.8e-02 |
| 4194 | MDM4 | MDM4, p53 regulator | -0.10 | 4.3e-01 |
| 4233 | MET | MET proto-oncogene, receptor tyrosine kinase | 0.68 | 2.2e-02 |
| 4318 | MMP9 | matrix metallopeptidase 9 | 3.28 | 7.7e-09 |
| 4325 | MMP16 | matrix metallopeptidase 16 (membrane-inserted) | -1.63 | 1.1e-09 |
| 4363 | ABCC1 | ATP-binding cassette, sub-family C (CFTR/MRP), member 1 | 1.19 | 3.4e-26 |
| 442891 | MIR135B | microRNA 135b | 0.34 | 4.4e-02 |
| 442898 | MIR324 | microRNA 324 | 0.34 | 1.7e-01 |
| 442902 | MIR330 | microRNA 330 | 0.55 | 1.1e-03 |
| 442903 | MIR331 | microRNA 331 | -0.94 | 1.3e-06 |
| 442904 | MIR335 | microRNA 335 | 0.32 | 2.4e-02 |
| 442910 | MIR345 | microRNA 345 | 0.21 | 1.9e-01 |
| 4609 | MYC | v-myc avian myelocytomatosis viral oncogene homolog | -1.35 | 1.7e-10 |
| 472 | ATM | ATM serine/threonine kinase | -0.93 | 2.3e-14 |
| 4790 | NFKB1 | nuclear factor of kappa light polypeptide gene enhancer in B-cells 1 | -0.70 | 5.4e-11 |
| 4851 | NOTCH1 | notch 1 | 0.03 | 8.7e-01 |
| 4853 | NOTCH2 | notch 2 | -0.73 | 3.3e-08 |
| 4854 | NOTCH3 | notch 3 | 0.65 | 1.8e-04 |
| 4855 | NOTCH4 | notch 4 | -1.11 | 9.8e-25 |
| 4893 | NRAS | neuroblastoma RAS viral (v-ras) oncogene homolog | 0.76 | 2.6e-07 |
| 494335 | MIR423 | microRNA 423 | -0.36 | 7.4e-02 |
| 5154 | PDGFA | platelet-derived growth factor alpha polypeptide | -0.37 | 9.1e-02 |
| 5155 | PDGFB | platelet-derived growth factor beta polypeptide | -0.41 | 7.9e-03 |
| 5156 | PDGFRA | platelet-derived growth factor receptor, alpha polypeptide | -3.00 | 7.1e-35 |
| 5159 | PDGFRB | platelet-derived growth factor receptor, beta polypeptide | -1.85 | 6.8e-23 |
| 5243 | ABCB1 | ATP-binding cassette, sub-family B (MDR/TAP), member 1 | -2.75 | 1.3e-53 |
| 5268 | SERPINB5 | serpin peptidase inhibitor, clade B (ovalbumin), member 5 | 2.74 | 7.2e-08 |
| 5290 | PIK3CA | phosphatidylinositol-4,5-bisphosphate 3-kinase, catalytic subunit alpha | -0.34 | 1.2e-02 |
| 5292 | PIM1 | Pim-1 proto-oncogene, serine/threonine kinase | -1.04 | 1.2e-11 |
| 5296 | PIK3R2 | phosphoinositide-3-kinase, regulatory subunit 2 (beta) | 0.67 | 6.5e-08 |
| 5328 | PLAU | plasminogen activator, urokinase | 0.81 | 7.8e-04 |
| 5335 | PLCG1 | phospholipase C, gamma 1 | -0.02 | 9.1e-01 |
| 5336 | PLCG2 | phospholipase C, gamma 2 (phosphatidylinositol-specific) | -0.70 | 1.7e-05 |
| 54541 | DDIT4 | DNA-damage-inducible transcript 4 | 1.10 | 8.0e-06 |
| 5578 | PRKCA | protein kinase C, alpha | -1.99 | 8.5e-30 |
| 5579 | PRKCB | protein kinase C, beta | -1.76 | 5.6e-18 |
| 5581 | PRKCE | protein kinase C, epsilon | -0.81 | 7.9e-11 |
| 5582 | PRKCG | protein kinase C, gamma | 0.28 | 5.7e-01 |
| 5594 | MAPK1 | mitogen-activated protein kinase 1 | -0.24 | 2.6e-02 |
| 5598 | MAPK7 | mitogen-activated protein kinase 7 | -0.23 | 3.1e-02 |
| 5604 | MAP2K1 | mitogen-activated protein kinase kinase 1 | -0.05 | 6.3e-01 |
| 5605 | MAP2K2 | mitogen-activated protein kinase kinase 2 | 0.76 | 2.3e-06 |
| 5728 | PTEN | phosphatase and tensin homolog | -0.86 | 7.4e-09 |
| 574031 | MIR363 | microRNA 363 | 0.37 | 3.6e-02 |
| 5743 | PTGS2 | prostaglandin-endoperoxide synthase 2 (prostaglandin G/H synthase and cyclooxygenase) | -1.00 | 7.9e-03 |
| 574457 | MIR181D | microRNA 181d | 0.07 | 6.8e-01 |
| 57521 | RPTOR | regulatory associated protein of MTOR, complex 1 | -0.24 | 9.8e-03 |
| 578 | BAK1 | BCL2-antagonist/killer 1 | 1.23 | 5.4e-16 |
| 5894 | RAF1 | Raf-1 proto-oncogene, serine/threonine kinase | -0.11 | 1.2e-01 |
| 595 | CCND1 | cyclin D1 | 0.84 | 4.4e-04 |
| 596 | BCL2 | B-cell CLL/lymphoma 2 | -2.24 | 1.7e-33 |
| 5962 | RDX | radixin | -0.49 | 8.3e-05 |
| 599 | BCL2L2 | BCL2-like 2 | -1.24 | 1.3e-36 |
| 6093 | ROCK1 | Rho-associated, coiled-coil containing protein kinase 1 | -0.82 | 1.6e-12 |
| 619552 | MIR483 | microRNA 483 | 0.48 | 1.3e-02 |
| 63923 | TNN | tenascin N | -0.20 | 5.6e-01 |
| 6464 | SHC1 | SHC (Src homology 2 domain containing) transforming protein 1 | -0.10 | 3.4e-01 |
| 648 | BMI1 | BMI1 proto-oncogene, polycomb ring finger | -0.81 | 8.0e-04 |
| 6541 | SLC7A1 | solute carrier family 7 (cationic amino acid transporter, y+ system), member 1 | 1.06 | 1.6e-09 |
| 659 | BMPR2 | bone morphogenetic protein receptor, type II (serine/threonine kinase) | -0.92 | 1.9e-14 |
| 6624 | FSCN1 | fascin actin-bundling protein 1 | 0.16 | 5.0e-01 |
| 6654 | SOS1 | son of sevenless homolog 1 (Drosophila) | -0.44 | 1.5e-06 |
| 6655 | SOS2 | son of sevenless homolog 2 (Drosophila) | -0.83 | 1.9e-19 |
| 6659 | SOX4 | SRY (sex determining region Y)-box 4 | 1.09 | 2.7e-11 |
| 672 | BRCA1 | breast cancer 1, early onset | 1.32 | 4.5e-12 |
| 6768 | ST14 | suppression of tumorigenicity 14 (colon carcinoma) | 3.40 | 1.5e-49 |
| 6774 | STAT3 | signal transducer and activator of transcription 3 (acute-phase response factor) | -0.02 | 8.3e-01 |
| 693187 | MIR602 | microRNA 602 | 0.07 | 7.1e-01 |
| 693200 | MIR615 | microRNA 615 | -0.01 | 9.6e-01 |
| 6935 | ZEB1 | zinc finger E-box binding homeobox 1 | -3.08 | 5.4e-73 |
| 7042 | TGFB2 | transforming growth factor, beta 2 | -1.33 | 1.4e-08 |
| 7057 | THBS1 | thrombospondin 1 | -2.40 | 1.1e-22 |
| 7078 | TIMP3 | TIMP metallopeptidase inhibitor 3 | -3.52 | 2.5e-51 |
| 7143 | TNR | tenascin R | 0.62 | 2.2e-02 |
| 7148 | TNXB | tenascin XB | -5.49 | 1.8e-117 |
| 7157 | TP53 | tumor protein p53 | 0.53 | 2.8e-03 |
| 7168 | TPM1 | tropomyosin 1 (alpha) | -2.37 | 6.7e-52 |
| 7329 | UBE2I | ubiquitin-conjugating enzyme E2I | -0.25 | 2.8e-03 |
| 7422 | VEGFA | vascular endothelial growth factor A | 0.57 | 2.4e-03 |
| 7430 | EZR | ezrin | 1.51 | 6.5e-23 |
| 7431 | VIM | vimentin | -0.96 | 2.1e-04 |
| 7473 | WNT3 | wingless-type MMTV integration site family, member 3 | -0.63 | 5.6e-02 |
| 7976 | FZD3 | frizzled class receptor 3 | 0.58 | 6.3e-04 |
| 8091 | HMGA2 | high mobility group AT-hook 2 | 2.52 | 7.5e-05 |
| 836 | CASP3 | caspase 3, apoptosis-related cysteine peptidase | 0.88 | 6.8e-18 |
| 8434 | RECK | reversion-inducing-cysteine-rich protein with kazal motifs | -2.75 | 1.6e-91 |
| 85414 | SLC45A3 | solute carrier family 45, member 3 | 0.12 | 5.1e-01 |
| 8626 | TP63 | tumor protein p63 | 0.80 | 2.6e-02 |
| 8651 | SOCS1 | suppressor of cytokine signaling 1 | 0.14 | 5.3e-01 |
| 8660 | IRS2 | insulin receptor substrate 2 | -2.11 | 3.7e-22 |
| 894 | CCND2 | cyclin D2 | -2.90 | 1.3e-37 |
| 89780 | WNT3A | wingless-type MMTV integration site family, member 3A | 1.40 | 1.3e-04 |
| 898 | CCNE1 | cyclin E1 | 3.74 | 4.9e-19 |
| 900 | CCNG1 | cyclin G1 | -0.67 | 7.8e-07 |
| 90427 | BMF | Bcl2 modifying factor | 0.36 | 5.4e-02 |
| 9134 | CCNE2 | cyclin E2 | 2.01 | 6.1e-12 |
| 9252 | RPS6KA5 | ribosomal protein S6 kinase, 90kDa, polypeptide 5 | -1.01 | 6.3e-08 |
| 9493 | KIF23 | kinesin family member 23 | 2.49 | 2.2e-23 |
| 960 | CD44 | CD44 molecule (Indian blood group) | -1.03 | 5.3e-06 |
| 9759 | HDAC4 | histone deacetylase 4 | -0.98 | 1.2e-15 |
| 9839 | ZEB2 | zinc finger E-box binding homeobox 2 | -2.55 | 4.3e-67 |
| 993 | CDC25A | cell division cycle 25A | 3.00 | 9.4e-31 |
| 994 | CDC25B | cell division cycle 25B | 0.83 | 8.7e-06 |
| 995 | CDC25C | cell division cycle 25C | 4.76 | 4.6e-62 |

| ENTREZID | SYMBOL | GENENAME | FC | ADJ.PVAL |
| --- | --- | --- | --- | --- |

(Page generated on Mon Aug 24 22:00:29 2015 by ReportingTools 2.9.1 and hwriter 1.3.2)
