# Supplementary material for: Bioconductor’s EnrichmentBrowser: seamless navigation through combined results of set- & network-based enrichment analysis
Source: BMC Bioinformatics. 2016 Jan 20;17:45. doi: 10.1186/s12859-016-0884-1 (PMC4721010; doi:10.1186/s12859-016-0884-1)
Supplement: Supplementary file 4 — GEO2KEGG target pathways. Unzip and open the contained index.html in the browser to view the contents of this file (tested with Firefox 39.0). ZIP 4597.76 kb [file 12859_2016_884_MOESM4_ESM.zip › GSE19728/hsa05214_kview.html]

hsa05214 
