# Supplementary material for: Bioconductor’s EnrichmentBrowser: seamless navigation through combined results of set- & network-based enrichment analysis
Source: BMC Bioinformatics. 2016 Jan 20;17:45. doi: 10.1186/s12859-016-0884-1 (PMC4721010; doi:10.1186/s12859-016-0884-1)
Supplement: Supplementary file 3 — EnrichmentBrowser output (TCGA RNA-seq data). Unzip and open the contained index.html in the browser to view the contents of this file (tested with Firefox 39.0). (ZIP 7116.8 kb) [file 12859_2016_884_MOESM3_ESM.zip › hsa05220.html]

hsa05220: Gene Report


## hsa05220: Gene Report

| ENTREZID | SYMBOL | GENENAME | FC | ADJ.PVAL |
| --- | --- | --- | --- | --- |
| ENTREZID | SYMBOL | GENENAME | FC | ADJ.PVAL |
| 10000 | AKT3 | v-akt murine thymoma viral oncogene homolog 3 | -3.49 | 6.4e-108 |
| 1019 | CDK4 | cyclin-dependent kinase 4 | 0.28 | 8.6e-03 |
| 1021 | CDK6 | cyclin-dependent kinase 6 | -0.93 | 6.5e-06 |
| 1026 | CDKN1A | cyclin-dependent kinase inhibitor 1A (p21, Cip1) | 0.08 | 7.6e-01 |
| 1027 | CDKN1B | cyclin-dependent kinase inhibitor 1B (p27, Kip1) | -1.42 | 1.6e-15 |
| 1029 | CDKN2A | cyclin-dependent kinase inhibitor 2A | 3.96 | 1.9e-09 |
| 1147 | CHUK | conserved helix-loop-helix ubiquitous kinase | 0.13 | 1.9e-01 |
| 1398 | CRK | v-crk avian sarcoma virus CT10 oncogene homolog | -0.22 | 1.6e-02 |
| 1399 | CRKL | v-crk avian sarcoma virus CT10 oncogene homolog-like | -0.33 | 2.1e-04 |
| 1487 | CTBP1 | C-terminal binding protein 1 | 0.30 | 2.3e-03 |
| 1488 | CTBP2 | C-terminal binding protein 2 | 0.28 | 1.6e-02 |
| 1869 | E2F1 | E2F transcription factor 1 | 2.99 | 8.8e-21 |
| 1870 | E2F2 | E2F transcription factor 2 | 4.05 | 2.8e-42 |
| 1871 | E2F3 | E2F transcription factor 3 | 1.47 | 5.0e-26 |
| 207 | AKT1 | v-akt murine thymoma viral oncogene homolog 1 | 0.54 | 4.8e-09 |
| 208 | AKT2 | v-akt murine thymoma viral oncogene homolog 2 | 0.08 | 5.2e-01 |
| 2122 | MECOM | MDS1 and EVI1 complex locus | 1.46 | 6.4e-12 |
| 23533 | PIK3R5 | phosphoinositide-3-kinase, regulatory subunit 5 | 0.24 | 2.6e-01 |
| 23624 | CBLC | Cbl proto-oncogene C, E3 ubiquitin protein ligase | 3.28 | 7.8e-13 |
| 25 | ABL1 | ABL proto-oncogene 1, non-receptor tyrosine kinase | -1.00 | 8.4e-22 |
| 25759 | SHC2 | SHC (Src homology 2 domain containing) transforming protein 2 | -2.29 | 2.9e-26 |
| 2885 | GRB2 | growth factor receptor-bound protein 2 | 0.00 | 9.9e-01 |
| 3065 | HDAC1 | histone deacetylase 1 | 0.41 | 8.5e-07 |
| 3066 | HDAC2 | histone deacetylase 2 | 0.46 | 4.6e-05 |
| 3265 | HRAS | Harvey rat sarcoma viral oncogene homolog | 0.90 | 2.8e-08 |
| 3551 | IKBKB | inhibitor of kappa light polypeptide gene enhancer in B-cells, kinase beta | -0.27 | 2.9e-03 |
| 369 | ARAF | A-Raf proto-oncogene, serine/threonine kinase | -0.12 | 2.8e-01 |
| 3845 | KRAS | Kirsten rat sarcoma viral oncogene homolog | 0.33 | 1.3e-02 |
| 399694 | SHC4 | SHC (Src homology 2 domain containing) family, member 4 | -1.02 | 1.9e-05 |
| 4088 | SMAD3 | SMAD family member 3 | -1.19 | 3.7e-21 |
| 4089 | SMAD4 | SMAD family member 4 | -0.86 | 4.9e-19 |
| 4193 | MDM2 | MDM2 proto-oncogene, E3 ubiquitin protein ligase | 0.32 | 3.8e-02 |
| 4609 | MYC | v-myc avian myelocytomatosis viral oncogene homolog | -1.35 | 1.7e-10 |
| 4790 | NFKB1 | nuclear factor of kappa light polypeptide gene enhancer in B-cells 1 | -0.70 | 5.4e-11 |
| 4792 | NFKBIA | nuclear factor of kappa light polypeptide gene enhancer in B-cells inhibitor, alpha | -0.36 | 7.9e-03 |
| 4893 | NRAS | neuroblastoma RAS viral (v-ras) oncogene homolog | 0.76 | 2.6e-07 |
| 5290 | PIK3CA | phosphatidylinositol-4,5-bisphosphate 3-kinase, catalytic subunit alpha | -0.34 | 1.2e-02 |
| 5291 | PIK3CB | phosphatidylinositol-4,5-bisphosphate 3-kinase, catalytic subunit beta | 0.19 | 8.7e-02 |
| 5293 | PIK3CD | phosphatidylinositol-4,5-bisphosphate 3-kinase, catalytic subunit delta | -0.71 | 1.6e-06 |
| 5294 | PIK3CG | phosphatidylinositol-4,5-bisphosphate 3-kinase, catalytic subunit gamma | -0.98 | 4.5e-05 |
| 5295 | PIK3R1 | phosphoinositide-3-kinase, regulatory subunit 1 (alpha) | -0.80 | 2.1e-03 |
| 5296 | PIK3R2 | phosphoinositide-3-kinase, regulatory subunit 2 (beta) | 0.67 | 6.5e-08 |
| 53358 | SHC3 | SHC (Src homology 2 domain containing) transforming protein 3 | -1.66 | 1.2e-09 |
| 5594 | MAPK1 | mitogen-activated protein kinase 1 | -0.24 | 2.6e-02 |
| 5595 | MAPK3 | mitogen-activated protein kinase 3 | -0.86 | 1.2e-13 |
| 5604 | MAP2K1 | mitogen-activated protein kinase kinase 1 | -0.05 | 6.3e-01 |
| 5605 | MAP2K2 | mitogen-activated protein kinase kinase 2 | 0.76 | 2.3e-06 |
| 572 | BAD | BCL2-associated agonist of cell death | 0.17 | 2.1e-01 |
| 5781 | PTPN11 | protein tyrosine phosphatase, non-receptor type 11 | -0.53 | 1.8e-06 |
| 5894 | RAF1 | Raf-1 proto-oncogene, serine/threonine kinase | -0.11 | 1.2e-01 |
| 5925 | RB1 | retinoblastoma 1 | -0.09 | 6.1e-01 |
| 595 | CCND1 | cyclin D1 | 0.84 | 4.4e-04 |
| 5970 | RELA | v-rel avian reticuloendotheliosis viral oncogene homolog A | 0.00 | 9.6e-01 |
| 598 | BCL2L1 | BCL2-like 1 | 0.66 | 6.2e-09 |
| 613 | BCR | breakpoint cluster region | 0.76 | 1.3e-11 |
| 6464 | SHC1 | SHC (Src homology 2 domain containing) transforming protein 1 | -0.10 | 3.4e-01 |
| 6654 | SOS1 | son of sevenless homolog 1 (Drosophila) | -0.44 | 1.5e-06 |
| 6655 | SOS2 | son of sevenless homolog 2 (Drosophila) | -0.83 | 1.9e-19 |
| 673 | BRAF | B-Raf proto-oncogene, serine/threonine kinase | 0.82 | 1.7e-09 |
| 6776 | STAT5A | signal transducer and activator of transcription 5A | -1.03 | 2.9e-16 |
| 6777 | STAT5B | signal transducer and activator of transcription 5B | -1.83 | 7.5e-74 |
| 7040 | TGFB1 | transforming growth factor, beta 1 | -0.65 | 7.3e-05 |
| 7042 | TGFB2 | transforming growth factor, beta 2 | -1.33 | 1.4e-08 |
| 7043 | TGFB3 | transforming growth factor, beta 3 | -1.52 | 1.4e-25 |
| 7046 | TGFBR1 | transforming growth factor, beta receptor 1 | -0.62 | 1.0e-09 |
| 7048 | TGFBR2 | transforming growth factor, beta receptor II (70/80kDa) | -1.75 | 6.2e-30 |
| 7157 | TP53 | tumor protein p53 | 0.53 | 2.8e-03 |
| 8503 | PIK3R3 | phosphoinositide-3-kinase, regulatory subunit 3 (gamma) | 1.28 | 1.7e-10 |
| 8517 | IKBKG | inhibitor of kappa light polypeptide gene enhancer in B-cells, kinase gamma | -0.37 | 1.1e-04 |
| 861 | RUNX1 | runt-related transcription factor 1 | 1.56 | 2.5e-12 |
| 867 | CBL | Cbl proto-oncogene, E3 ubiquitin protein ligase | -0.47 | 4.7e-05 |
| 868 | CBLB | Cbl proto-oncogene B, E3 ubiquitin protein ligase | 0.30 | 1.1e-02 |
| 9846 | GAB2 | GRB2-associated binding protein 2 | -1.77 | 3.4e-35 |

| ENTREZID | SYMBOL | GENENAME | FC | ADJ.PVAL |
| --- | --- | --- | --- | --- |

(Page generated on Tue Aug 25 12:04:33 2015 by ReportingTools 2.9.1 and hwriter 1.3.2)
