# Supplementary material for: Bioconductor’s EnrichmentBrowser: seamless navigation through combined results of set- & network-based enrichment analysis
Source: BMC Bioinformatics. 2016 Jan 20;17:45. doi: 10.1186/s12859-016-0884-1 (PMC4721010; doi:10.1186/s12859-016-0884-1)
Supplement: Supplementary file 3 — EnrichmentBrowser output (TCGA RNA-seq data). Unzip and open the contained index.html in the browser to view the contents of this file (tested with Firefox 39.0). (ZIP 7116.8 kb) [file 12859_2016_884_MOESM3_ESM.zip › index.html]

EnrichmentBrowser: Index of Result Files


## EnrichmentBrowser: Index of Result Files

DE Measures for each Gene

#### Combined Results

Top Table

Full Ranking

#### GSEA Results

Top Table

Full Ranking

#### NEA Results

Top Table

Full Ranking

#### PATHNET Results

Top Table

Full Ranking

(Page generated on Mon Aug 24 17:11:37 2015 by ReportingTools 2.9.1 and hwriter 1.3.2)
