# Supplementary material for: Bioconductor’s EnrichmentBrowser: seamless navigation through combined results of set- & network-based enrichment analysis
Source: BMC Bioinformatics. 2016 Jan 20;17:45. doi: 10.1186/s12859-016-0884-1 (PMC4721010; doi:10.1186/s12859-016-0884-1)
Supplement: Supplementary file 3 — EnrichmentBrowser output (TCGA RNA-seq data). Unzip and open the contained index.html in the browser to view the contents of this file (tested with Firefox 39.0). (ZIP 7116.8 kb) [file 12859_2016_884_MOESM3_ESM.zip › pathnet.html]

PATHNET - Table of Results


## PATHNET - Table of Results

| GENE.SET | TITLE | NR.GENES | NR.SIG.GENES | NR.SIG.COMB.GENES | P.VALUE | SET.VIEW | PATH.VIEW | GRAPH.VIEW |
| --- | --- | --- | --- | --- | --- | --- | --- | --- |
| GENE.SET | TITLE | NR.GENES | NR.SIG.GENES | NR.SIG.COMB.GENES | P.VALUE | SET.VIEW | PATH.VIEW | GRAPH.VIEW |
| hsa05205 | Proteoglycans in cancer | 204 | 156 | 161 | 3.92e-05 |  |  |  |
| hsa04915 | Estrogen signaling pathway | 100 | 64 | 79 | 1.11e-04 |  |  |  |
| hsa05220 | Chronic myeloid leukemia | 73 | 57 | 62 | 1.76e-04 |  |  |  |

| GENE.SET | TITLE | NR.GENES | NR.SIG.GENES | NR.SIG.COMB.GENES | P.VALUE | SET.VIEW | PATH.VIEW | GRAPH.VIEW |
| --- | --- | --- | --- | --- | --- | --- | --- | --- |

(Page generated on Tue Aug 25 12:04:34 2015 by ReportingTools 2.9.1 and hwriter 1.3.2)
