# Supplementary material for: Bioconductor’s EnrichmentBrowser: seamless navigation through combined results of set- & network-based enrichment analysis
Source: BMC Bioinformatics. 2016 Jan 20;17:45. doi: 10.1186/s12859-016-0884-1 (PMC4721010; doi:10.1186/s12859-016-0884-1)
Supplement: Supplementary file 4 — GEO2KEGG target pathways. Unzip and open the contained index.html in the browser to view the contents of this file (tested with Firefox 39.0). ZIP 4597.76 kb [file 12859_2016_884_MOESM4_ESM.zip › index.html]

KEGGdzPathwaysGEO: Target pathways


## KEGGdzPathwaysGEO: Target pathways

| GEO.ID | KEGG.ID | SET.VIEW | PATH.VIEW |
| --- | --- | --- | --- |
| GEO.ID | KEGG.ID | SET.VIEW | PATH.VIEW |
| GSE1297 | hsa05010 |  |  |
| GSE14762 | hsa05211 |  |  |
| GSE15471 | hsa05212 |  |  |
| GSE16515 | hsa05212 |  |  |
| GSE18842 | hsa05223 |  |  |
| GSE19188 | hsa05223 |  |  |
| GSE19728 | hsa05214 |  |  |
| GSE20153 | hsa05012 |  |  |
| GSE20291 | hsa05012 |  |  |
| GSE21354 | hsa05214 |  |  |
| GSE3467 | hsa05216 |  |  |
| GSE3585 | hsa05414 |  |  |
| GSE3678 | hsa05216 |  |  |
| GSE4107 | hsa05210 |  |  |
| GSE5281\_EC | hsa05010 |  |  |
| GSE5281\_HIP | hsa05010 |  |  |
| GSE5281\_VCX | hsa05010 |  |  |
| GSE6956AA | hsa05215 |  |  |
| GSE6956C | hsa05215 |  |  |
| GSE781 | hsa05211 |  |  |
| GSE8671 | hsa05210 |  |  |
| GSE8762 | hsa05016 |  |  |
| GSE9348 | hsa05210 |  |  |
| GSE9476 | hsa05221 |  |  |
| GSE1145 | hsa05414 |  |  |
| GSE14924\_CD4 | hsa05221 |  |  |
| GSE14924\_CD8 | hsa05221 |  |  |
| GSE16759 | hsa05010 |  |  |
| GSE23878 | hsa05210 |  |  |
| GSE24739\_G0 | hsa05220 |  |  |
| GSE24739\_G1 | hsa05220 |  |  |
| GSE32676 | hsa05212 |  |  |
| GSE4183 | hsa05210 |  |  |
| GSE7305 | hsa05213 |  |  |

| GEO.ID | KEGG.ID | SET.VIEW | PATH.VIEW |
| --- | --- | --- | --- |

(Page generated on Wed Jul 15 11:18:10 2015 by ReportingTools 2.9.1 and hwriter 1.3.2)
