# Supplementary material for: Bioconductor’s EnrichmentBrowser: seamless navigation through combined results of set- & network-based enrichment analysis
Source: BMC Bioinformatics. 2016 Jan 20;17:45. doi: 10.1186/s12859-016-0884-1 (PMC4721010; doi:10.1186/s12859-016-0884-1)
Supplement: Supplementary file 4 — GEO2KEGG target pathways. Unzip and open the contained index.html in the browser to view the contents of this file (tested with Firefox 39.0). ZIP 4597.76 kb [file 12859_2016_884_MOESM4_ESM.zip › reports/index.html]

KEGGdzPathwaysGEO - Table of Results


## KEGGdzPathwaysGEO - Table of Results

| GEO.ID | KEGG.ID | SET.VIEW | PATH.VIEW | ORA.RANK | GSEA.RANK | SAFE.RANK | SAMGS.RANK |
| --- | --- | --- | --- | --- | --- | --- | --- |
| GEO.ID | KEGG.ID | SET.VIEW | PATH.VIEW | ORA.RANK | GSEA.RANK | SAFE.RANK | SAMGS.RANK |
| GSE1297 | hsa05010 |  |  | 85.5 | 2.4 | 5.3 | 17.2 |
| GSE14762 | hsa05211 |  |  | 24.3 | 16.2 | 15.2 | 25 |
| GSE15471 | hsa05212 |  |  | 51.1 | 14.7 | 54.3 | 50 |
| GSE16515 | hsa05212 |  |  | 7.3 | 11.6 | 18.9 | 3.8 |
| GSE18842 | hsa05223 |  |  | 13.1 | 94.4 | 27.6 | 100 |
| GSE19188 | hsa05223 |  |  | 32.3 | 82.3 | 69.8 | 100 |
| GSE19728 | hsa05214 |  |  | 17 | 61.1 | 24.6 | 2 |
| GSE20153 | hsa05012 |  |  | 100 | 51.9 | 23.2 | 20.3 |
| GSE20291 | hsa05012 |  |  | 100 | 38.1 | 32.1 | 43.4 |
| GSE21354 | hsa05214 |  |  | 4.3 | 53.2 | 2 | 4 |
| GSE3467 | hsa05216 |  |  | 50.2 | 69.2 | 44.3 | 7.7 |
| GSE3585 | hsa05414 |  |  | 71.8 | 60.9 | 81.9 | 22.2 |
| GSE3678 | hsa05216 |  |  | 55.7 | 66.9 | 36.1 | 1.4 |
| GSE4107 | hsa05210 |  |  | 49.4 | 14.8 | 25.3 | 5.3 |
| GSE5281\_EC | hsa05010 |  |  | 6.9 | 22.8 | 26.1 | 10.8 |
| GSE6956AA | hsa05215 |  |  | 100 | 6.8 | 35.1 | 13.2 |
| GSE6956C | hsa05215 |  |  | 100 | 20.8 | 12 | 13.5 |
| GSE781 | hsa05211 |  |  | 100 | 41.1 | 88.6 | 27.3 |
| GSE8671 | hsa05210 |  |  | 23 | 62.6 | 8.1 | 100 |
| GSE8762 | hsa05016 |  |  | 100 | 38.9 | 18.1 | 10.5 |
| GSE9348 | hsa05210 |  |  | 27.6 | 38.2 | 31 | 33.3 |
| GSE9476 | hsa05221 |  |  | 4.8 | 58.7 | 17.6 | 2.9 |
| GSE1145 | hsa05414 |  |  | 74.7 | 16 | 86.9 | 16.7 |
| GSE14924\_CD4 | hsa05221 |  |  | 2.1 | 6 | 3 | 5 |
| GSE14924\_CD8 | hsa05221 |  |  | 8.3 | 20.2 | 8.8 | 2.7 |
| GSE16759 | hsa05010 |  |  | 100 | 9.7 | 19.4 | 27.3 |
| GSE23878 | hsa05210 |  |  | 35.7 | 69.5 | 8.6 | 16.7 |
| GSE24739\_G0 | hsa05220 |  |  | 45.5 | 34.6 | 5.4 | 1.3 |
| GSE24739\_G1 | hsa05220 |  |  | 26.5 | 3.6 | 1.4 | 0.6 |
| GSE32676 | hsa05212 |  |  | 17.2 | 40.6 | 1.6 | 100 |
| GSE4183 | hsa05210 |  |  | 43 | 23.2 | 66.9 | 11.9 |
| GSE7305 | hsa05213 |  |  | 8.8 | 32.5 | 1.7 | 25 |

| GEO.ID | KEGG.ID | SET.VIEW | PATH.VIEW | ORA.RANK | GSEA.RANK | SAFE.RANK | SAMGS.RANK |
| --- | --- | --- | --- | --- | --- | --- | --- |

(Page generated on Tue Jul 14 20:42:45 2015 by ReportingTools 2.9.1 and hwriter 1.3.2)
